# Well-being as a tool to improve productivity in existing office space: Case study in Alexandria, Egypt

**DOI:** 10.12688/f1000research.133199.1

**Published:** 2023-06-12

**Authors:** Miral Hamadah, Ahmed ElSeragy, Sally ElDeeb

**Affiliations:** 1 Department of Architectural Engineering & Environmental Design, Arab Academy for Science Technology and Maritime Transport, Alexandria, Alexandria Governorate, 21500, Egypt; 2 School of Engineering, University of Lincoln, Lincoln, England, 19352, UK

**Keywords:** WELL, Rating System, well-being framework, Sick Building Syndrome, post-pandemic office buildings design standards, designing for occupants, Enhancing office productivity, Productivity design standards, Healthy Buildings Design

## Abstract

**Background:** The green building industry has significantly impacted the construction market, providing various sustainable solutions for the community. However, conventional green building standards have yet to adequately address occupant health and well-being, leading to challenges with performance. This has caused many businesses to take note of the latest report from the Bureau of Labour Statistics, which indicated that productivity in the US has dropped by the sharpest level since the 1940s [1]. Addressing these issues, organisations like International WELL Building Institute (IWBI) developed WELL Building Rating System (WELL), prioritising occupant health and well-being as critical components for improving performance and avoiding potential vulnerabilities brought about by sickness or pandemics. For this reason, this study will explore how to improve employee productivity within office buildings by bettering their overall health and well-being.

**Methods:** The methodology is designed to collect data from traditional office design, new trended successful office designs, and the WELL Building Rating System to understand healthy building design. Additionally, using DesignBuilder computer software simulates natural daylight, ventilation, and thermal heat gain in the case study to compare implementation results to the base case result.

**Results:** Showing thermal comfort, ventilation, and natural daylight significantly influence employees’ productivity. Implementing conducted design features from WELL achieved an average of 20.2%-35.6% decrease in thermal gain throughout the year, a 20% increase in airflow, an average 2.4%-6.5% decrease in Air temperature, enhanced temperature distribution by 7%, and direct sunlight minimum reduction by 9% in Winter and maximum 21.9% in Spring.

**Conclusion:** Our research analysed that new design features in famous office buildings positively impact employee productivity. We particularly examined the features outlined by WELL Building Rating System to identify the most influential factors affecting occupant productivity. The results of this study informed recommendations for enhancing productivity in existing office buildings in Alexandria, Egypt.

## 1. Introduction

The operation and construction of current and new buildings negatively impacted the environment for countless years. In addition to the 1970s energy crisis, builders and building owners were encouraged to turn to green building rating systems to assist them in designing more environmentally conscious buildings and communities. ^2^ However, most of the well-known green building standards that surfaced due to this movement were solemnly focused on achieving sustainability in buildings to provide a better environment while inadequately covering the matter of occupants’ health and wellness, as it can be a dominant factor for poor productivity and health of the society, especially in the business field as employees performance is essential. ^1^^,^^3^

Thus, another movement started in 2014 that included human health and well-being and how buildings affect occupants as a new factor to the green movement equation. ^3^ That is when International WELL Building Institute (IWBI) created the WELL Building Standard, the first system of its kind that tracks, measures, and certifies how building features affect the people inside. ^4^

Studies indicate that 90% of people’s time is spent in enclosed spaces, which impacts their health. During this time, they can be exposed to multiple pollutants, such as air pollution, inadequate lighting and ventilation, toxic materials indoors, uncomfortable temperatures, and disregarding mental health and the well-being of their social and community environment, all of which can be up to five times higher than average outdoor concentrations, ^5^ leading to negative short-term and long-term well-being and health outcomes that are held responsible for employees’ poor productivity and health vulnerability to pandemics in the workspace, ^6^^,^^7^ recently followed by the rise of the pandemic (COVID-19), which urged the need to change how we operate. Meanwhile, architects on a global scale started reconsidering current building design strategies and considering updating existing building standards to alleviate vulnerability to future pandemics and increase productivity and well-being. ^8^^,^^9^

While recent research has found that Air Quality, Thermal Comfort, and Natural Light are the three highest factors affecting office productivity, this is a significant finding as it shows that these factors directly impact employee performance. The findings suggest that companies should prioritise improving air quality, providing adequate thermal comfort, and increasing natural light in their office spaces to maximise staff productivity. Additionally, this data can help inform businesses about office design and layout decisions to create an environment conducive to employees’ well-being and success. ^10^

### 1.1 Research problem

The problem is that most office buildings in Egypt are closed, with inadequate operable windows, natural light, and thermal comfort. This leads to vulnerability to pandemics and a lack of motivation and productivity. ^11^ All caused by the fact that architecture development is unsynchronised with building occupants’ well-being and mental health, which should be considered a significant factor for a healthy community and sustainable environment, especially for the post-pandemic future. ^11^

### 1.2 Research aim

This paper aims to direct architects and construction firms in Egypt to include the well-being of occupants as an essential key factor of building design to connect occupants’ well-being to sustainability in design. To achieve this connection and complete the deficiency in the design criteria between architecture and occupant well-being, a thorough study of the WELL Building Rating System is submitted to resolve occupants’ issues within buildings in Egypt, along with analysing standard office design strategies and new trending office designs. To reach the paper’s aim, the following objectives will be achieved: Study and analyse the new WELL Building Standard rating that seeks the health and well-being of people, and understand all new categories developed by IWBI to develop a strategy to solve problems and boost efficiency in office buildings in Egypt.

### 1.3 Methods

The research focuses on the well-being of occupants in existing office buildings in Alexandria, Egypt, and highlights the Cairo Petroleum Complex office building as a case study.

Through analysing and studying the traditional and new design criteria of office design, we explore two famous successful office buildings, the Googleplex HQ and the Amazon Spheres, to analyse their productivity-derived design criteria in which we conduct their productivity derived design criteria.

The methodology is designed to conclude a checklist to enhance the productivity and well-being of occupants through studying the WELL building rating system concepts and features to conduct the design derived features that affects occupants’ health and productivity. Thus, we focus on six concepts: air, light, thermal comfort, movement, sound, and mind to conduct the features applicable on office buildings in Egypt. Next, we study three certified offices, which are the Centre of Sustainable Landscapes office building, the American Society of Interior Designers HQ office, and the 425 Park Avenue office building to analyse the impact of WELL certification on the employee’s productivity and understand the application of WELLs concepts in office buildings to conduct a summarised checklist to implement on the case study building “Cairo Petroleum Complex” in Alexandria/Egypt.

The methodology of this study used DesignBuilder computer software to simulate natural ventilation, lighting, and thermal comfort in the case study building in two stages:

First, simulation in the base case:

To ensure that the results obtained through the DesignBuilder software can be replicated, it is important to follow a systematic and rigorous process:

1. **Data collection:** Collect detailed and accurate data on the building’s geometry, construction materials, HVAC systems, lighting, occupancy, and other parameters that may affect its energy performance.
  Applied through: This data can be obtained through site visits, and other sources involves collecting detailed and accurate data on the building’s geometry, construction materials, HVAC systems, lighting, occupancy, and other parameters that may affect its energy performance; in this paper we obtained the data from a site visit to the case study building.
2. **Model creation:** Use the DesignBuilder software to create a detailed and accurate model of the building, considering all the relevant parameters and inputs.
  Applied through: This involves inputting the data collected in step 1 and configuring the various parameters of the simulation, such as building orientation, window size and placement, in this paper we create a model of the base case five floor building of 3370 m ^2^ total construction area. The building is a rectangle shape of dimensions 60 m × 67 m and a 20 m building height with uninsulated exterior brick walls thickness of 20 cm and 2 cm interior and exterior plaster with the total u-value of 1.5 W/m ^2^K and 4 m single floor height. The building main structure is reinforced concrete with uninsulated fixed curtain wall elevations with no shades externally and uses mechanical ventilation system. The building roof is flat 20 cm reinforced concrete and insulated with four single glazed skylights of 240 m ^2^ total area above the main court.
3. **Calibration:** Calibrate the model by comparing its predicted energy performance against the actual energy consumption data for the building and adjusting the inputs as necessary to improve the model’s accuracy.
  Applied through: This process involves iteratively adjusting the input parameters, running the simulation, and comparing the results against actual data until the model is accurately calibrated that is not available in this case so Appling step 5.
4. **Sensitivity analysis:** Conduct sensitivity analyses to determine the sensitivity of the model’s results to changes in the input parameters and identify the most important variables that affect the building’s energy performance.
  Applied through: This step helps identify the most important variables that affect the building’s energy performance and provides insights into how the model can be improved.
5. **Verification:** Verify the accuracy of the model by comparing its predicted results against independent data sources, such as published literature or other validated models.
  Applied through: This step helps ensure that the model is reliable and can be trusted to provide accurate predictions. In this paper we validate the air temperature of the base case simulation to that of Alexandria weather and use the “correlation coefficient” parameter to validate the results as correlation coefficient (R2) should range between -1 and 1, in this case, the result was 0.993 which is within the acceptable range of the correlation coefficient -1 and 1, thus indicating that the base case results are reliable.
6. **Documentation:** Document the model creation process, including the input data, assumptions, and any modifications made during the calibration and verification steps.
  Applied through: This documentation should include the input data, assumptions, and any modifications made during the calibration and verification steps. Proper documentation also helps to ensure that the model can be updated and maintained over time as necessary.

By following these steps, it is possible to create an accurate and reliable model of a building’s energy performance using the DesignBuilder software, and to ensure that the results obtained can be replicated and verified.

Second, simulating daylight, thermal heat gain, and natural ventilation after implementing the conducted criteria on the case study building to compare the impact results with the base case results.

Data input: Cairo Petroleum Complex base case, EGYPT region, energy code ASHRAE 90.1-2007, location simulation using weather data “EGY_AL ISKANDARIAH_ ALEXANDRIA_ETMY”, the yearly design temperatures using 0.4% dry-bulb cooling design temperature with maximum value 33.2 OC, minimum value 27.1, and a Coincident wet-bulb temperature value is 22.3 OC, the climate zone used in ASHRAE 2B.

The building activity template is “office buildings” as the ASHRAE 90.1 Settings for heating source is “fossil fuel”, the occupancy density (people/m ^2^) = 0.05 based on the building survey.

For the Environmental control the heating set point temperatures for heating is 20.0 °C, and heating set back is 13.0 °C, the cooling set point temperatures cooling is 26.0 °C, and the cooling set back 32.0 °C. the computers and office equipment supplied to each zone according to the building visit.

The building constructions for the exterior wall the U-value equals 2.094 (W/m ^2^-K), and for the internal partitions equals 1.490 (W/m ^2^-K), and the typical floor equals 2.353 (W/m ^2^-K).

For the opening the external windows layout using wall façade types for 40% vertical glazing ASHRAE 90.2 Appx with a single layer of generic Clear 6 mm glass panel.

The lighting system through ASHRAE classification is space-by-space method with HVAC template is fan coil unit (4-Pipe), Air cooled.

All these parameters also applied for the post implementation scenario expect proposing a shading device (Vertical and horizontal Louvers) for the curtain walls windows as described and switching single glazing to double glazing additionally adding 30% operable windows externally and internal windows to allow cross ventilation.

### 1.4 Study design

This paper will follow four parts:

1.4.1 Literature review: Involves studying the definition of well-being, what are the main points that lead to achieving it and learning the WELL Building Rating System, which focuses on the study of people and how to make them thrive, to reach a design solution to improve the performance, productivity, and health of building occupants.

1.4.2 Qualitative study: Performed to see how different buildings and companies implement different design strategies to improve the overall health performance of their employees and how it all reflects on their productivity. The analytical part will include analysing the WELL framework and studying existing office building examples to understand how to improve the design strategies in Egypt to increase productivity.

1.4.3 Quantitative study: Concluding a checklist to be implemented in Egypt office spaces designed to improve productivity and well-being of employees and implement it in the case study office.

1.4.4 Practical part: Implementing the design strategy on a single office in the chosen case study building, “Cairo Petroleum Complex” office building in Alexandria/Egypt, using the computer software DesignBuilder to compare the simulation results to the same office pre-implementation. DesignBuilder is a paid computer software that has a free 30-day trial period. Alternatively, a free version of TRNSYS is available to use.

## 2. Results

### 2.1 Developed office design standards

According to the “Health, Wellbeing & Productivity in Offices” research article published by the World Green Building Council (WorldGBC), among other research concluded that the critical factors in designing powerful office space to boost productivity and well-being are location, layout, size, appeal, atmosphere, and the dimension of the area. ^12^^–^^15^

**Figure 1.**
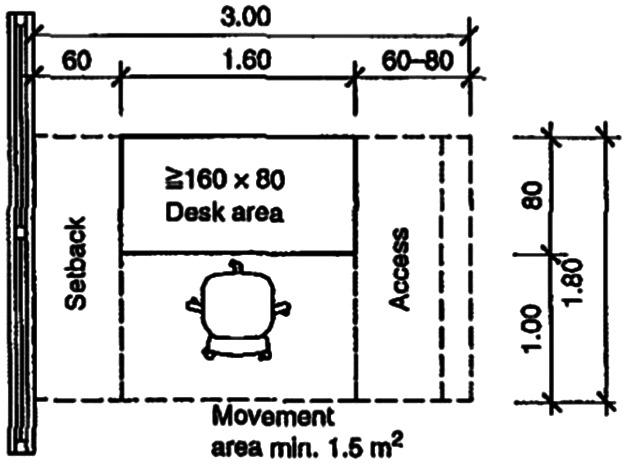
The minimum space required for a single workstation in meters giving a total area of 5.4 m ^2^: Neufert Architects’ Data Fourth Edition, 2012.

**Figure 2.**
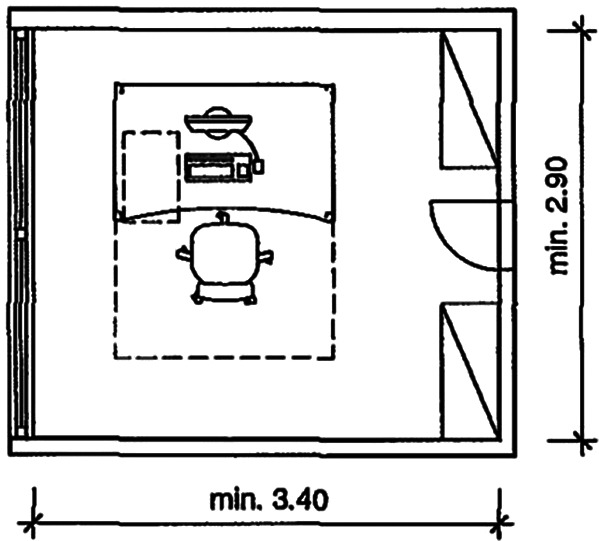
An example of a single office room dimension in meters giving a total area of 10 m ^2^ per office room: Neufert Architects' Data Fourth Edition, 2012.

In 2012, workspace design studies concluded that the average office space per employee was around 5.4 to 6.5 m ^2^ and approximately 10 m ^2^-16.4 m ^2^ for an office room (see Figure 1 and Figure 2). ^16^

**Figure 3.**
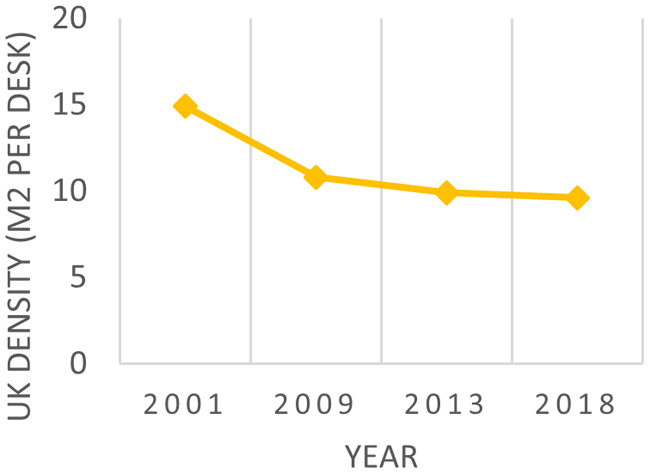
Benchmark density in UK offices over time: BCO the future of UK office density, 2022.

Other studies, like the research done by the British Council for Offices (BCO), concluded that the average density in offices was around 14.8 m ^2^/employee in 2001 and decreased to 9.6 m ^2^/employee in 2018 (see Figure 3). ^17^ From 2000 to 2012, the average space per employee decreased by about 21% due to the growth in flexible workspaces and remote working trends. ^18^

**Figure 4.**
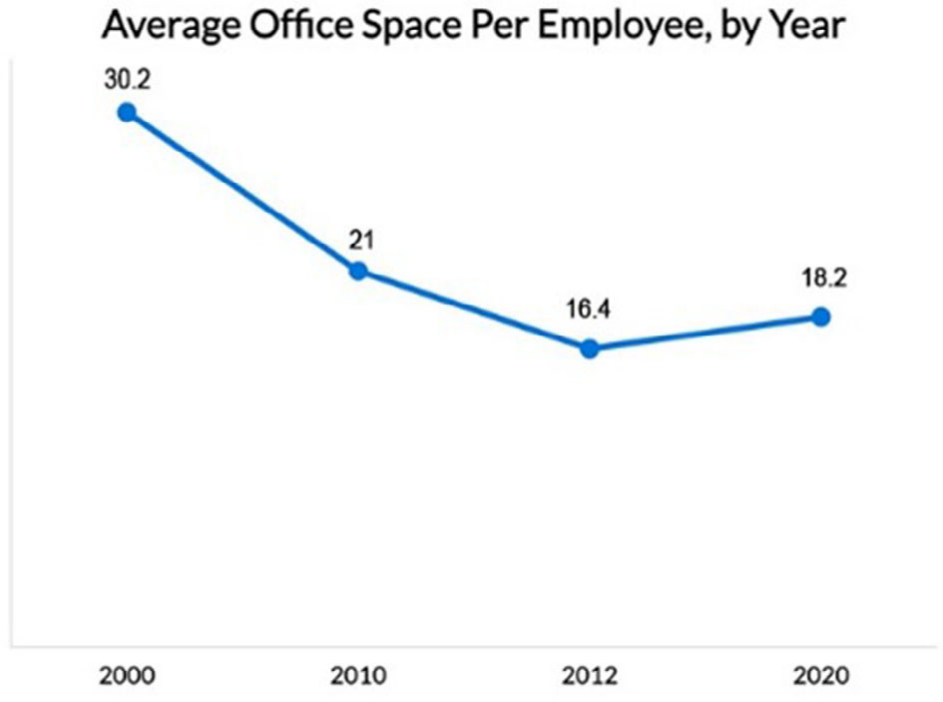
Over the years, the decrease in square meter area in Office space per employee: McMahon, 2023.

**Figure 5.**
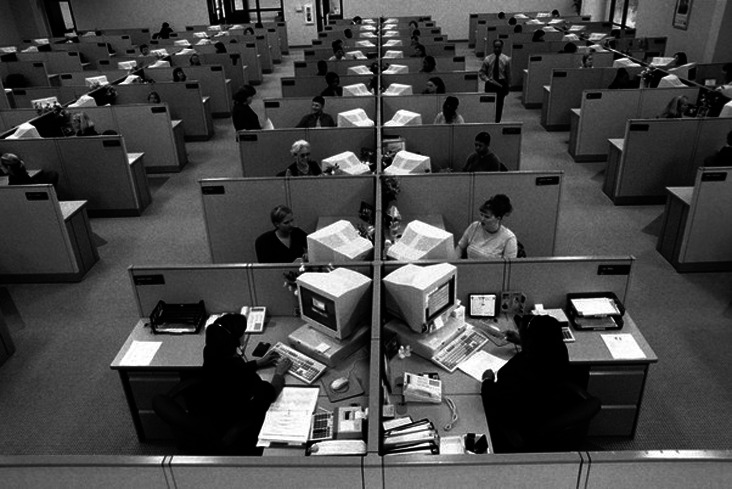
The common office cubicles were created in 1967 by the designer Robert Propst, who worked at the office-furniture firm Herman Miller: The Wall Street Journal.

**Figure 6.**
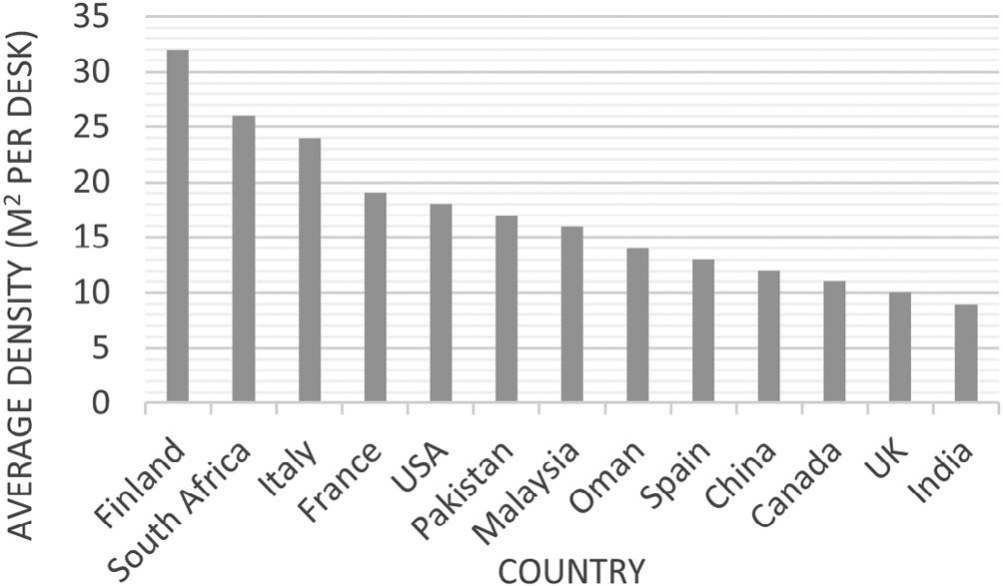
Comparing average density offices between countries (from 2010 to 2022): BCO the future of UK office density, 2022.

Between 2010 and 2012, the area per employee decreased from 21 m ^2^ to 16.4 m ^2^ (see Figure 4). ^16^ At the start of the 21st century, this number was as high as 30.2 m ^2^. ^18^ By contrast, with employers concentrating on returning to the office while maintaining physical distance in response to the COVID-19 pandemic, the amount of space per worker began increasing again. ^16^ While there is no single size that fits all types of businesses, US research showed that in 2020 the average area per employee increased from 16.4 m ^2^ to 18.2 m ^2^ (see Figure 4) (considering various needs depending on the kind of work done in that space). ^16^ This considers dedicated desk space and surrounding spaces such as meeting rooms and shared areas. ^19^ Additionally, The British Council for Offices suggests a generous allocation of space based on people rather than desks to satisfy companies’ current requirements for maximising staff performance and comfort by providing a range of settings at work. ^20^ This report points out that with a 10-12 m ^2^ ‘Sweet Spot’ for each person, most common workspace issues like overcrowding and noise pollution can be addressed. On the contrary, higher office densities with less than 8 m ^2^ per person are more likely to cause complications and negatively affect occupant comfort, well-being and performance for most businesses ^20^ (see Figure 5 and Figure 6).

It was also believed that the way density is measured can vary depending on the degree of enclosure (e.g., open plans and screens/partitions, etc.) and the office design and desk layout all influence the effect of office density on employees’ productivity. ^17^

Considering the recommendation to increase the size of the average workspace per person by 50% as a precautionary measure against COVID-19. ^19^ Some companies followed the ‘Hybrid work schedule’ method (assigning half the employees to work remotely and switching shifts the following week) while maintaining the same office footprint. In contrast, others chose to apply the distancing in a larger office or reduction of employees. ^21^

At the start of the 21st century, companies realised the significance of their office setting. Now, when designing a workspace, there are some standard key considerations ^13^^,^^14^:

• Location: amenity-rich central location. ^22^^–^^24^
• Appeal/Utilization (e.g., relaxed dress codes, a splash of colour, natural light, greenery, soft furnishing, Etc). ^23^^–^^26^
• Well-being (biophilic design, the distance between desks, areas for socialising, opportunities for fresh air, self-care amenities, private mothers’ rooms, fitness amenities, things that are meaningful for the users). ^9^^,^^22^^,^^23^^,^^27^
• Flexibility (e.g., open plan with flexible seating areas, Open and private offices, staff rotating schedules for remote and office attendance, ETC). ^9^^,^^21^^,^^22^^,^^25^^,^^27^
• Designing the office by noise level (e.g., Public, Semi-Private, Private, adding a space for employees to unwind, ETC). ^21^^,^^25^^,^^26^
• Good Design (Passive solutions, shading, natural ventilation, and daylight when possible). ^9^^,^^24^
• User in control (giving occupants control over their indoor environment). ^24^

With this in mind, we look at some of the supreme paradigms of successful office buildings that applied unusual design strategies to enhance employee productivity.

### 2.2 Examples

Googleplex

**Figure 7.**
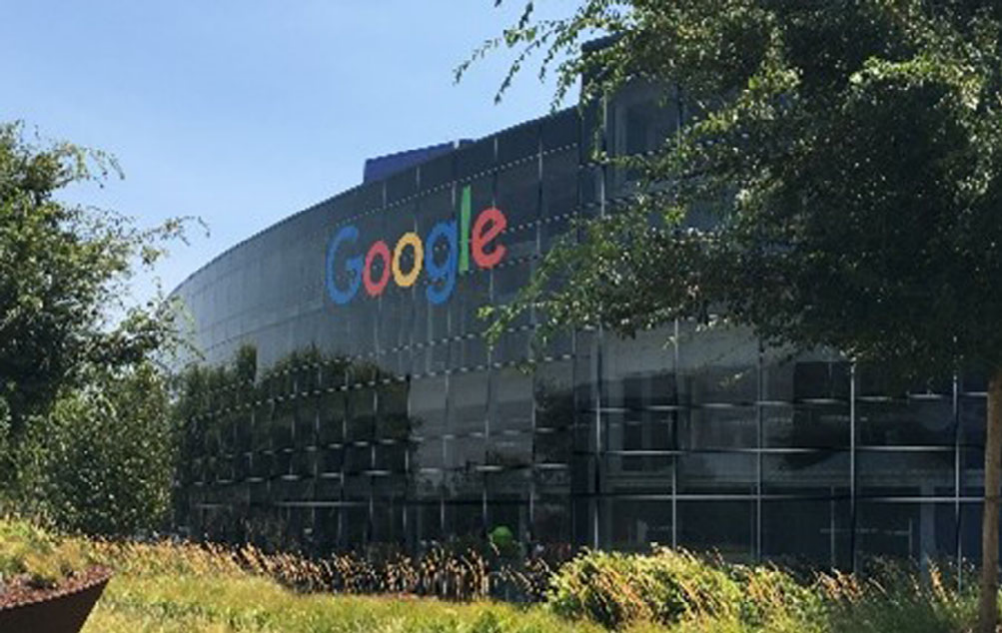
The elevation of the Googleplex building in California: Wikipedia.

With around 190 km ^2^ footprint of office space in California, USA (see Figure 7). Clive Wilkenson Architects Firm designed the building to mimic the university campus feel and merge the idea of the workplace with the experience and knowledge found within the educational environment. By applying 13 different office settings, the building components were proven effective in boosting employees’ well-being, ergo productivity. Google aimed to decrease everyday concerns and stress by providing all daily needs in one campus. ^28^ The building registered a 31% increase in revenue in 2013, ^29^ which means their philosophy benefited the company (see Table 1 for building content).

**Table 1.** Summarised Googleplex Building design features aiming to enhance the well-being and productivity of their employees: Author.

Design features	Additional detail
Fitness	Onsite gym, swimming pools, tennis courts, soccer fields, outdoor spaces for activities like frisbee, yoga, etc.
Entertainment area	Ping pong, billiard, foosball table, arcade.
Easy transportation	Travelling between amenities by Google coloured bikes, scooters, and electric cars.
Self-contained pods	Used by staff members to kick back and relax or distance themselves from the surroundings to get down to work on some heavy tasks.
Healthcare	Onsite medical staff.
Self-Care	Massage services, Haircut salon, laundry room and dry cleaner.
Nutrition	Three free meals daily for the whole staff and free snack bars. Vegetables and fruits eaten in the cafeteria are grown inside the gardens in google parks.
Eateries and cafes	Forty Restaurants and Cafeterias, in addition to every single workplace is within walking distance of an entire snack room.
Biophilic Design	Glass offices, curtain walls and skylight for natural light, soundproofing, and open spaces.
Various meeting room settings	Soundproof meeting rooms, open and closed meeting rooms.
Natural light	Courts, skylight, curtain walls.
Enhancing Productivity	Whiteboards walls for creative ideas and anonymous jokes.
Flexible workspace	Campus-style layout, glass offices, private offices, open plan desks, open spaces, and various seating areas.
Natural elements	Green elements and water elements.

Amazon Spheres

**Figure 8.**
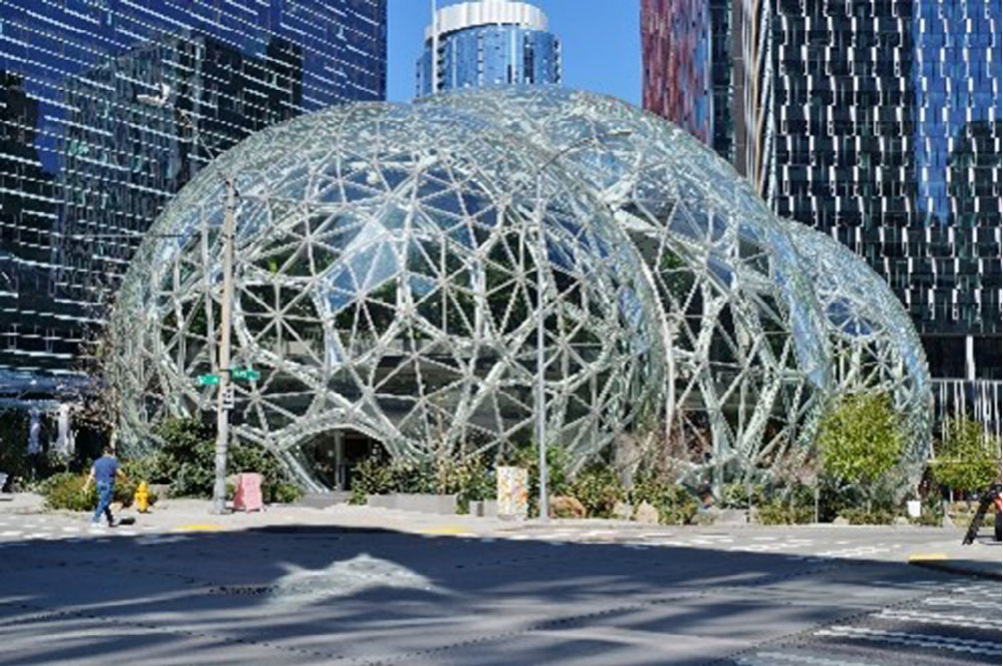
The Amazon Spheres three glass domes that are covered in pentagonal hexecontahedron panels: Wikipedia.

At 299.6 m ^2^ footprint located in Washington DC, USA (see Figure 8). ^30^ The Amazon Spheres was designed by NBBJ with the idea of mimicking the forest atmosphere while still being in touch with the urban areas’ comfort and luxury. After doing their research, they found that nature decreases stress, reduces cortisol levels and improves focus, and that’s how they came up with the building’s concept (see Table 2 for buildings content). ^30^

**Table 2.** Summarised Amazon Spheres Building design features aiming to enhance the well-being and productivity of their employees: Author.

Design features	Additional detail
Plant wall	The largest indoor living wall in the country is around 19m tall and 15m wide and hosts 25k individual plants. The building hosts 40k different varieties of plants (700 plant species). ^31^
Flexible seating	Variety of seating areas and open spaces.
Different Meeting rooms settings	Open and closed meeting areas. Canopy walls for walking meetings. Conference room surrounded by vines for privacy (AKA the bird cage).
Circadian solution	Lights stimulating daylight.
Flexible workplace	Places intended to create different working environments.
Curtain wall	The Spheres’ Catalans support 2,643 panes of glass; the outside structure was intended to look like vines or spider webs.
City view	A ring path circles inside the building, looking out to the city so employees can get the best of both worlds. The fourth floor is under the sky and has a 360-degree city view.
Natural Light	Court, skylight.
Eateries	Cafes and breakrooms.

By analysing the past examples and studying Table 1 and Table 2, we conduct a replicated design criteria followed by the two companies to boost employee productivity. Thus, the following table concluded the best design criteria for office buildings to increase the productivity and well-being of their employees (see Table 3).

**Table 3.** Replicated productivity-enhancing design features achieved in the analysed examples: Author.

Design features	Additional details
Natural Elements	Natural elements like greenery improve air quality, remove pollutants, and psychologically improve employees’ well-being. ^32^
Air Quality	The simulated office work performance improves by an average of 1.5% when the dissatisfaction with air quality is decreased by 10%. This can be achieved by reducing air pollution or increasing the ventilation rate without changing the interior climate conditions. ^33^
Open areas	Human stress levels are strongly affected by the surrounding environment, which affects their well-being and productivity ^34^^–^^36^; studies show that open space characteristics, in contrast to closed offices and cubic cells, can positively reduce stress levels and improve health outcomes. ^34^^–^^37^ This can be applied through open-plan design, outdoor seating areas, etc.
Easy transportation between facilities	Suppose the company had more than one building. In that case, an easy transportation method between buildings should be provided, like bicycles, and an easy transportation route to and from the building near a bus stop.
Terrace	Alternative places for nomadic work must provide at least two terraces per building.
Natural light	Humans are influenced by light both physiologically, psychologically, and behaviorally. Studies have shown that light can affect our physical well-being, alertness, and sleep quality. Additionally, natural light affects circadian rhythms, impacting the brain's cognitive performance. ^38^ This can be applied in the shape of curtain walls and skylights while considering proper shading elements.
Ventilation features	Using cross-ventilation, operable windows, courts, etc.
Flexible seating arrangement	Not assigning offices to employees and allowing workers to choose their seating preference daily.
Open offices	Supports collaboration, learning, and sharing ideas between employees when needed.
Closed offices	To provide privacy for employees when needed for more concentration and “head down” activities.
Open meeting rooms	For quiet nomadic work and improvised team collaborations containing seating areas such as sofas and coffee tables.
Closed meeting rooms	Focused space for collaboration containing seating areas, whiteboard, and projector.
Conference room	Focused space for larger collaborative groups contains whiteboards, a projector, and a more prominent seating solution.
Courts, Atriums, Skylight	A good rule of thumb for gracious natural light entrance and proper ventilation.
Fitness and Sports Facilities	Including a gym, football, basketball, or tennis court.
Eateries	A break space for lunch and coffee breaks also enables the chance to encounter collaborations.
Relaxation areas/Mind breakrooms	A space holding natural elements (e.g., plants, fountain, etc.) and seating areas for employees to unwind and lower stress levels.
Mother break rooms	For working moms, including lactation rooms and child day-care can shorten the parental leave period.
Self-care	Including spa rooms and meditation areas.
Entertainment facilities	A shared area with entertainment options (pool table, game room, etc.) for employees to unwind from work stress.
Healthcare facilities	Most workers tend to request an absence leave to get a medical check-up, including a healthcare unit (like medical staff or a clinic) in the building can prevent unnecessary leaves.
Library	A quite nomadic workspace.
Creative facilities to promote productivity	Wallboards, screens, and motivational signs.
Privacy areas/breakrooms	For concentrated solo work or a quick nap.

## 3. Occupants’ well-being ranking systems

The WELL Building Standard (WELL) created by the International WELL Building Institute (IWBI) is a cutting-edge rating system that considers energy use, water consumption, waste production and other environmental impacts, as well as several socioeconomic measures. This has helped lead to the increasing global importance of green building construction and design that works towards creating a workspace where employees can thrive. ^39^

WELL is a holistic approach that needs the equal effort of four aspects: Design, Operation, Behaviour, and People for it to succeed. WELL is designed to complement other top-tier green building standards while conducting thorough research into how the building environment can be improved for its occupants. As a result, projects are encouraged to seek dual certifications from both WELL and green building standards to achieve higher quality results.

### 3.1 Development of WELL

**Figure 9.**
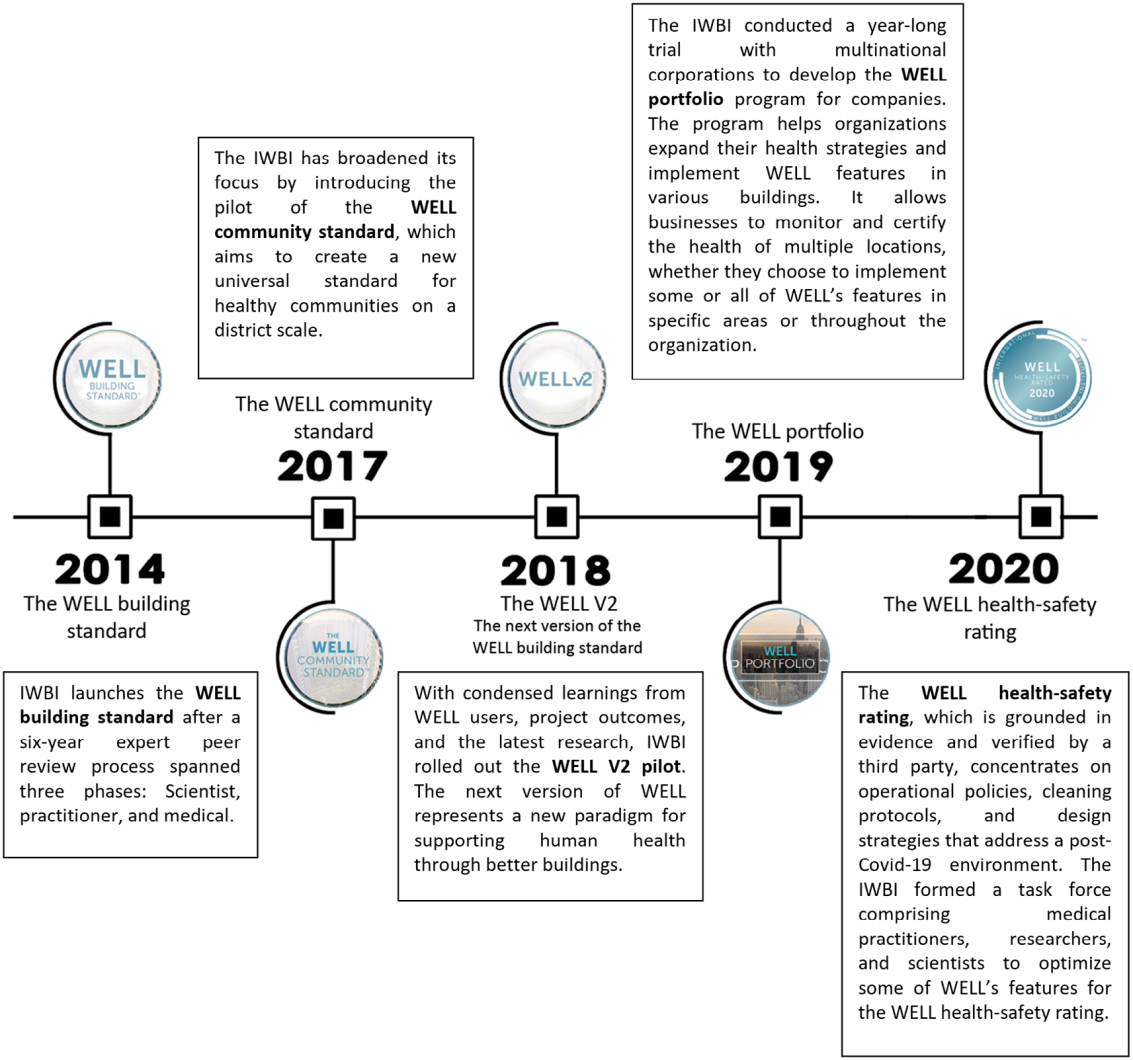
The timeline of WELL Building standard development: Author.

IWBI upgraded WELL V1 (consisting of seven concepts and three point-based scorings) to WELL V2 in 2018 (see Figure 9 for a detailed WELL timeline), which includes ten concepts and four point-based scorings. These include Air, Water, Nourishment, Light, Mind, Movement, Materials, Thermal Comfort, Sound and Community. ^39^

**Figure 10.**
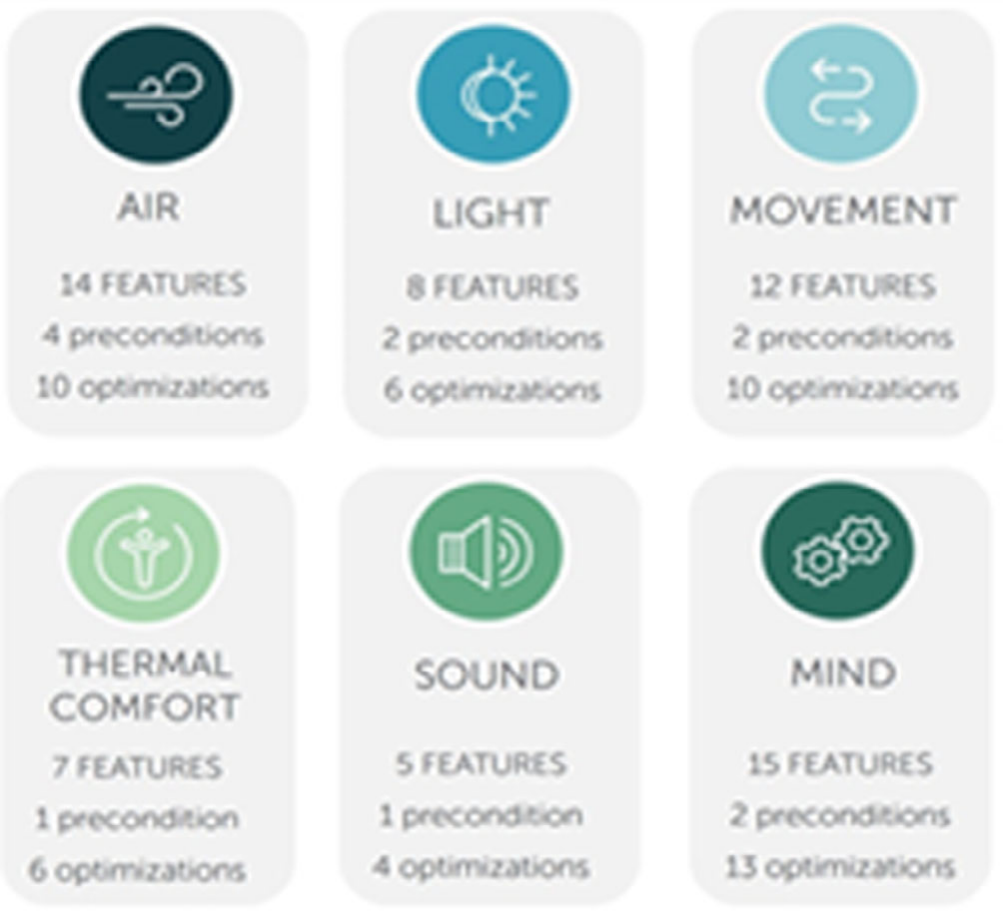
The WELL Building standard selected concepts for this paper with each concept’s number of features (divided into preconditions and optimizations). Source: IWBI.

This paper will discuss solely the concepts that have an architectural value to apply to the design strategies in Egypt (see Figure 10. For selected WELL concepts content categories).

The recent Global Burden of Disease study put household air pollution as the 10th highest cause of poor health for the world’s population, ^40^ making it one of the most important concepts within WELL. Light has also been shown to profoundly impact occupants’ moods and symptoms of depression. ^41^ For this reason, WELL looks to provide an environment where light can positively affect sleep quality and reduce circadian phase disruption to promote better moods and productivity.

The WELL Movement concept encourages physical activity, movement, and active living and discourages sedentary behaviour [ 1] from combating the global trend of physical inactivity. In 2016, more than 23% of adults were reported as physically inactive, ^42^ and a 2011 study suggested that adults worldwide sit for 3-9 hours each day. ^43^ This sedentary behaviour has been linked to obesity, type II diabetes, cardiovascular risks, and premature mortality. ^44^^–^^48^ If just 10% of this physical inactivity was reduced globally, it is estimated that at least 500 000 deaths could be prevented annually; reducing physical inactivity by 25% could result in over 1 million fewer deaths per year. ^48^

The WELL Thermal Comfort concept strives to maximise thermal comfort and accommodate individual preferences, resulting in higher productivity levels using improved HVAC system design and control. Where we live and work can be significantly impacted by thermal comfort; thus, it is one of the most critical factors influencing building satisfaction. This includes individual motivation levels, alertness, focus, and mood. ^49^

Mental health is essential for individuals to reach their full potential, cope with everyday challenges, work productively, and give back to the community. ^50^ Unfortunately, mental health issues and substance abuse affect 13% of the global burden of disease, accounting for up to 32% of years lived with disability. ^51^ 1 in 6 adults suffer from common mental health conditions such as depression, anxiety, or substance abuse at any given moment, and over 30% will experience a mental health condition throughout their lives. ^52^ These conditions also profoundly impact the workplace - impeding daily productivity and costing the global economy $1 trillion. ^53^ The WELL Mind concept seeks to address this issue by implementing design strategies that support cognitive and emotional health through prevention and treatment efforts - this can significantly improve individuals’ short-term and long-term mental well-being.

Finally, the WELL Sound concept prioritises occupants’ health and comfort by identifying and addressing acoustical parameters in built environments to improve the user experience.

Following this general introduction to WELL Building Standards’ six Architectural based concepts is the conducted checklist to implement in office buildings in Egypt.

### 3.2 Conducted checklist

The WELL Building Standard centres around ten core concepts, each contributing to creating a healthy living and working environment. Six of these ten concepts are particularly relevant to architecture design: air, light, movement, thermal comfort, sound, and mind. These concepts will be discussed in more detail below:

**Air**

Choosing four key features from the Air concept, each with its own set of points, ensures these goals are met.

The first feature is Enhanced Ventilation, where automated air conditioning systems should supply conditioned air through individual diffusers positioned 0.8 m above the occupants’ heads.

The second feature is Operable Windows, which provides access to natural ventilation when possible. At least 75% of occupied spaces should have operable windows of at least 4% of the floor area, and these should be designed with universal access in mind so they can be operated easily without tight grasping or twisting of the wrist.

Pollution Infiltration Management is the third feature, where the entryways of regular entrances (excluding terraces) should use entryway design elements such as grilles, grates, slots, or roll-out mats that have widths of at least 3 m and length in the circulation direction.

Finally, Source Separation examines separating rooms with high-volume printers, copiers, and humidity using automatic operating doors and negative-pressure exhaust fans that redirect outside air into higher-pressure areas. ^39^

**Light**

This concept is centred around the idea of light exposure with six selected features. The first feature is Light exposure focusing on the interior layout. 30% of all occupied areas must be within 6 meters of envelope glazing, and common areas must have seating for at least 15% of regular occupants, with a 5-meter distance between seatings and envelope glazing for 70% or more of said seating.

The second feature is Visual Lighting Design - 90% or more of space types in the project area must meet illuminance thresholds based on their purpose (offices need 320 lux at task surfaces while lobbies, atriums, and transition spaces need a minimum of 110 lux at floor levels). Eateries, lounge, and restroom levels are required to achieve a minimum of 110 lux at the task surface).

Circadian Lighting Design is the third feature - meeting lighting requirements for day-active people such as applying light levels on vertical planes at eye level, achieving 4 hours (min. start by noon) of light over work surfaces at 45 cm height and 140 cm in the centre of all seating areas and kitchens.

Electric Light Glare Control follows this as the fourth feature - buildings needing strategies to manage glare from electric lighting either through luminaire, considerations that limit UGR values to 16 or lower, and luminance do not exceed 6,000 cd/m ^2^ between angles 450-900 from nadir; or through space consideration where UGR values must also be 16 or lower.

Daylight Design Strategies is the fifth feature - two options present themselves: 70% of workstations within 7.5 m from transparent envelope glazing with VLT > 40%, 15% minimum envelope glazing area, or 70% within 5 m with VLT > 40%, 25% minimum envelope glazing area; both facilitated by solar shading in manual mode controllable by occupants (opening throughout the working day), automated shading for glare prevention.

Last up is Occupant Lighting Control - ambient lighting systems should be in place per 60 m ^2^, one per 10 occupants’ zones; differing criteria if rooms are smaller than needed or occupancy is lower than allocated quota; plus supplemental lighting available controlled by occupants. ^39^

**Movement**

This concept focuses on creating a healthy and comfortable working environment. There are four selected features in this concept. The first feature is Ergonomic Workstation Design which requires a minimum of 25% of workstations to be adjustable by users to support standing and seated positions. This includes flexible device heights, chairs, anti-fatigue mats or impact-reducing flooring, toe space, and footrests/footrails.

The second feature is Circulation Network which looks at aesthetically designed staircases with music, artwork, light levels, access to daylight and natural design elements for each floor. Visible stairs should be promoted over elevators and escalators from the entry-level onwards.

The third feature is Facilities for Active Occupants which provides cycling infrastructure with short-term bike parking located 30 m from the entrance accommodating at least 2.5% of visitors, and long-term bike parking located within building boundaries accommodating at least 5% of occupants. Furthermore, within a 200 m walk distance from the building boundary, there must be showers, lockers and changing facilities available for every 0-100 regular occupants, plus one shower per 150 occupants for every 101-999 regular occupants and 8 showers plus 1 per 500 occupants for every 1000-4999 regular occupant as well as 16 showers plus 1 per 1000 occupant for more than 5000 regular occupants with a minimum of five lockers associated with each shower facility.

The last feature is Physical Activity Spaces and Equipment, which requires the provision of an indoor activity space with dedicated fitness facilities offering two types of exercise equipment that can be used by at least 5% of building users, as well as outdoor physical activity spaces such as green spaces like parks or trails, blue spaces like swimming areas, recreational fields or courts and fitness zones. ^39^

**Thermal Comfort**

This concept has six selected features that improve users’ thermal comfort.

The first feature is Verified Thermal Comfort. The first point under this feature is a Thermal Comfort Questionnaire - occupants must participate in an anonymous questionnaire, and the number of responses required depends on the number of occupants: if there are more than 45, then a minimum of 35% should respond, 20-45 requires 15%, and fewer than 20 requires 80%. The results of responses must also meet target satisfaction thresholds: 80% or 90%.

The second feature is Thermal Zoning. The first point is to Provide Thermostat Control for at least 90% of occupied spaces; temperature in each room must be controlled via a thermostat or digital interface accessible via a smart device; maximum size per thermal zone should not exceed 60 m ^2^ or 10 occupants. Sensors should be placed at least 1 metre away from exterior walls, doors, windows, direct sunlight, air supply diffusers, mechanical fans, heaters etc.

The third feature is Individual Thermal Control. It includes two points. The first point provides Personal Cooling Options such as rooms/thermal zones with adjustable thermostats connected to building cooling systems that one person can regularly occupy; desk/ceiling fans; mechanical cooling system chairs; any other solutions capable of affecting a PMV change of -0.5 within 15 minutes without changing PMV for other occupants. The second point lists Personal Heating Options such as rooms/thermal zones with direct user-adjustable thermostats connected to buildings heating systems that can only be regularly occupied by one person; electric parabolic space heaters; electric heated chairs/footwarmers; any other solutions capable of affecting a PMV change of +0.5 within 15 minutes without changing PMV for others.

The fourth feature is Radiant Thermal Comfort, with Implement Radiant Heating and Cooling being the only point - at least 50% of occupied areas should have radiant ceilings/walls/floors or radiant panels attached, covering half the wall/ceiling area minimum.

Fifth is Enhanced Operable Windows, where Provide Windows with Multiple Opening Modes has four points: At least 70% open so no more than 1.8 m above finished floor (1 window per room); 30% open with whole opening 1.8 m above finished floor (1 window per room), Operation controls min 1.7m above the finished floor and low openings used in mild/warm weather, high in cold weather.

The last feature is Outdoor Thermal Comfort which consists of two points: Manage Outdoor Heat where pedestrian pathways and building entrances must have tree canopies, awnings or other structures providing shade for ≥50%, parking spaces ≥25%, plazas seating areas and other outdoor areas covered between 25%-75%; Avoid Excessive Wind where 5 m/s not expected more than 5% hours yearly in seating areas and 10% on paths and parking lots while 15 m/s no more than 0.05% hours throughout the year across all areas. ^39^

**Sound**

This concept has three selected features, starting with sound mapping as the first feature, offering an Acoustic Design Plan. Sound barriers are the second feature, with Doors and Walls Sound Isolation Design as its primary point. Lastly, Impact Noise Management is the third feature, with Specify Impact Noise Reducing Flooring being its main point. ^39^

**Mind**

There are five selected features in this concept. The first feature is devoted to promoting mental health and well-being with a dedicated space for restoration and relaxation, as well as work policies allowing breaks.

Nature and Place is the second feature that establishes a connection to nature through materials, patterns, shapes, colours, images, or sounds. It also entails celebrating culture and the integration of art.

The Restorative Opportunities feature, which is the third feature, provides a nap space and policy with at least one acoustically and visibly separated environment in a designated quiet zone, plus one reclining furniture for every 100 employees.

The Restorative Spaces is the fourth feature and offers an environment considering specific criteria such as lighting, sound, thermal comfort seating arrangements, calming colours, textures, and forms.

Lastly, the Enhanced Access to Nature fifth feature guarantees that 75% of workstations and seating areas have views of indoor plants/water/natural elements, and 70% of outdoor spaces include plants or natural elements within 200 m walk distance from the rooms available to occupants. ^39^

### 3.3 Discussion of findings

Creating a productive office environment is essential for any business to succeed. After studying WELL concepts, other sources stated that the most critical factors for achieving success in office buildings are natural air, ventilation, and thermal comfort. ^10^^,^^54^ Good indoor air quality helps keep employees energised and productive while keeping air-borne diseases at bay. Quality ventilation systems help ensure that fresh air constantly enters the office, aiding in concentration and morale. Additionally, thermal comfort should be considered to prevent uncomfortable working conditions due to too hot or cold temperatures. All these elements combined create the perfect workspace environment where productivity can flourish. Thus, those are the three points that will be focused on in the design implementation.

### 3.4 Productivity

Employee productivity significantly impacts profits, yet it isn’t something that can easily be measured, and it’s not a one-size-fits-all rule to follow. Meanwhile, companies use three standard productivity calculation methods to measure productivity. This has nothing to do with what employers think of their employee’s performance because their opinion can be wrong.

One way to evaluate the impact of physical features on employee productivity is by collecting data such as physical features, outcome metrics (e.g., physical complaints) and HR department data (e.g., worker attitudes, performance data, absenteeism, medical costs, retention rates etc.), as well as financial directors’ data concerning revenue and financial metrics. These can be used to compare and calculate the overall effect on employee productivity.

Another method used is the direct use of the Labour Productivity Formula, a simple equation derived from the basic definition of productivity. It is output per unit input; you can use it to track productivity per individual, team, or even department. ^55^

$$\text{Productivity Formula}:\text{total output}\;\left(\text{Value of goods and services produced}\right)/\text{total input}\;\left(\mathrm{man}-\text{hour}\right)=\text{Labour Productivity}.$$

For example, an organisation produced goods or services worth $100,000 in 2000 hours. The output is worth $100,000, while the input is 2000 hours. Then, using these values in the formula, we get:

$$\text{Productivity}=\left(\text{Output}/\text{Input}\right)=\left(\text{\$}100,000/2000\;\text{hours}\right)=\text{\$}50\;\mathrm{per}\;\text{hour}.$$

Of course, in this equation, we calculate the work hours, not the paid hours (as paid hours can mean vacation pay and sick leave salaries, too). ^56^

Next is the Percentage of Goals Met method; companies’ productivity levels are synonymous with the number of goals employees accomplish. By calculating the percentage of goals met, we can determine where the workforce stands regarding efficiency. ^57^

$$\text{Percentage of Goals}\;\mathrm{Met}=\left(\text{Actual Output}/\text{Goal}\right)*100.$$

For example, an architectural firm gave their junior architects the goal of submitting 30 villa concepts within a week. The firm’s team submitted 45 concepts, then the percentage goals met will be:

$$\left(\text{Actual Output}/\text{Goals}\right)*100=\left(45/30\right)*100=150\%$$

This implies that the firm’s junior team met 150% of the goal.

### 3.5 Application on existing office buildings

After analysing and reviewing the previous examples and various sources, it was determined that the three most influential features for improving building occupant productivity are natural light, natural ventilation, and thermal comfort. With this in mind, we have identified several features that can be easily implemented in existing buildings in Alexandria/Egypt (see Table 4).

**Table 4.** Conducted design criteria for application in existing office in Egypt: Author.

Design features	Additional detail
Operable Windows	• 75% of occupied areas have operable windows. • Minimum 4% area of net floor area. • Weather indicator light. • Manual shading controllable by occupants. • Automating shading to prevent glare. • Operable windows must be opened at least halfway, with the maximum height from the finished floor not exceeding 1.8 m and a minimum dimension of 0.3 m for the smallest opening. • Window operation control is at least 1.7 m above the finished floor. • Low opening windows for mild/warm weather. • High opening windows for wintry weather. • Easy to operate windows with minimum use of force and operates single handily, with enough space around it to operate.
Natural Light Design	• Within a 5 m distance between seatings and envelope glazing, VLT > 40%
Visual Lighting Design	• Minimum 320 lux at task surface.
Lighting Design for Circadian Rhythm	• The necessary light levels should be achieved from a height of 45 cm above the work plane. • Regular users can control their own lighting environment with manual controls found in the same area as each light zone. • Minimum one portable supplemental light is available.
Movement	• Height adjustable desk. • Height and depth adjustable chair. • Height adjustable devices. • Footrest. • Recessed toe space (min. 10 cm depth and height).
Thermal Comfort	• Availability of thermostat in the room. • Temperature sensors should be positioned at least 1m away from exterior walls, doors and windows, direct sunlight, air supply diffusers, mechanical fans, etc. • Radiant panels with minimum coverage of half the wall.
Mind	• Design elements that celebrate culture and space, integration of art, and human delight. • View of indoor plants and/or water and natural elements.
Sound	• Doors and walls sound isolation design.

## 4. Case study building

For the purpose of this paper, we focused on the “Cairo Petroleum Complex”, a medium-sized office building, as our case study. Access to the building and its blueprints have been granted to measure natural ventilation, daylighting, and thermal gain through simulations in DesignBuilder computer software for both base case and after criteria implementation.

### 4.1 Location and climate

**Figure 11.**
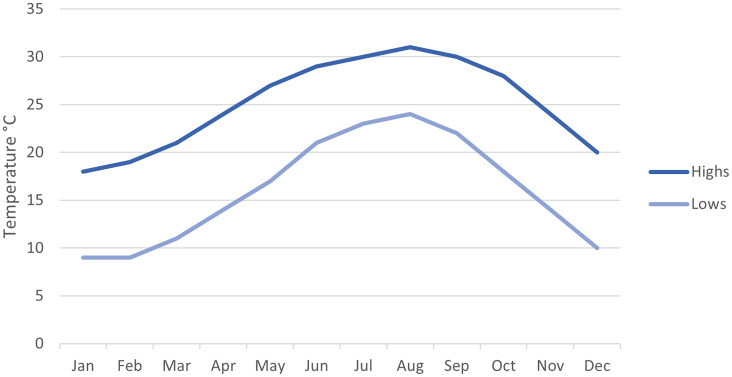
The monthly average high and low temperature in Alexandria / Egypt through the year 2023: NOAA.

The “Cairo Petroleum Complex” office building is located in Alexandria/Egypt, on the Alexandria-Cairo desert road, km 17.5. Alexandria’s climate is Mediterranean, with January and February the coolest months, featuring an average daily maximum temperature of 9 to 19 °C. July, August, and September are the hottest months, with an average daily maximum temperature of 30-31 °C (see Figure 11).

### 4.2 Brief

**Figure 12.**
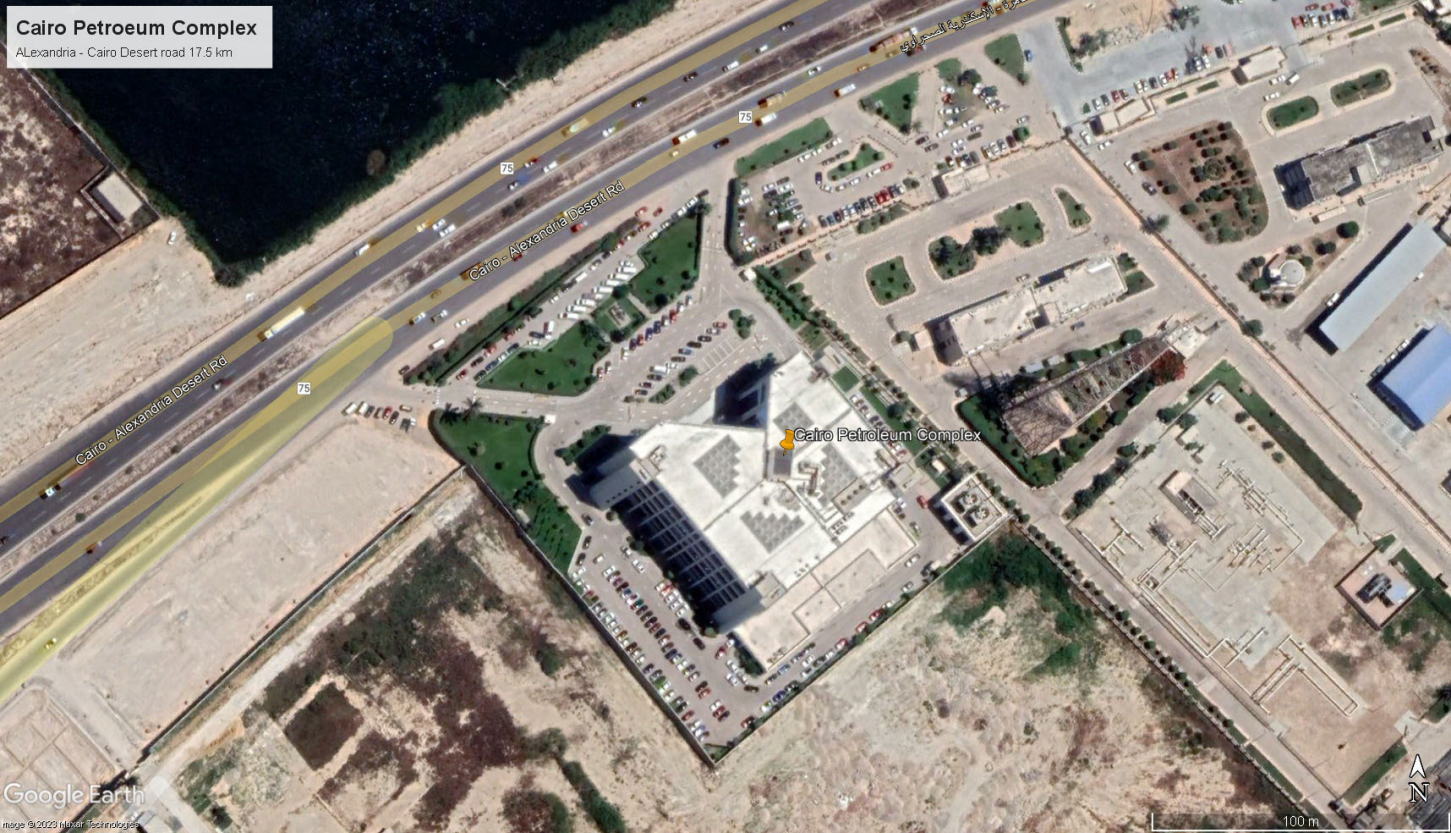
Bird’s-eye view of Cairo Petroleum Complex Building location: Google Earth.

“Cairo Petroleum Complex” Office Building was designed in 1995 and finished construction in 2000 (see Figure 12). It is composed of a total of 3370 m ^2^, with a rectangular shape measuring 60 m × 67 m and a height of 20 m, consisting of a ground floor and four typical floors. It is structured using reinforced concrete, with blue double-glazed curtain walls and aluminium frames, and no exterior shading. It accommodates 600 employees arriving at 8 a.m. until 4 p.m. for their eight-hour shifts on weekdays, aside from the control rooms, which operate 24/7. Presently, the building rents offices to eight different companies and a single bank. The offices are mechanically ventilated, with exterior walls constructed out of 20 cm brick, 2 cm exterior and interior plaster combination, and a total U-value of 1.5 W/m ^2^K. Meanwhile, the window-to-wall ratio across all elevations is 80%, except for the 90% that the North and Northwest double-glazed curtain walls have, with a U-value of 2.7 W/m ^2^K. Due to this, high mechanical loads are expected due to heat gain on the south and east-facing elevations. The northeast, northwest, southwest, and southeast elevations have 880 m ^2^, 198 m ^2^, 1072 m ^2^, and 960 m ^2^ of unshaded windows exposed to direct sunlight, totalling an overall exposed window area of 4010 m ^2^. The building contains four single-glazed skylights with a combined area of 240 m ^2^ and a U-value of 3.7 W/m ^2^K located above the primary court, with a flat, insulated roof with 3130 m ^2^ of reinforced concrete, interior paint, and insulation material with a U-value-of-0.5-W/m ^2^K (using the DesignBuilder computer software to identify the U-value within the building). ^58^

### 4.3 Building problems

**Figure 13.**
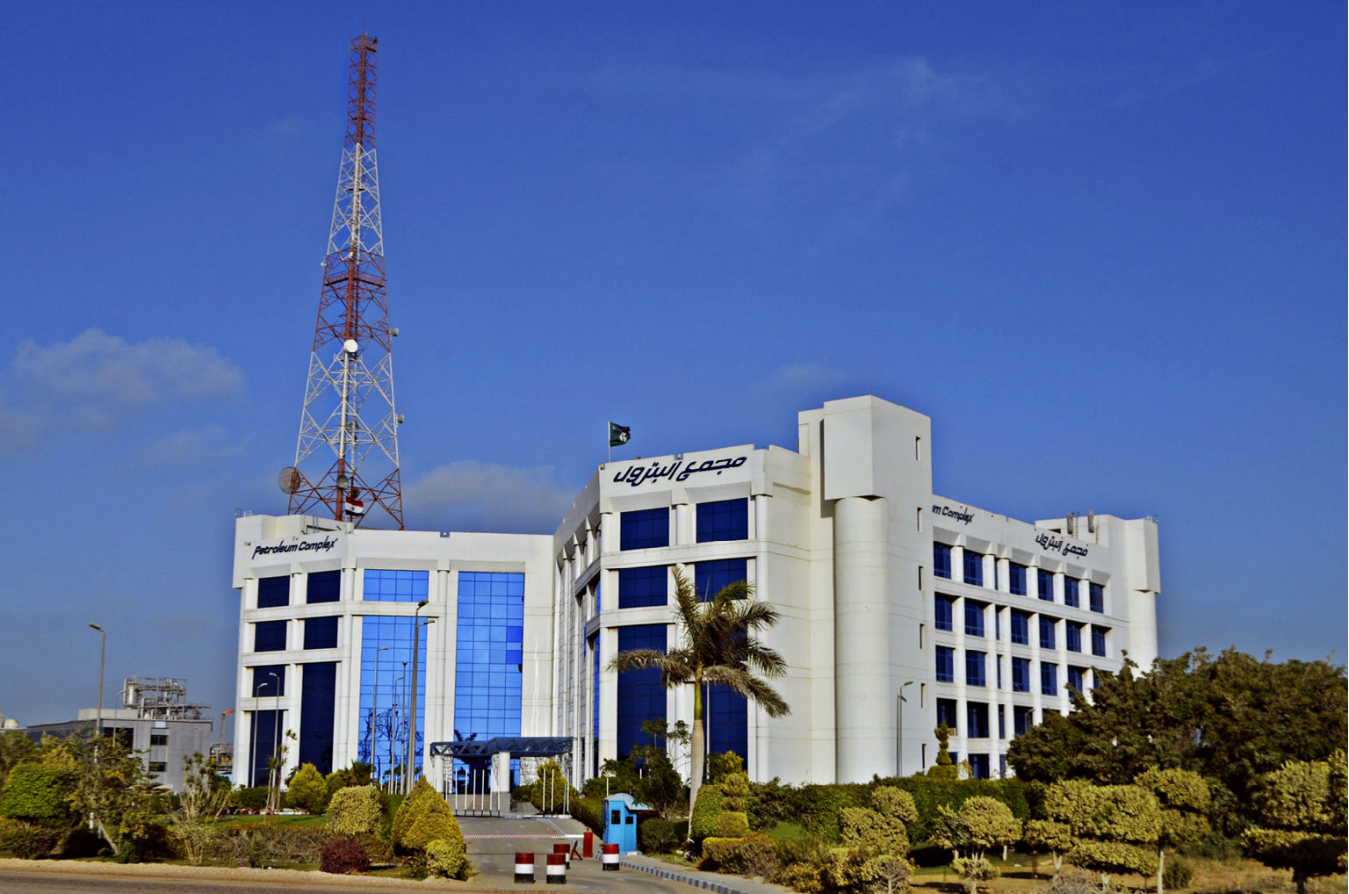
Cairo Petroleum Complex Building North-West main elevation showing the buildings Curtain wall façade: Wikipedia.

**Figure 14.**
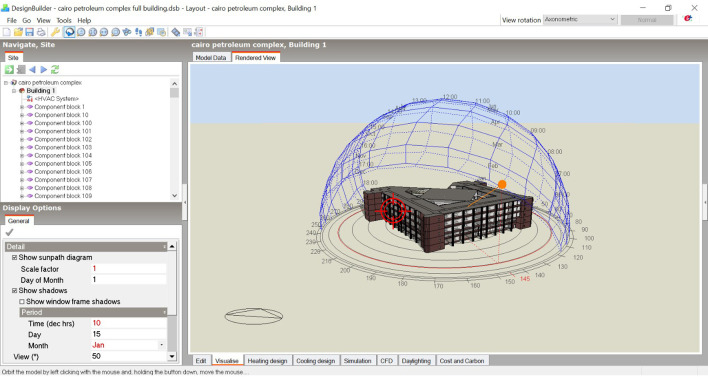
The three-dimensional model of the Cairo Petroleum Complex office building shows the solar path conducted by DesignBuilder on the selected office.

“Cairo Petroleum Complex” office building is a perfect example of modern architecture in Egypt, demonstrating an advanced mechanical ventilation system and a façade made mostly of curtain walls (see Figure 13). However, this combination has created various issues within the building due to thermal gain and glare caused by inadequate shading devices causing the West and South offices to be uncomfortable for employees to work in (see Figure 14 for solar path diagram and selected office). ^58^ Additionally, the building has inadequate natural ventilation as some facades have only 25% of the windows operable while others have none. Together these difficulties form a complex challenge requiring detailed consideration to ensure comfortable conditions with minimal energy consumption.

### 4.4 Selected office and possible solutions

**Figure 15.**
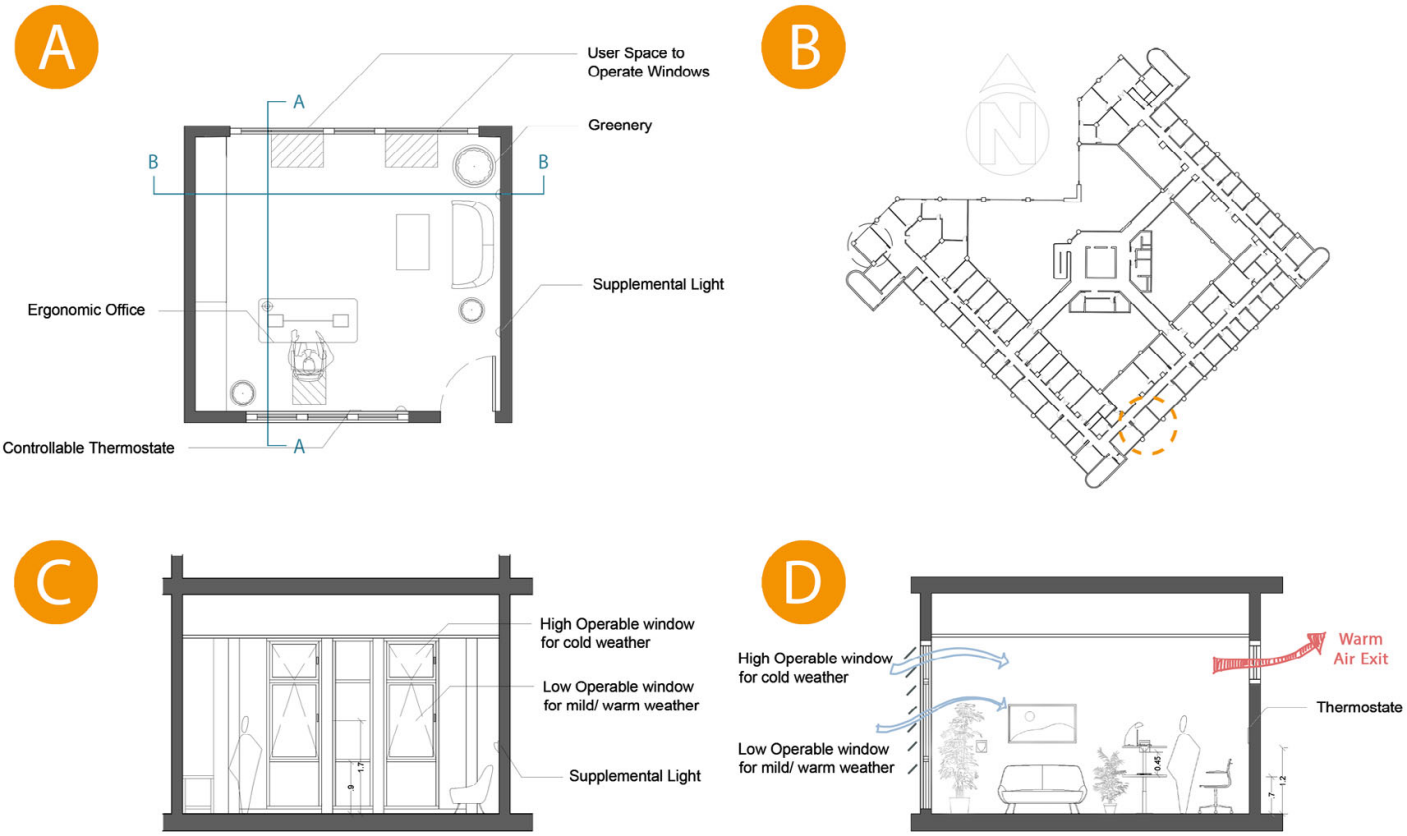
(A) Implemented criteria on case study office plan, (B) Building typical floor plan showing the chosen office, (C) Cross Section B-B of case study office room showing the recommended heights for window operation management at 1.7 m and openings with higher operable windows use for cold weather and lower operable window for mild/warm weather, (D) Cross section A-A showing predicted natural ventilation from different window heights and the supplemental light height at 0.45m above the work surface for the circadian solution. The photos (A), (B), (C), and (D) were acquired from the researcher’s work on the “Cairo Petroleum Complex” office building using Autodesk: AutoCAD software.

According to the employees’ statement, we chose a standard office room on the typical floor located on the South-East façade that was reported to suffer from thermal heat gain from the exterior façade and direct sunlight (see Figure 15). We apply the following modifications to the selected office for comparative simulation:

• **Curtain Wall:** Designing different height operable windows in the façade to allow natural ventilation, higher operable windows for winter, and lower operable windows for summer. Operable windows must be opened at least halfway, with the maximum height from the finished floor not exceeding 1.8 m and a minimum dimension of 0.3 m for the smallest opening. Window operation control is a minimum of 1.7 m above the finished floor.
• **Shading:** Application of automatic shading devices of curtain wall facades (louvres) to decrease direct sunlight entering the offices and eliminate glare.
• **Cross Ventilation:** Applying a high operable window opposite the curtain wall creates cross-ventilation.
• **Circadian Lighting Design:** The necessary light levels should be achieved from a height of 45 cm above the work plane.
• **Location of Furniture:** The distance between envelope glazing and seating area is within 5 m, VLT>40%.

### 4.5 Base case model validation

A modelling validation method was needed to conduct reliable results for modifying the base case model based on the comparison between observation and simulation. Through various modelling validation parameters, we selected “the Correlation Coefficient” parameter. The Pearson product-moment correlation coefficient, also known as “the correlation coefficient [ 2],” is a widely used statistical tool that measures the strength of the relationship between two variables. The correlation coefficient ranges from -1.0 to 1.0 and indicates the relative movements of the two variables being measured. A value of 1.0 indicates a perfect positive correlation, while a value of -1.0 shows a perfect negative correlation. A value of 0.0 indicates no relationship between the two variables. Pearson’s correlation coefficient is denoted by R, with R2 representing the squared value of the correlation coefficient. It is commonly used in various scientific fields. ^59^

The formula states:

$$r=\frac{n\left(\sum \mathrm{xy}\right)-\left(\sum x\right)\left(\sum y\right)}{\sqrt{\left[n\sum x^{2}-{\left(\sum x\right)}^{2}\right]}\left[n\sum y^{2}-{\left(\sum y\right)}^{2}\right]}$$

“ *r*” stands for “ *the correlation coefficient*”, “ *n*” stands for “ *number in the given dataset*”, “ *x*” stands for “ *first variable in the context*”, and “ *y*” stands for “second *variable*”.

**Figure 16.**
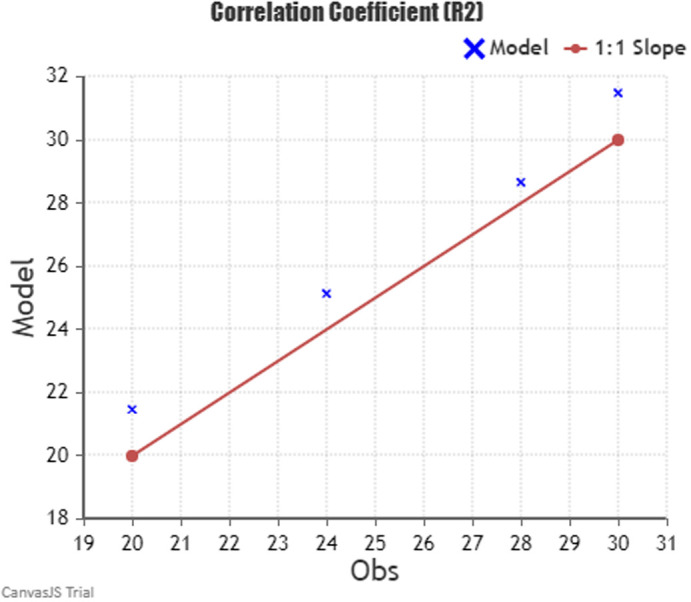
The Correlation Coefficient formula calculation chart.

By comparing the temperature data from the previous Figure 11 of Alexandria’s 2023 monthly weather data chart and the base case resulting data from DesignBuilder software, we conduct the following table:

By using the variables from Table 5 in the Correlation n Coefficient formula, we get the following results (see Figure 16):

$$\text{The correlation coefficient}\;\left({\text{R}}^{2}\right)\;\text{is}:0.993$$

$$\text{Pearson’ correlation coefficient}\;\left(R\right)\;\text{is}:0.997$$

**Table 5.** Required temperature data for September, October, November, and December gathered from Alexandria’s Weather chart and Base case temperature results for the Correlation Coefficient formula: Author.

Month	Observation	Simulation
September	30	31.46
October	28	28.66
November	24	25.14
December	20	21.47

The results show R ^2^ = 0.993, which is within the acceptable range of the correlation coefficient -1 and 1, thus indicating that the base case results are valid, reliable, and applicable.

### 4.6 Simulations of the base case and post-implementation

The following analysis diagrams compare the office base case and post-implementation results. Starting with the direct sunlight simulation results. ^58^

**Figure 17.**
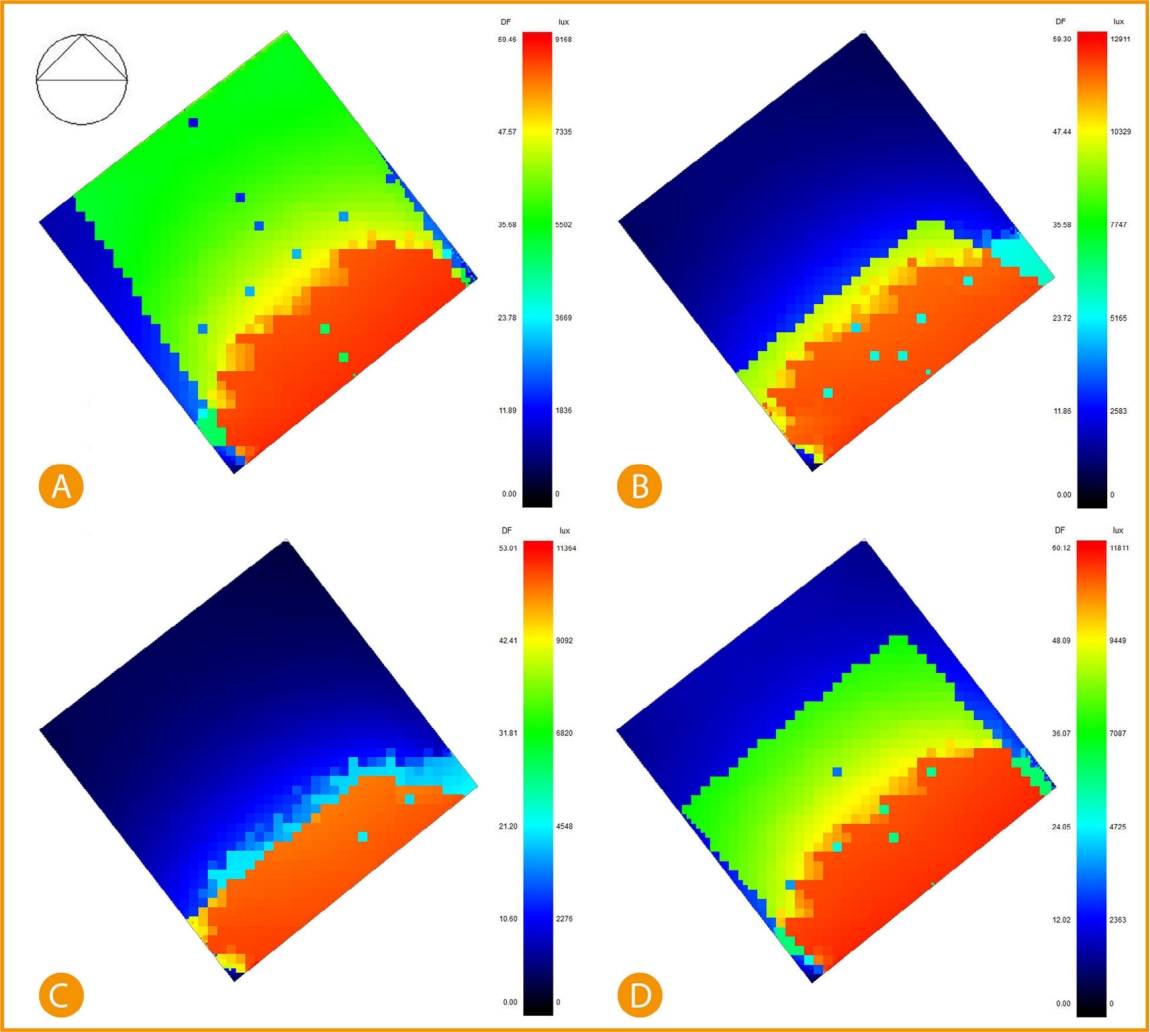
South-East office Base case direct sunlight simulation results, (A) January sunlight simulation, (B) April sunlight simulation, (C) July sunlight simulation, and (D) October sunlight simulation. The photos (A), (B), (C), and (D) were acquired from the researcher’s work on the “Cairo Petroleum Complex” office building using DesignBuilder software.

Using DesignBuilder computer software to simulate the base case of direct sunlight entering the office, we find that January and October are the highest recorded sunlight rate entering the building. January recorded direct sunlight of 7335-9168 lux reaching around 28% office space, while 60% is Indirect daylight of about 5502 lux, and 12% is daylight 0-1836 lux. April records 27.1% direct sunlight of around 12911 lux, 9.1% 10329 lux, 1.6% 5165 lux and the rest 62.1% between 0-2583 lux. July records 21.7% direct sunlight of 11364 lux, 7.9% records 9092 lux and the rest, 70.4%, records between 0-4548 lux. Lastly, October records 30% direct sunlight of 9449-11811 lux, 35% indirect sunlight of around 7087 lux, and the other 35% records 0-2363 lux (see Figure 17).

**Figure 18.**
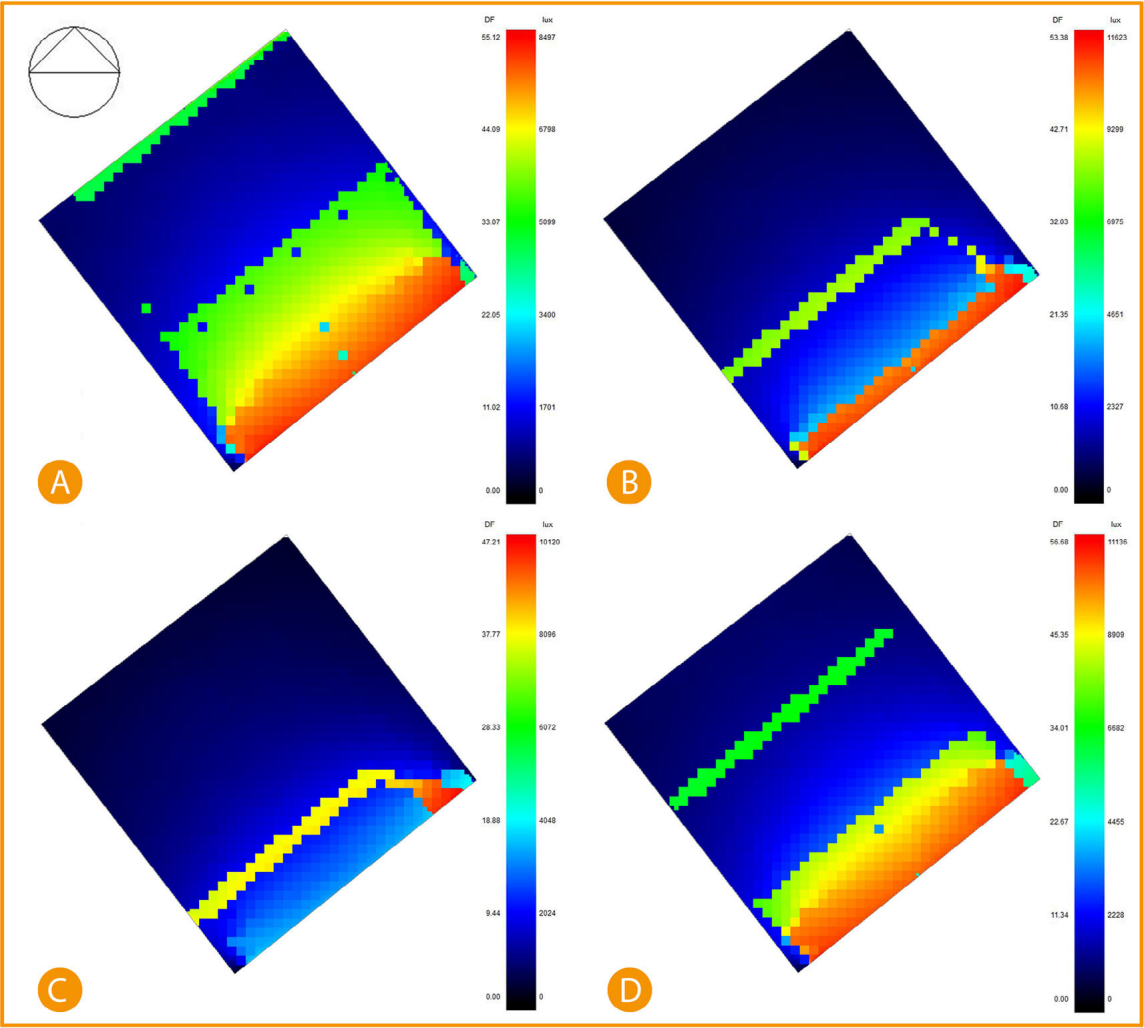
South-East office Post-implementation direct sunlight simulation results, (A) January sunlight simulation, (B) April sunlight simulation, (C) July sunlight simulation, and (D) October sunlight simulation. The photos (A), (B), (C), and (D) were acquired from the researcher’s work on the “Cairo Petroleum Complex” office building using DesignBuilder software.

While direct sunlight simulation post-implementation shows significant improvement and decrease in the area exposed to direct sunlight, the results were simulated after adding an automatic shading device (louvres) on the southeast façade. The results show January recorded 19% of exposed area in the range between 6798 lux to 8497 lux of direct sunlight, 27% records around 5099 lux, and the 54% remaining area are recorded at between 0-3400 lux, April records only 5.2% of the area is direct sunlight of 11623 lux, 5.9% records 6975 lux, and the rest 88.9% records between 2327-4651 lux. July records only 7.4% area of direct sunlight at 10120 lux, and the rest of 92.6% area is recorded between 2024-4048 lux. Lastly, October recorded 20% of direct sunlight area at 8909-11136 lux, 9.34% records 6682 lux, and 70.26% records between 2228-4455 lux (see Figure 18). ^58^

Comparing the base case and post-implementation results, we see that in January, there was a decrease in the total lux by **7.3%** and a decrease in the direct sunlight exposure area by **9%.** In April, the total lux decreased by around **10%,** and the direct sunlight exposed area decreased by **21.9%.** In July, total lux decreased by **10.9%,** and the direct sunlight exposed area decreased by **14.3%.** Lastly October, total lux decreased by **5.7%,** and the direct sunlight exposed area decreased by **10%.**

**Figure 19.**
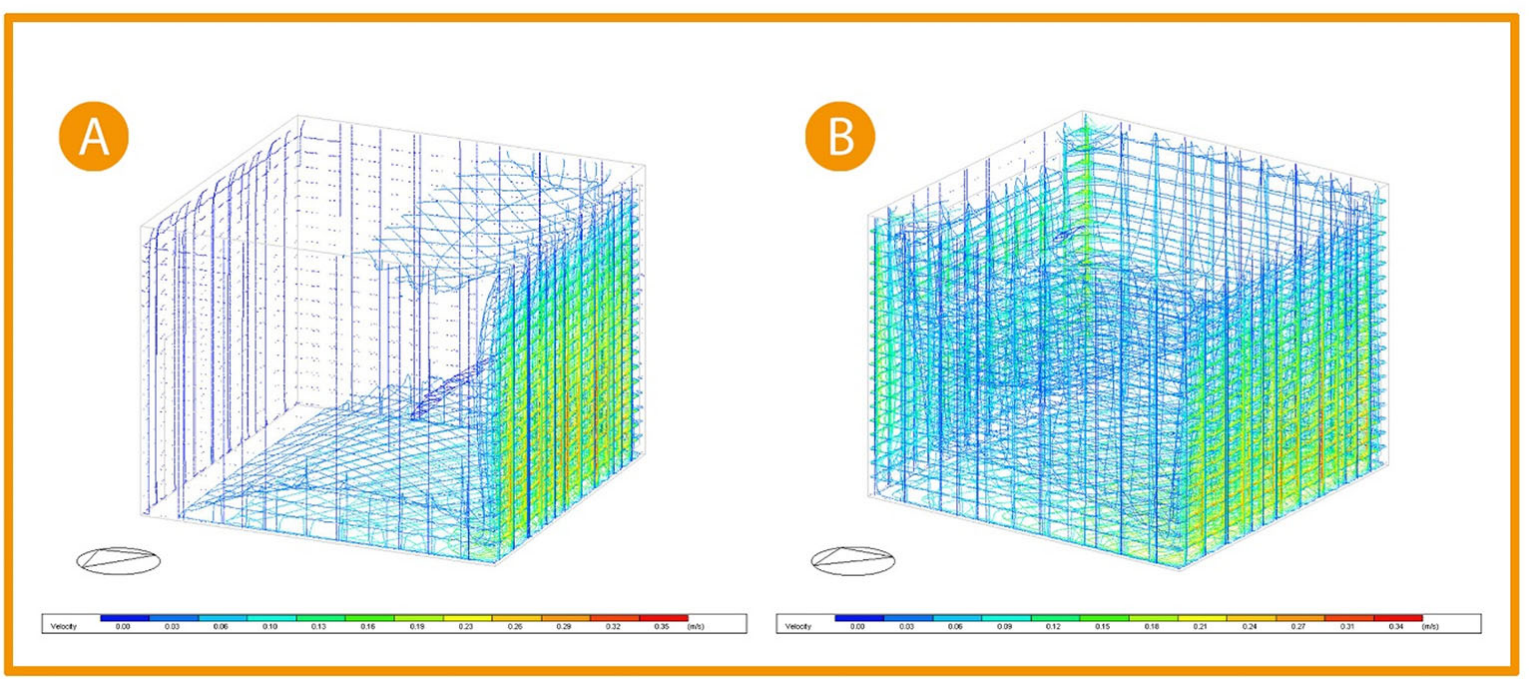
Three-Dimensional Air flow simulation comparison chart (A) Base case, (B) Post-implementation. The photos (A), and (B) were acquired from the researcher’s work on the “Cairo Petroleum Complex” office building using DesignBuilder software.

It was conducted through the previous studies that applying around 25% operable windows to the office façade and placing a high window opposite to the curtain wall windows creating cross ventilation, indicated an increase in air flow in the space (see Figure 19). ^7^

Picture (A) Figure 19 shows the base case 3D simulation for airflow; the simulation indicates that the airflow is minor, and its velocity ranges between 0.01-0.10 m/s closer to the floor, increasing to 0.19 m/s near the curtain wall. While in picture (B), after adding windows to the façade and opposing it to create cross ventilation, the airflow increased to cover all the office area with a slight increase in the velocity ranging between 0.06-0.12 m/s all around the office while not affecting the comfort of users. The air velocity at the building envelope remains the same. Thus, making a **20% improvement** in air flow. ^58^

**Figure 20.**
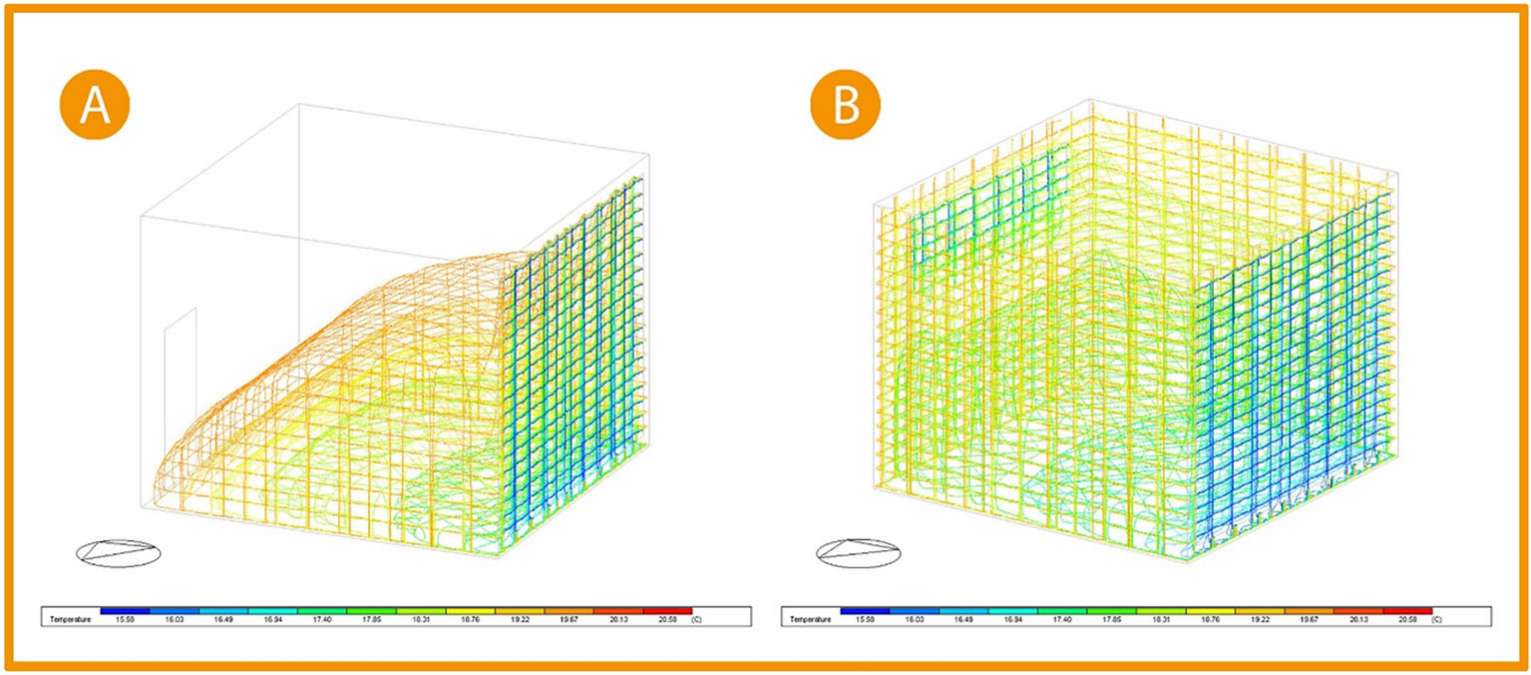
Three-Dimensional thermal distribution comparison chart (A) Base case, (B) Post-implementation. The photos (A) and (B) were acquired from the researcher’s work on the “Cairo Petroleum Complex” office building using DesignBuilder software.

As for the thermal distribution analysis, providing an automatic shading system to the building’s façade along with windows that increased airflow indicated the wider distribution of cooler temperature, leading to thermal comfort. Figure 20, picture (A) shows the three-dimensional thermal distribution in the base case office to cover less area, with the average temperature for user area ranging between 17.85 °C and 19.67 °C (colour indication: orange, yellow, light green, and green). While after implementation in the picture (B), it shows the 3D temperature distribution after modification increases to cover the whole office area leading to an increase in the temperature affected by thermal gain. As the colour indication shows, the orange zone colour decreased (indicating a temperature zone of 19.67 °C), and the usable zone temperature records range between 16.49 °C and 18.31 °C (colour indication: cyan, green, and light green). Thus, the average temperature decreases by 1.37 °C, which is a **7% improvement.** ^58^

**Figure 21.**
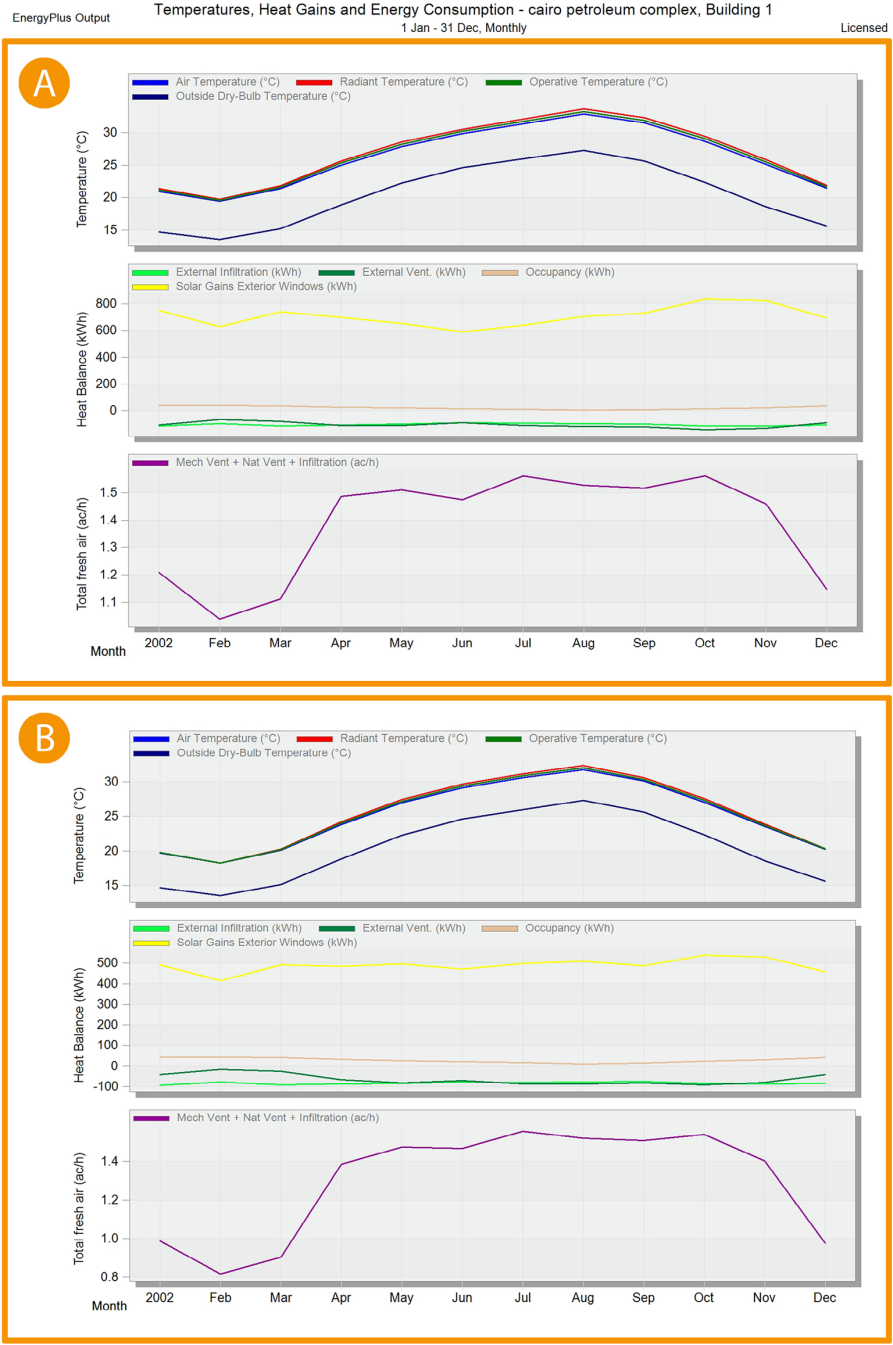
EnergyPlus monthly simulation results for Temperatures, heat gains and energy consumption of the selected office from 1 Jan- 31 Dec (A) Base case, (B) Post-criteria implementation. Showing the increase in natural ventilation and decrease in solar glare and thermal gain. The photos (A) and (B) were acquired from the researcher’s work on the “Cairo Petroleum Complex” office building using the computer software “DesignBuilder” to simulate the changes on the space.

**Figure 22.**
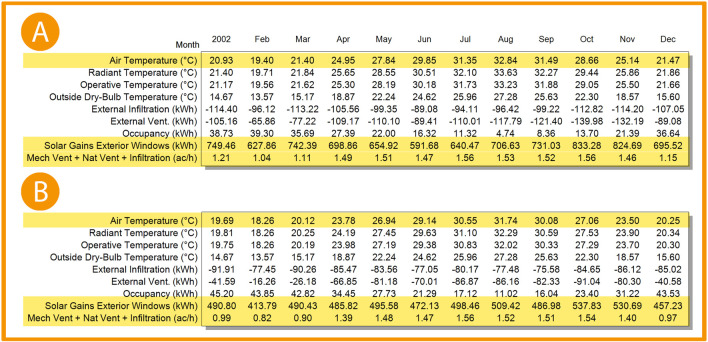
EnergyPlus monthly simulation results for Temperatures, heat gains, and energy consumption of the selected office from 1 Jan- 31 Dec (A) Base case, (B) Post-criteria implementation. Showing increased natural ventilation and decreased solar glare and thermal gain. The photos (A) and (B) were acquired from the researcher’s work on the “Cairo Petroleum Complex” office building using the computer software “DesignBuilder” to simulate the changes in the space.

**Figure 23.**
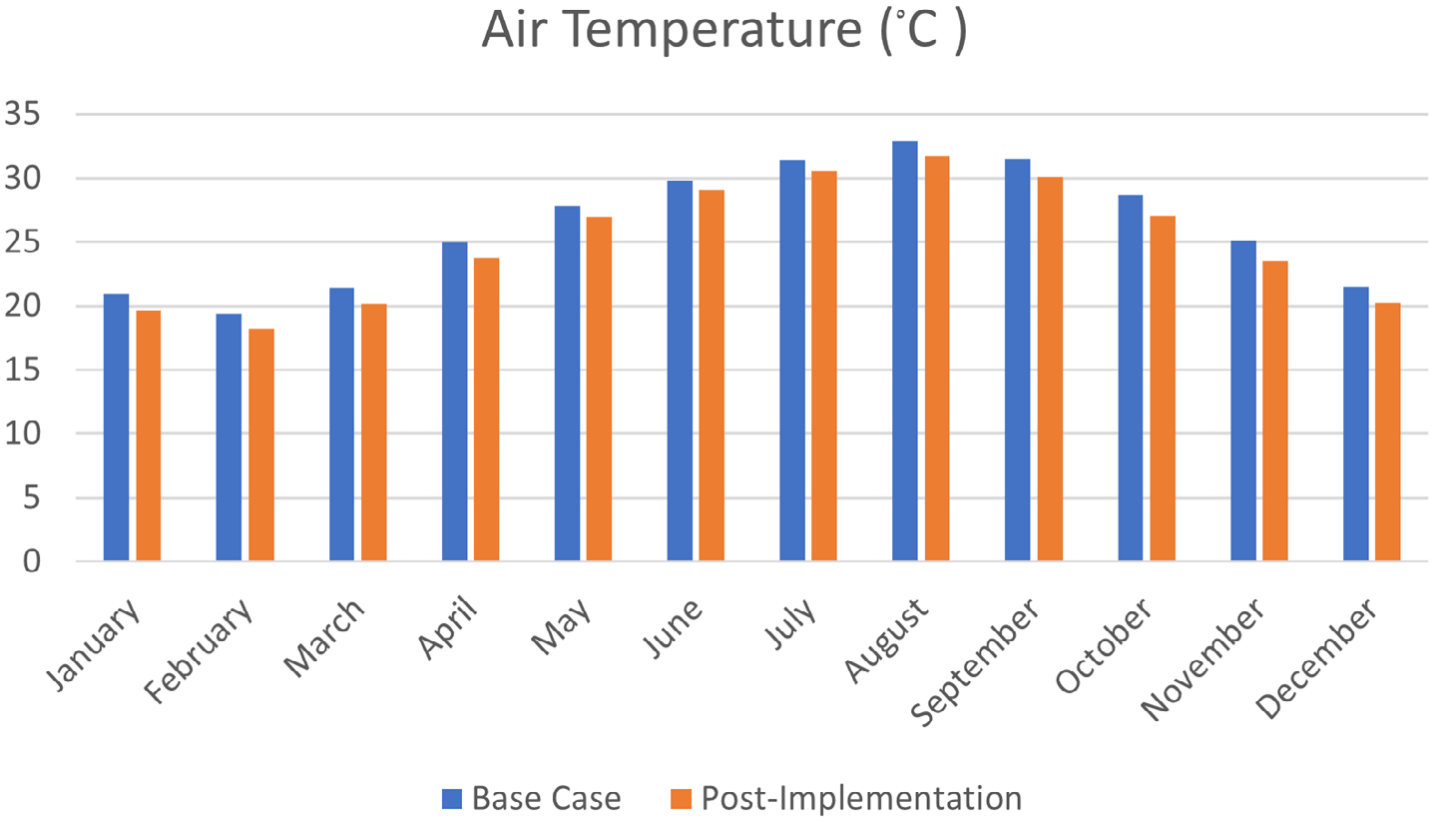
Comparison bar chart between base case and post-implementation Air temperature monthly simulations results.

As shown in Figure 21, Figure 22, and Figure 23, in January and February, air temperature decreased by 5.9%, 6% in March, 4.7% in April, 3.2% in May, 2.4% in June, 2.6% in July, 3.3% in August, 4.5% in September, 5.6% in October, 6.5% in November, and 5.7% in December. ^58^

**Figure 24.**
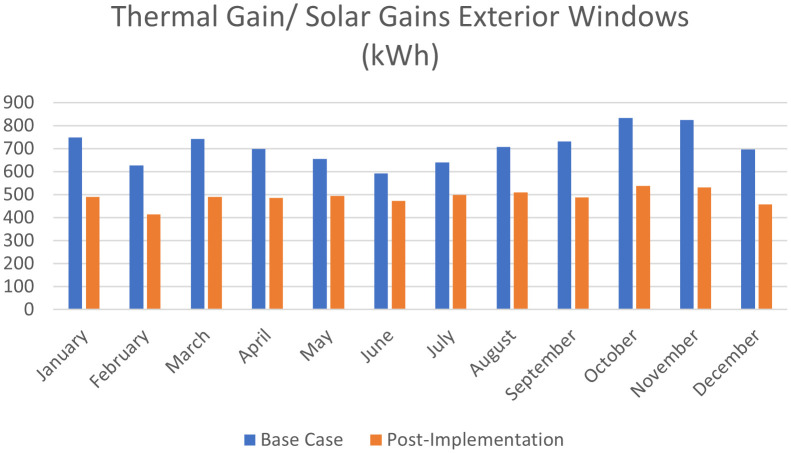
Comparison bar chart between base case and post-implementation monthly Thermal gain/Solar gains exterior windows simulations results.

For the thermal gain/solar gains exterior windows comparison simulation results, January records 34.5% decrease in thermal gain, 34.1% in February, 33.9% in March, 30.5% in April, 24.3% in May, 20.2% in June, 22.1% in July, 27.9% in August, 33.4% in September, 35.5% in October, 35.6% in November, and 34.3% in December (see Figure 21, Figure 22, and Figure 24). ^7^^,^^58^

**Figure 25.**
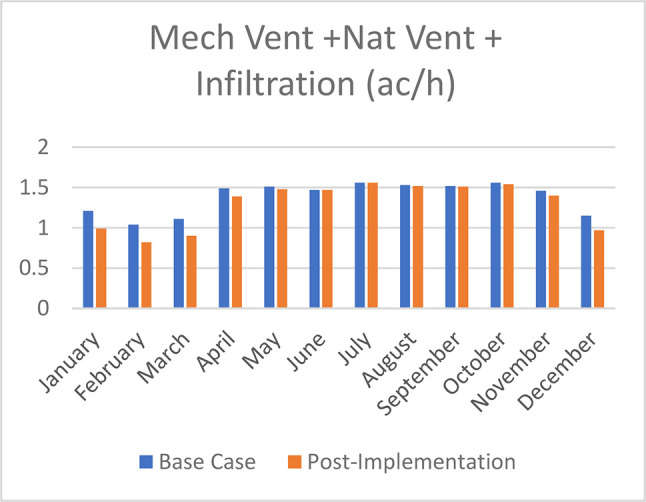
Comparison bar chart between base case and post-implementation of mechanical ventilation, Natural Ventilation, and Infiltration monthly simulations results.

Mechanical Ventilation, Natural Ventilation, and infiltration decreased by 18% in January, 21.2% in February, 19% in March, 6.7% in April, 2% in May, no effect in June and July, 0.65% in August and September, 1.3% in October, 4.1% in November, and 15.7% in December (see Figure 21, Figure 22, and Figure 25).

In addition to providing supplemental light 45 cm above the worksurface to influence employees’ circadian rhythm. According to the future workplace wellness study and other research, it was assured that by achieving thermal comfort, natural ventilation and natural light, an increase in productivity is inevitable. ^10^^,^^58^

## 5. Conclusion and recommendations

### 5.1 Research conclusion

Studying WELL Building standards and other green building rating systems and recognising the success of popular existing office buildings shows great promise for improving occupants’ well-being and productivity. This is especially crucial after the pandemic, as business districts in Egypt must implement these structures to ensure a safe yet productive environment. Regarding present and future circumstances, this kind of research is increasingly vital for establishing better practices in the workplace.

This paper studies successful design criteria for existing office buildings to reach a conducted design criteria for healthy office design to enhance productivity in the workspace.

By studying successful office designs like Googleplex building and Amazon spheres, we conducted the first design criteria indicating that natural light, thermal comfort, natural ventilation, nature, and mental health design are key for a successful and productive environment.

Next, by studying and analysing WELL Building Rating system concepts, we conducted the second design criteria, which explains how to achieve adequate levels of natural light, natural ventilation, and thermal comfort in addition to sound and designing for the mind to get employees to thrive in their work area.

Lastly, by applying our findings to the selected case study building, we see the building simulation results calculated using DesignBuilder computer software.

### 5.2 Case study findings and conclusion

The simulations show that by applying the design implementations on the selected case study, the thermal heat gain was reduced after using automatic shading devices by an average of 20.2%-35.6% throughout the year, Airflow increased by 20% after adding 25% user-friendly designed operable windows to the building’s façade and opposite operable windows for cross ventilation. Lastly, adding double-glazed glass for the curtain wall and the automatic shading device enhanced the illuminance distribution, temperature distribution, and air temperature. Direct sunlight area decreased by 9% in January (Winter), 21.9% in April (Spring), 14.3% in July (Summer), and 10% in October (Autumn). Air temperature decreased by a minimum of 2.4% in June and a maximum of 6.5% in November—temperature distribution enhanced by an average of 7%. ^58^

### 5.3 Research recommendations

This paper concluded the top three effective design features from the literature review, example analysis and applying and testing their effect on a selected case study in a medium sized-office building; there is a possibility that the conducted design features can be used on an entire existing office building in Egypt. This paper proposes some criteria for increasing productivity in existing office buildings scale as well as individual workplaces, including:

• Providing adequate natural ventilation and air quality through windows and cross ventilation while considering easy window operation management.
• Providing adequate natural light while considering circadian rhythm design elements.
• Use automatic shading devices (such as louvres, etc.) to function in all seasons.
• Seeking thermal comfort, proper temperature distribution and furniture placement.
• Connecting to nature boost productivity and health.

It is recommended that productivity measuring method is used to rate the existing buildings’ employees’ productivity. These productivity rates could be enhanced by using the conducted criteria. We recommend that architects and construction firms consider this design criteria in the designing phase of buildings to enhance the performance of occupants through encouraging healthy building design. Additionally, the legislative authorities and the government should be required to include conditions that support the health and well-being of occupants in the new construction law for those requesting construction permits.

## Data Availability

Mendeley data: Well-being as a tool to improve productivity in existing office space. Doi: https://doi.org/10.17632/d5g9vwt28s.1. ^58^ This project contains the following underlying data:
- Adobe Photoshop [Cairo Petroleum Complex architecture plans and cross-sections presentations] - Autodesk AutoCAD [An architectural detailed plans and cross-section for the Cairo Petroleum Complex office building] - DesignBuilder [A three-dimensional simulation of the Cairo Petroleum Complex case study building for the base case and the post-implementation of the conducted criteria] Data are available under the terms of the Creative Commons Attribution 4.0 International license (CC-BY 4.0).

## Footnotes

^1^
Sedentary behavior is distinct from physical inactivity and is characterized as very low-intensity, low-effort activities, such as sitting.

^2^
The correlation coefficient was developed by Karl Pearson in the 1880s based on an idea from Francis Galton, with the mathematical formula established by Auguste Bravais in 1844.

## References

[1] TED: The Economics Daily:2022 [cited 2023 25 March]. Reference Source

[2] DONOFF E : *The Energy Crises of the 70s.* ARCHITECT: THE JOURNAL OF THE AMERICAN INSTITUTE OF ARCHITECTS;2016.

[3] Arnett A : *Why is the WELL Building Standard Worth Striving For?* FER: Foodservice Equipment Reports;2021.

[4] Quintana K : *WELL @ Work.* 2017.

[5] EPA: *Why Indoor Air Quality is Important to schools.* [cited 2023 12 March]. Reference Source

[6] *The total exposure assessment methodology (TEAM) study: Summary and analysis. EPA/600/6-7/002a.* Washington, DC.: U.S. Environmental Protection Agency;1987.

[7] *Indoor Air Quality: What are the trends in indoor air quality and their effects on human health?* The United States Environmental Protection Agency (EPA);2021.

[8] Vaughn E : *Redesigning The Office For The Next 100-Year Flu (Yes, It’s Coming). npr.* 2020.

[9] Kaysen R : *The Post-Pandemic Office.* Architectural Record;2022.

[10] Future Workplace, V: *Future Workplace Wellness Study.* research study. 2019 [cited 2023 20 March]. Reference Source

[11] Faragg N : Sick building syndrome and office space design in Cairo, Egypt. *Sage Journals.* 2021;31(2):568–577. 10.1177/1420326X211016507

[12] Ritchie D : *10 Office Design Tips That Will Boost Employee Productivity.* Calendar;2019.

[13] *Ways to Design a Productive Office Space.* Pinnacle Interiors;2022.

[14] Post J : How to Create a Workspace That Improves Productivity. *Business News Daily.* 2022.

[15] Alker J : Health, wellbeing & productivity in offices: The next chapter for green building. 2014.

[16] Neufert E : *Neufert Architect’s Data.* fourth edition. Wiley-Blackwell;2012.

[17] Nigel Oseland RB : Michael Hadcocks. *The Future of UK Office Densities.* British Council for offices (BCO);2022.

[18] McMahon C : HOW MUCH OFFICE SPACE DO WE NEED PER EMPLOYEE?. 2023 Jan. 12 [cited 2023 03/02]. Reference Source

[19] Petrusky M : *How Much Office Space Do We Need Per Employee?* IOffice by Eptura;2020.

[20] OBeirne S : BCO recommends allocating more space-per-person for the post-pandemic office. *FMJ: Facilities Management Journal.* 2022.

[21] *Office Space Planning Guidelines For Returning To Work and a Better Occupant Experience*, in *Kontakt.io.* Aneta Ciurkot;2022.

[22] *How Will Covid-19 Change Demand For Office Space?* WSP.

[23] (WorldGBC), W.G.B.C: Principle 1: Protect and Improve Health. [cited 2023 12 Feb]. Reference Source

[24] Council, W.G.B: *Health, Wellbeing & Productivity in Offices.* World Green Building Council (WorldGBC): World Green Building Council;2014.

[25] Haiken: *What are the 5 key elements to a good office design?* Haiken;2021.

[26] L’Estrange S : A Functional Post-Pandemic Office That Inspires. *Workdesign Magazine.* 2021.

[27] Brownell B : *rethinking office design trends in a post-covid world.* Architect magazine;2020.

[28] *Googleplex*, in *wikipedia.* wikipedia wikipedia.

[29] Tudorache A : *The ultimate level of employee satisfaction: Working at the Googleplex.*, in *Performance Magazine.* The KPI Institute;2013.

[30] Wikipedia: Amazon Spheres. *Wikipedia. Wikipedia: Wikipedia.*

[31] WenMing Ye ME : Inference at the Edge: A Case Study at the Amazon Spheres. *solaripedia.* 2018.

[32] Smith A Pitt M : Sustainable workplaces: improving staff health and well-being using plants. *J. Corp. Real Estate.* 2009;11(1):52–63.

[33] Wargocki P Wyon DP Fanger PO : Productivity is affected by the air quality in offices. *Proceedings of Healthy Buildings.* 2000.

[34] Sop Shin W : The influence of forest view through a window on job satisfaction and job stress. *J.S.j.o.f.r.* 2007;22(3):248–253. 10.1080/02827580701262733

[35] Grahn P Stigsdotter UKJL : The relation between perceived sensory dimensions of urban green space and stress restoration. *Landsc. Urban Plan.* 2010;94(3-4):264–275. 10.1016/j.landurbplan.2009.10.012

[36] Lottrup L : Associations between use, activities and characteristics of the outdoor environment at workplaces. *Urban For. Urban Green.* 2012;11(2):159–168.

[37] Lottrup L : The workplace window view: a determinant of office workers’ work ability and job satisfaction. *Landsc. Res.* 2015;40(1):57–75.

[38] Gilles Vandewalle PM Dijk DJ : Light as a modulator of cognitive brain function. *Trends Cogn. Sci.* 2009;3(10):429–438. 10.1016/j.tics.2009.07.004 19748817

[39] *The WELL Building Standard (WELL).* WELL v2. 2022 [cited 2020. Reference Source

[40] Lee KK : Adverse health effects associated with household air pollution: a systematic review, meta-analysis, and burden estimation study. *Lancet Glob. Health.* 2020;8(11):e1427–e1434. 33069303 10.1016/S2214-109X(20)30343-0 PMC7564377

[41] Beemer CJ : A brief review on the mental health for select elements of the built environment. *Indoor Built. Environ.* 2021;30(2):152–165.

[42] Sallis JF Bull F Guthold R : Progress in physical activity over the Olympic quadrennium. *Lancet.* 2016;388(10051):1325–1336. 10.1016/S0140-6736(16)30581-5 27475270

[43] Bauman A : The descriptive epidemiology of sitting: a 20-country comparison using the International Physical Activity Questionnaire (IPAQ). *Am. J. Prev. Med.* 2011;41(2):228–235. 21767731 10.1016/j.amepre.2011.05.003

[44] Owen N : Sedentary behaviour and health: mapping environmental and social contexts to underpin chronic disease prevention. *Br. J. Sports Med.* 2014;48(3):174–177. 24415410 10.1136/bjsports-2013-093107

[45] Young DR : Sedentary behavior and cardiovascular morbidity and mortality: a science advisory from the American Heart Association. *AHA Journals: Circulation.* 2016;134(13):e262–e279. 10.1161/CIR.0000000000000440 27528691

[46] Chau JY : Daily sitting time and all-cause mortality: a meta-analysis. *PLoS One.* 2013;8(11):e80000. 24236168 10.1371/journal.pone.0080000 PMC3827429

[47] Patterson R : Sedentary behaviour and risk of all-cause, cardiovascular and cancer mortality, and incident type 2 diabetes: a systematic review and dose response meta-analysis. *Eur. J. Epidemiol.* 2018;33:811–829. 29589226 10.1007/s10654-018-0380-1 PMC6133005

[48] Biswas A : Sedentary time and its association with risk for disease incidence, mortality, and hospitalization in adults: a systematic review and meta-analysis. *Ann. Intern. Med.* 2015;162(2):123–132. 25599350 10.7326/M14-1651

[49] Nicol JF Humphreys MAJE : Adaptive thermal comfort and sustainable thermal standards for buildings. *Buildings.* 2002;34(6):563–572. 10.1016/S0378-7788(02)00006-3

[50] Mental health: strengthening our response. *Fact sheets.* 2022 [cited 2023 15 Feb]. Reference Source

[51] Vigo D Thornicroft G Atun R : Estimating the true global burden of mental illness. *Lancet Psychiatry.* 2016;3(2):171–178. 10.1016/S2215-0366(15)00505-2 26851330

[52] Steel Z : The global prevalence of common mental disorders: a systematic review and meta-analysis 1980–2013. *IEA: Int. J. Epidemiol.* 2014;43(2):476–493. 10.1093/ije/dyu038 PMC3997379 24648481

[53] Mental Health and Substance Use: Mental health at work.[15/2/2023]. Reference Source

[54] Meister JC : The #1 Office Perk? Natural Light. *Harvard Business Review.* 2018.

[55] Schreyer P Pilat D : Measuring productivity. *J. EconPapers.* 2001;2001(2):127–170. 10.1787/eco_studies-v2001-art13-en

[56] Statistics, U.S.B.o.L: HOW IS PRODUCTIVITY MEASURED? *Labour Input.* [cited 2023 9 March]. Reference Source

[57] Miller K : 7 best ways to measure productivity of employees. *AboutLeaders.* 2023.

[58] Hamadah M : Well-being as a tool to improve productivity in existing office space. *Mendeley Data.* 2023. 10.17632/d5g9vwt28s.1 PMC10797229 38249134

[59] Software, A.a.M: *AgriMetSoft.* R2 (correlation coefficient). 2019 [cited 2023 6 april]. Online Calculators. Reference Source

