# Well-being as a tool to improve productivity in existing office space: Case study in Alexandria, Egypt

**DOI:** 10.12688/f1000research.133199.2

**Published:** 2023-12-14

**Authors:** Miral Hamadah, Ahmed ElSeragy, Sally ElDeeb

**Affiliations:** 1 Department of Architectural Engineering & Environmental Design, Arab Academy for Science Technology and Maritime Transport, Alexandria, Alexandria Governorate, 21500, Egypt; 2 School of Engineering, University of Lincoln, Lincoln, England, 19352, UK

**Keywords:** WELL, Rating System, well-being framework, Sick Building Syndrome, post-pandemic office buildings design standards, designing for occupants, Enhancing office productivity, Productivity design standards, Healthy Buildings Design

## Abstract

**Background:**

The green building industry has significantly impacted the construction market, providing various sustainable solutions for the community. However, conventional green building standards have yet to adequately address occupant health and well-being, leading to challenges with performance. This has caused many businesses to take note of the latest report from the Bureau of Labour Statistics, which indicated that productivity in the US has dropped by the sharpest level since the 1940s. ^1^ Addressing these issues, organisations like International WELL Building Institute (IWBI) developed WELL Building Rating System (WELL), prioritising occupant health and well-being as critical components for improving performance and avoiding potential vulnerabilities brought about by sickness or pandemics. For this reason, this study will explore how to improve employee productivity within office buildings by bettering their overall health and well-being.

**Methods:**

A comprehensive data collection approach was employed in this paper, involving the analysis of office form evolution, and the evaluation of productivity attributes in office spaces. Resulting in identifying the top design-oriented features impacting employee productivity. Data was gathered from traditional office designs, trending successful office buildings, and the WELL Building Rating System to understand the concept of healthy building design.

**Results:**

Showing thermal comfort, ventilation, and natural daylight significantly influence employees’ productivity. Implementing conducted design features from WELL achieved an average of 20.2%-35.6% decrease in thermal gain throughout the year, a 20% increase in airflow, an average 2.4%-6.5% decrease in Air temperature, enhanced temperature distribution by 7%, and direct sunlight minimum reduction by 9% in Winter and maximum 21.9% in Spring.

**Conclusion:**

New design features in trending successful office buildings positively impact employee productivity. Particularly the outlined features by WELL Building Rating System led to identifying the most influential factors affecting occupant productivity. The results of this study informed recommendations for enhancing productivity in existing office buildings in Alexandria, Egypt.

## 1. Introduction

The operation and construction of current and new buildings negatively impacted the environment for countless years. ^2^ In addition to the 1970s energy crisis, builders and building owners were encouraged to turn to green building rating systems to assist them in designing more environmentally conscious buildings and communities.

Despite the emergence of various renowned green building standards, a predominant focus has been observed on attaining sustainability in buildings to enhance environmental quality. The issue of occupants’ health and wellness, which can significantly impact societal health and productivity—particularly within the business sector due to its influence on employee performance—has been insufficiently addressed. ^1^^,^^3^

Thus, another movement started in 2014 that included human health and well-being and how buildings affect occupants as a new factor to the green movement equation. ^3^ That is when International WELL Building Institute (IWBI) created the WELL Building Standard, the first system of its kind that tracks, measures, and certifies how building features affect the people inside. ^4^

Studies indicate that 90% of people’s time is spent in enclosed spaces, which impacts their health. ^5^^,^^6^ Potential exposure to multiple pollutants, including air pollution, substandard lighting and ventilation, indoor toxic materials, uncomfortable temperatures, and overlooked mental health and social environment well-being, may occur during this period. ^7^ These conditions, which can be up to five times higher than typical outdoor concentrations, are implicated in adverse short-term and long-term health and well-being outcomes. ^8^^,^^9^ These circumstances are held accountable for the diminished productivity and increased health vulnerability of employees in the workspace, especially during pandemics. The recent emergence of the COVID-19 pandemic has underscored the urgent need for operational changes. ^10^^,^^11^

While recent research has found that Air Quality, Thermal Comfort, and Natural Light are the three highest factors affecting office productivity, this is a significant finding as it shows that these factors directly impact employee performance. ^12^^–^^16^ The findings suggest that companies should prioritise improving air quality, providing adequate thermal comfort, and increasing natural light in their office spaces to maximise staff productivity. Additionally, this data can help inform businesses about office design and layout decisions to create an environment conducive to employees’ well-being and success. ^17^

### 1.1 Research problem

Office buildings globally suffered a severe drop in productivity rates of their employees caused by several factors, including the most recent hit of the COVID-19 pandemic, accelerating the need to change how we live and work. ^18^ This has prompted architects and business owners worldwide to adjust office building designs to minimize the risk of infection and improve productivity. However, typical office buildings in Egypt reports several causes for employees’ discomfort caused by the buildings architectural design features (as full curtain wall façades with inadequate ventilation, lack of operable windows, natural light, and thermal comfort) leading to vulnerability to infection, low motivation levels, and poor productivity rates. ^19^

### 1.2 Research aim

This paper aims to direct the entire design team (architects, construction firms, building services engineers, and the legislative authorities) in Egypt to include the well-being of occupants as an essential key factor of building design to connect occupants’ well-being to sustainability in design. To achieve this connection and bridge the gap between architectural design and occupant well-being. A thorough study of the WELL Building Rating System is submitted to resolve occupants’ issues within buildings in Egypt, along with analysing standard office design strategies and new trending office designs. To reach the paper’s aim, the following objectives will be achieved: Study and analyse the new WELL Building Standard rating system seeking the health and well-being of people, and understand all methods developed by IWBI to conduct an applicable design scenario solving problems and enhancing efficiency in existing office buildings in Egypt.

### 1.3 Study design

This paper will follow four parts:

**Literature review:** Involves studying the definition of well-being and connecting the relationship between it and employees productivity, what are the methods to measure productivity, what are the main points that lead to achieving well-being and reaching productivity, and studying the WELL Building Rating System, which focuses on the study of people and how to make them thrive, to reach a design scenario to improve the performance, productivity, and health of building occupants in existing office buildings.

**Qualitative study:** Performed to see how different buildings and companies implement different design strategies to improve the overall health performance of their employees and how it all reflects on their productivity. The analytical part will include analysing the WELL framework and studying existing examples of certified office building to understand how to improve the design strategies in Egypt to increase productivity.

**Quantitative study:** Concluding an easy applicable design scenario to be implemented in Egypt’s existing office spaces designed to improve productivity and well-being of employees and implement it in the case study office.

**Practical part:** Implementing the design strategy on a single office in the selected case study typical office building, in Alexandria/Egypt, using the computer software DesignBuilder to compare the simulation results between the base case scenario of the case study and the conducted design scenario. In addition to verifying the results data using the” Coefficient correlation parameters”. DesignBuilder is a paid computer software that has a free 30-day trial period. Alternatively, a free version of TRNSYS is available to use.

### 1.4 Methods

The research focuses on the well-being of occupants in existing office buildings in Alexandria, Egypt, and highlights a typical five-story office building, constructed in 2000 as a case study. The rationale behind selecting this office building as the case study for this paper is its representation of typical modern office buildings in Egypt, facing similar design/architectural challenges. Full curtain wall facades and inadequate ventilation, leading to thermal heat gain and an increased load on the HVAC system tasked with cooling the space, are prevalent issues in these buildings.

The methodology is formulated to establish an easy implementation scenario aimed at enhancing the productivity and well-being of occupants in existing office buildings in Egypt. This is achieved by understanding the definition of well-being, its relation to productivity, and the methods of measuring productivity. The WELL building rating system’s concepts and features are studied to identify those with a direct impact on employee productivity. Six concepts were identified as having a direct impact on productivity: air, light, thermal comfort, movement, sound, and mind. To further narrow down the three most impactful features, the evolution of office form is analysed, attributes of productivity in office space are examined, and two high-performing office buildings, Googleplex HQ and Amazon Spheres, are explored. These investigations aim to understand their productivity-driven design criteria and the reasons for their success. From these examples and the identified impacting WELL features, the top three design features affecting productivity are determined. To suggest easily applicable methods for existing office buildings in Egypt, three WELL-certified office buildings (the Centre of Sustainable Landscapes office building, the American Society of Interior Designers HQ office, and the 425 Park Avenue office building) are compared. This comparison aims to analyse the impact of WELL certification on employee productivity, understand the application of WELL concepts in office spaces, and identify the easiest application methods. Finally, a design scenario is proposed for implementation in the selected typical office building in Alexandria, Egypt, that serves as the case study for this paper. The methodology of this study uses DesignBuilder computer software to simulate natural ventilation, lighting, and thermal comfort in the case study building in two stages:

First, simulation in the base case:

To ensure that the results obtained through the DesignBuilder software can be replicated, it is important to follow a systematic and rigorous process:

1. **Data collection:** Collect detailed and accurate data on the building’s geometry, construction materials, HVAC systems, lighting, occupancy, and other parameters that may affect its energy performance.
  Applied through: This data was obtained through site visits and interviews, resulting in pinpointing the case study office building design challenges faced by its employees and collecting the data needed to model the base case building (indoor air temperature, wall thickness, heights, window types, glazing types, percentage of operable windows, plaster material, etc.)
2. **Model creation:** Use the DesignBuilder software to create a detailed and accurate model of the building, considering all the relevant parameters and inputs.
  Applied through: This process entails the input of data collected in step 1 and the adjustment of various simulation parameters, such as building orientation, window size, and placement. In this paper, a model is created of a base case five-floor building with a total construction area of 3370 m ^2^. The building is rectangular, with dimensions of 60 m × 67 m and a height of 20 m. It features exterior brick walls, uninsulated and 20 cm thick, with 2 cm of interior and exterior plaster, resulting in a total U-value of 1.5 W/m ^2^K. The floors are 4 m high. The main structure of the building is reinforced concrete, with uninsulated fixed curtain wall elevations that lack external shading and utilize a mechanical ventilation system. The flat roof, made of 20 cm reinforced concrete, is insulated and features four single-glazed skylights covering a total area of 240 m ^2^ above the main court.
3. **Calibration:** Calibrate the model by comparing its predicted energy performance against the actual energy consumption data for the building and adjusting the inputs as necessary to improve the model’s accuracy.
  Applied through: This procedure involves the iterative adjustment of input parameters, execution of the simulation, and comparison of outcomes with actual data until the model is accurately calibrated. In this case, the calibration ensures that the indoor air temperature closely mirrors real-life conditions. This is achieved by measuring the interior air temperature in the case study building using a thermostat and comparing it to the base-case model indoor air temperature. The results are expected to align closely.
4. **Sensitivity analysis:** Conduct sensitivity analyses to determine the sensitivity of the model’s results to changes in the input parameters and identify the most important variables that affect the building’s energy performance.
  Applied through: This step helps identify the most important variables that affect the building’s energy performance and provides insights into how the model can be improved. In this case, Application of operable windows, shades, and double glazing (Air, Light, Thermal comfort).
5. **Verification:** Verify the accuracy of the model by comparing its predicted results against independent data sources, such as published literature or other validated models.
  Applied through: This step helps ensure that the model is reliable and can be trusted to provide accurate predictions. In this paper the air temperature of the base case simulation in Designbuilder is being validated to the interior air temperature measured in real-life in the case study office building (measured indoor air temperature from the site visit by using a thermostat in different months throughout the year) by using the “correlation coefficient” parameter to validate the results as correlation coefficient (R ^2^) which should range between -1 and 1. In this case, the result was **0.945** which is within the acceptable range of the correlation coefficient -1 and 1, thus indicating that the base case results are reliable.
6. **Documentation:** Document the model creation process, including the input data, assumptions, and any modifications made during the calibration and verification steps.
  Applied through: This documentation should include the input data, assumptions, and any modifications made during the calibration and verification steps. Proper documentation also helps to ensure that the model can be updated and maintained over time as necessary.

By following these steps, it is possible to create an accurate and reliable model of a building’s energy performance using the DesignBuilder software, and to ensure that the results obtained can be replicated and verified.

Second, simulating daylight, thermal heat gain, and natural ventilation after implementing the conducted criteria on the case study building to compare the impact results with the base case results.

Data input: Typical Alexandria office building base case, EGYPT region, energy code ASHRAE 90.1-2007, location simulation using weather data “EGY_AL ISKANDARIAH_ ALEXANDRIA_ETMY”, the yearly design temperatures using 0.4% dry-bulb cooling design temperature with maximum value 33.2 OC, minimum value 27.1, and a Coincident wet-bulb temperature value is 22.3 OC, the climate zone used in ASHRAE 2B.

The building activity template is “office buildings” as the ASHRAE 90.1 Settings for heating source is “fossil fuel”, the occupancy density (people/m ^2^) = 0.05 based on the building survey.

For the Environmental control the heating set point temperatures for heating is 20.0 °C, and heating set back is 13.0 °C, the cooling set point temperatures cooling is 26.0 °C, and the cooling set back 32.0 °C. the computers and office equipment supplied to each zone according to the building visit.

The building constructions for the exterior wall the U-value equals 2.094 (W/m ^2^-K), and for the internal partitions equals 1.490 (W/m ^2^-K), and the typical floor equals 2.353 (W/m ^2^-K).

For the opening the external windows layout using wall façade types for 40% vertical glazing ASHRAE 90.2 Appx with a single layer of generic Clear 6 mm glass panel.

The lighting system through ASHRAE classification is space-by-space method with HVAC template is fan coil unit (4-Pipe), Air cooled.

All these parameters also applied for the post implementation scenario expect proposing a shading device (Vertical and horizontal Louvers) for the curtain walls windows as described and switching single glazing to double glazing additionally adding 30% operable windows externally and internal windows to allow cross ventilation.

## 2. Literature review

### 2.1 Well-being in office space

The World Health Organization (WHO) defines health as encompassing not only physical health but also mental and social well-being and not being limited to the absence of illness or injury. ^20^ However, for many employees, going to work means sitting at a desk and earning a paycheck, leading to productivity and health issues. ^21^^–^^24^ As a result, companies struggle to find and retain qualified employees. Today’s workforce seeks opportunities to enrich their lives and well-being and make a difference in the world. ^25^ Successful companies recognise this and strive to create work environments that align with their employees’ evolving values and goals. However, the path to achieving this is not always clear. To address this, the International WELL Building Institute (IWBI) established the WELL Building Standard, this framework integrates design and wellness to promote employee health and create workspaces where employees can flourish. ^26^

#### 2.1.1 Relationship between well-being and productivity in offices

**Figure 1.**
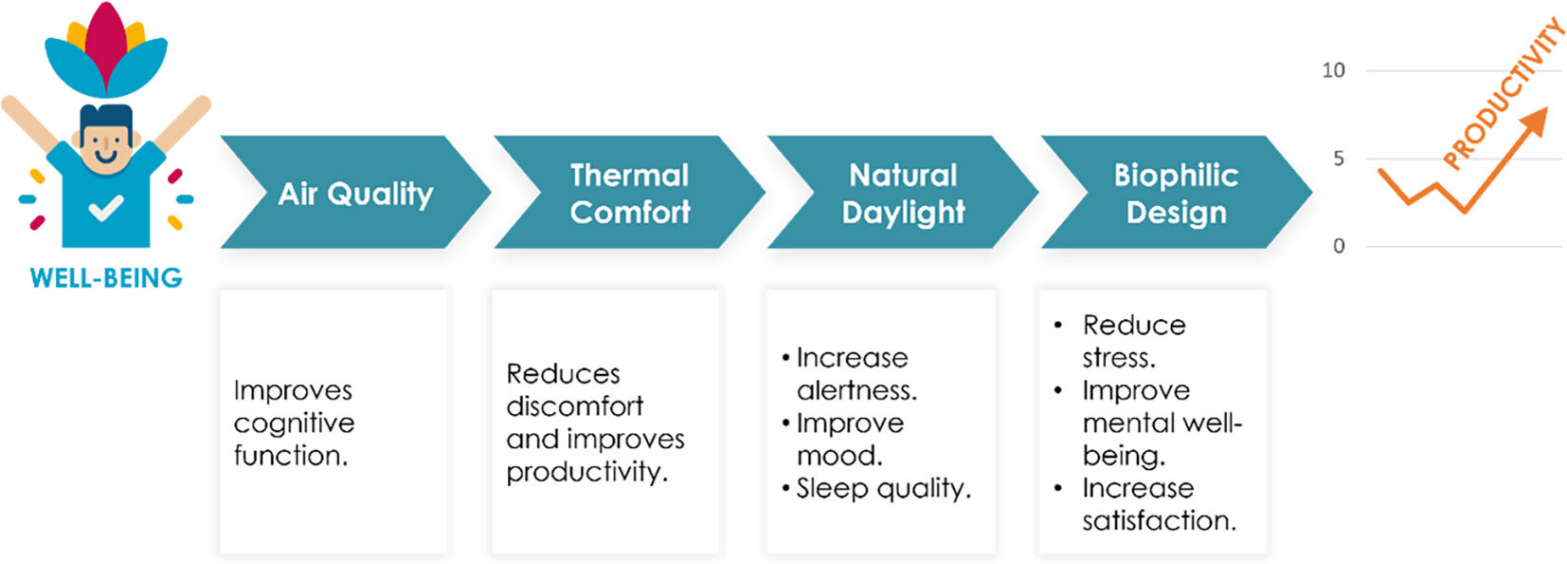
Steps to achieve high productivity rates in office space: author.

There is a strong relationship between well-being and productivity in office spaces ( Figure 1). Studies have shown that employees who experience higher levels of well-being tend to be more productive, engaged, and satisfied with their jobs. ^27^^–^^31^ Workplace well-being is influenced by factors such as enhanced indoor air quality, comfortable physical and thermal conditions, natural light access, ergonomic design, good acoustics, and the incorporation of biophilic elements. By incorporating these elements into office spaces, job satisfaction can potentially enhance, decrease absenteeism, and thereby increase productivity rates. ^27^^–^^31^

Thus, promoting well-being in office spaces through thoughtful design and management can lead to significant benefits for both employees and organizations, as it positively impacts productivity and overall job satisfaction.

#### 2.1.2 Productivity measuring methods

Employee productivity significantly impacts profits, yet it isn’t something that can easily be measured, and it’s not a one-size-fits-all rule to follow. Meanwhile, business companies are familiar with eight productivity calculation methods to measure productivity of their employees, independent of employer opinions, which are: data collection, labour productivity formula, percentage of goals met, determination of the overtime percentage, revenue per employee, employee absenteeism rate, learning ability, and percentage error rate. In the context of this paper, we will thoroughly investigate three widely used productivity measuring methods: Data collection, revenue per employee, and employee absenteeism rates. These methods are frequently utilized by companies to quantify their employees’ productivity enhancements and will be central to our discussion and examples. The remaining five methods will not be the primary focus of our analysis in this paper.

A. Data collection: such as physical features, outcome metrics (e.g., physical complaints) and HR department data (e.g., worker attitudes, performance data, absenteeism, medical costs, retention rates etc.), as well as financial directors’ data concerning revenue and financial metrics. These can be used to compare and calculate the overall effect on employee productivity. ^32^
B. Revenue per employee: The organization’s revenue can also be used to measure the productivity of employees. Although this isn’t a direct indicator of productivity, high revenue per employee can be seen as an indication of higher productivity in the workplace. ^32^

Revenue Per Employee = (Revenue generated in a particular interval of time/the total number of employees or the number of employees in the department whose productivity is aspired to calculate).

For example, if an organization has 50 employees and generates an annual revenue of $500,000 then:

Revenue Per Employee: ($500,000/50) = $10,000 per employee.

C. Employee absenteeism rate: The productivity of a workforce can be significantly impacted by the rate of employee absenteeism, given that productivity is essentially zero when employees are not present. Therefore, to enhance employee productivity, it becomes crucial to minimize the absenteeism rate within the workplace. The calculation of the absenteeism rate can be achieved using the following formula:

Employee Absenteeism Rate = (Total workdays lost to absenteeism/Total workdays in a given interval of time) *100.

For example, if an employee took 4 days off out of 30 working days, then:

Employee Absenteeism Rate: (4/30) *100 = 13.33%.

A rate of 1.5% is seen as being favourable but it does depend on the policies and regulations in place at various workplaces. ^32^

#### 2.1.3 What determines the state of health?

Research has demonstrated that over 50% of our well-being is impacted by our physical and social environment. This becomes particularly significant when considering that approximately 90% of our time is now spent indoors, thus positioning our environment as a crucial determinant of our overall health. ^2^^,^^5^^,^^33^^,^^34^ As the time spent indoors continues to increase, the role of buildings as a determinant of our health is underscored.

### 2.2 Well-being movement

While it’s challenging to pinpoint an exact date for the start of the movement focusing on the well-being of occupants in building design, it can be inferred that the emphasis on this aspect has been growing over recent years. The Healthy Building Movement, which focuses on the health and wellness of building occupants as a crucial component of high-performance buildings, underscores this shift. ^35^ Moreover, the concept of Biophilic Design, which argues that integrating nature-inspired elements into building and city-scale design has health, environmental, and economic benefits for occupants, also supports this trend. ^36^ However, it’s noteworthy that the awareness of architecture’s profound influence on occupants well-being has been recognized for decades, as Finnish architect Pallasmaa noted almost a quarter of a century ago. ^37^

With this said, new green building organisations resurfaced due to this trend targeting occupants’ health and well-being. One of the most know nowadays is The WELL Building Standard (WELL) developed by the International WELL Building Institute. Thus, it will be the focus of this paper. ^38^

The WELL Building Standard (WELL) created by the IWBI is a cutting-edge rating system that considers energy use, water consumption, waste production and other environmental impacts, as well as several socioeconomic measures. This has helped lead to the increasing global importance of green building construction and design that works towards creating a workspace where employees can thrive. ^39^

**Figure 2.**
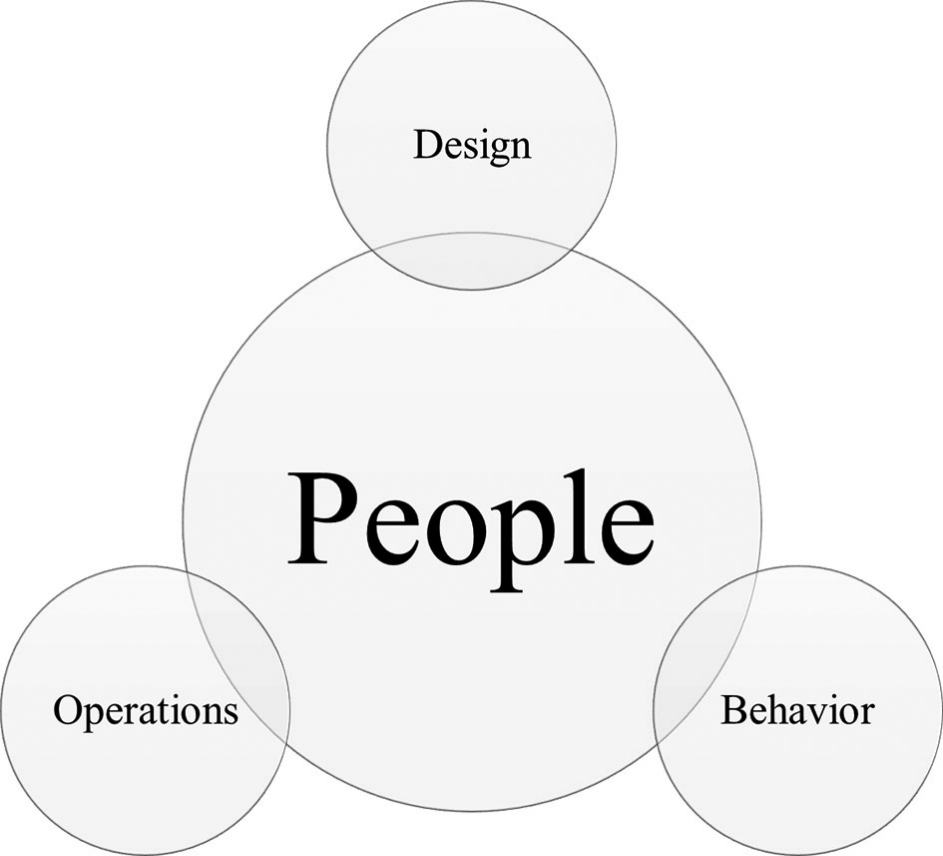
A diagram based on IWBI definition of WELL’s holistic approach: Author.

WELL is a holistic approach that needs the equal effort of four aspects: Design, Operation, Behaviour, and People for it to succeed ( Figure 2). WELL is designed to complement other top-tier green building standards while conducting thorough research into how the building environment can be improved for its occupants. As a result, projects are encouraged to seek dual certifications from both WELL and green building standards to achieve higher quality results.

### 2.3 Development of WELL

**Figure 3.**
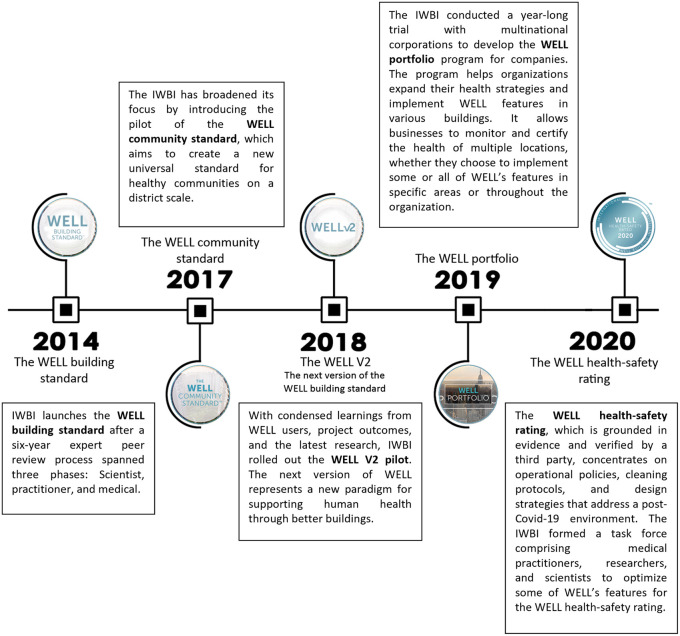
The timeline of WELL Building standard development: Author.

Back in 2014, WELL V1 consisted of 7 Concepts: Air, Water, Nourishment, Light, Fitness, Comfort, and Mind. It was rated by 3 scorings: WELL Silver Certification, WELL Gold Certification, WELL Platinum Certification ( Figure 3 for WELLs timeline).

**Figure 4.**
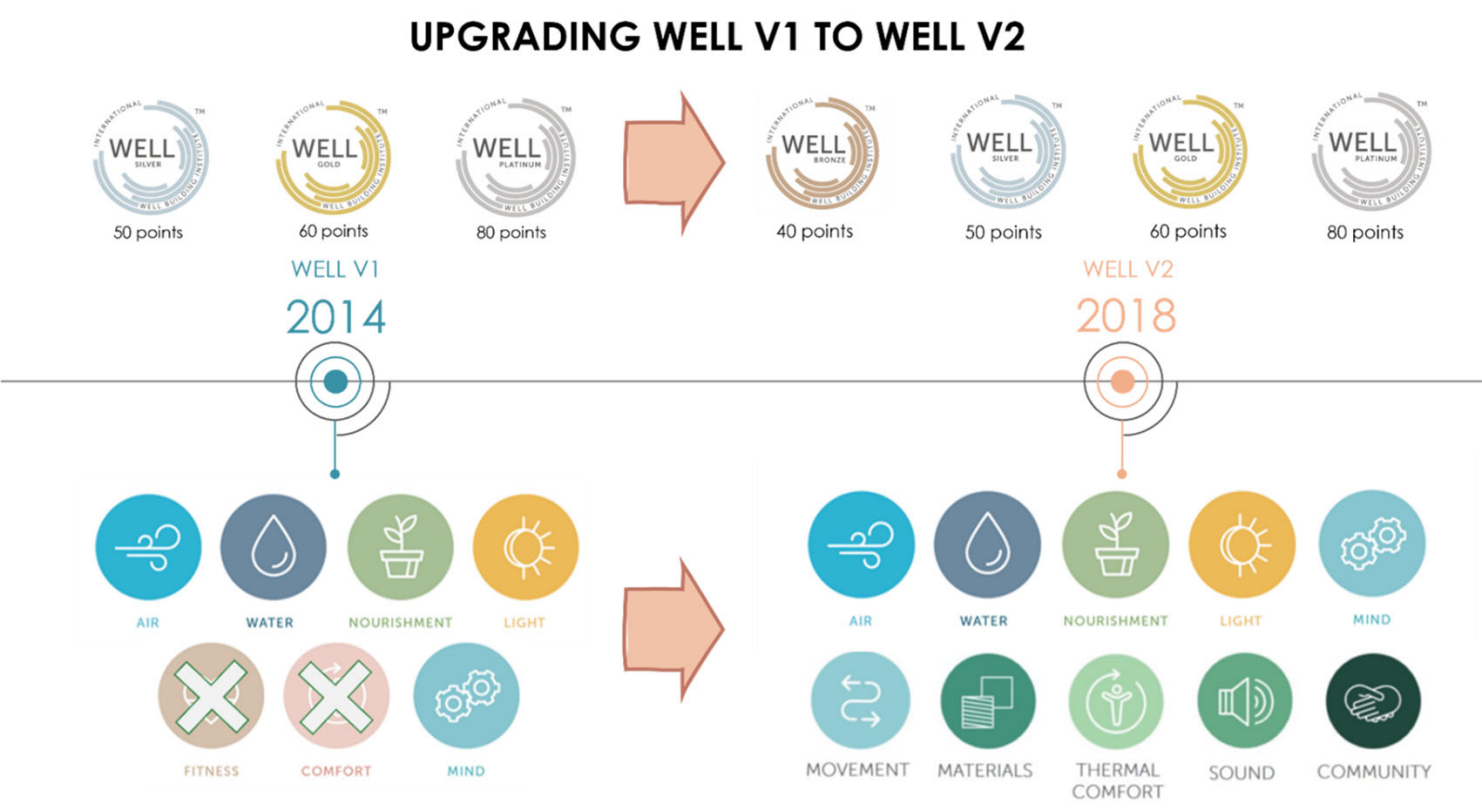
Modified WELL V1 concepts and scoring to WELL V2: Author

In 2018, after thorough research and collecting feedback from the users, IWBI modified WELL V1 which resulted in the development of WELL V2, where they concluded the importance of thermal comfort and its impact on the well-being of occupants. Thus, they developed a new concept based solely on thermal comfort. Additionally, adding four more concepts - Materials, Sound, Movement and Community Concepts. As a result, WELL V2 now consists of 10 Concepts: Air, Water, Nourishment, Light, Mind, Movement, Materials, Thermal Comfort, Sound, and Community ( Figure 4). Additionally, WELL introduced a new scoring system - the Bronze Certification - which led to four scoring options instead of the previous three, making it more feasible for projects to achieve certification. ^40^

#### 2.3.1 WELL V2 concepts and features

**Figure 5.**
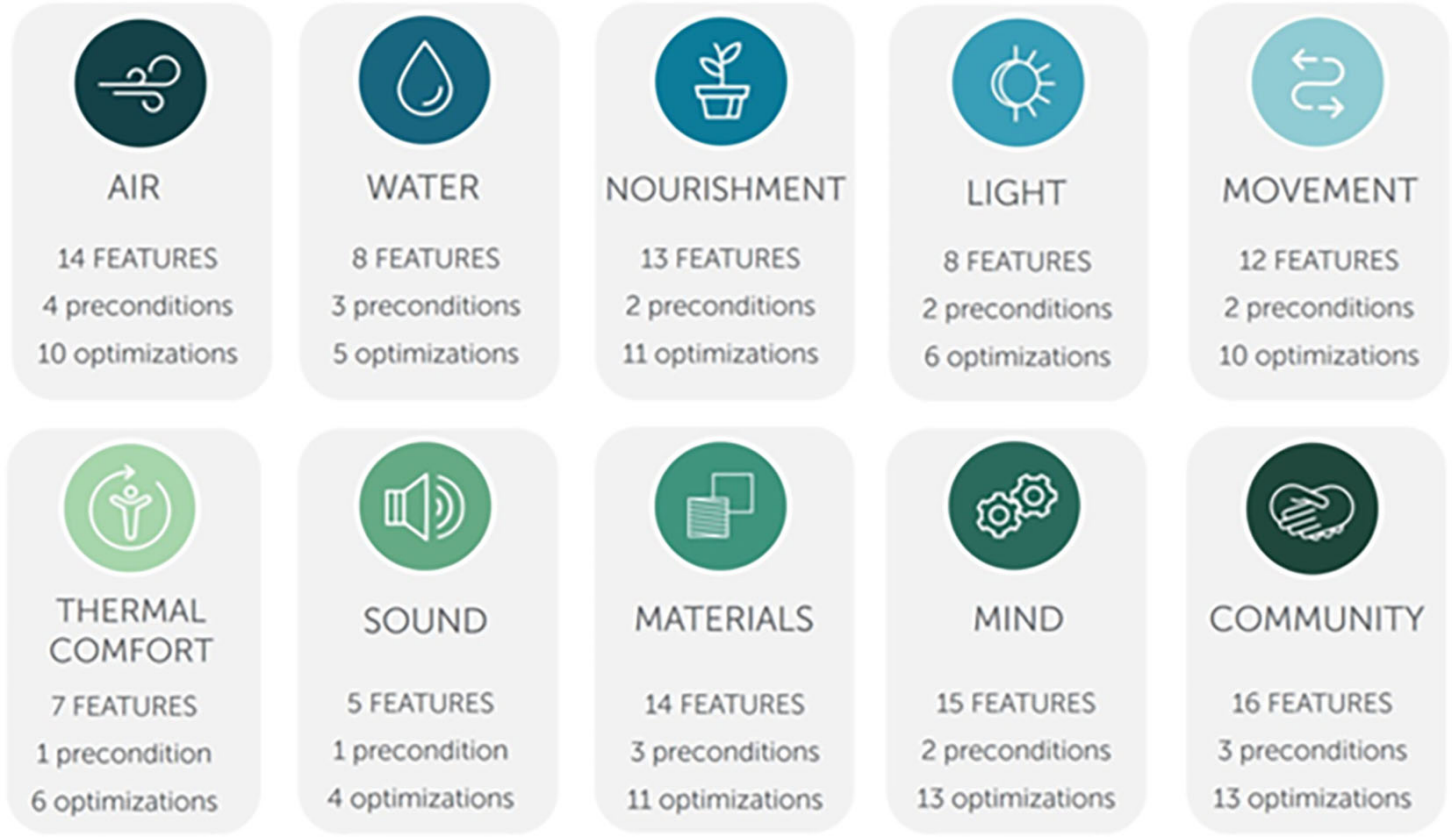
WELL V2 ten concepts showing the number of features (preconditions and optimizations) of each concept: IWBI.

WELL V2 consists of ten concepts, each concept is comprised of features with distinct health intents, totalling to 108 features divided into preconditions and optimizations ( Figure 5). Following a meticulous examination of the ten concepts within the WELL rating system, it was deduced that six of these ten are design-oriented and directly influence employee productivity. These concepts, namely Air, Light, Thermal Comfort, Mind, Movement, and Sound, will be subject to a more comprehensive exploration later in this paper to further pinpoint the top three impacting features on productivity.

**Air concept:** Good air quality has been linked to (absenteeism rate): Improved cognitive function, reduced symptoms of sick building syndrome, and fewer sick days among employees. All of which enhances the overall productivity of office employees and reduces employees’ absenteeism rate.

**Light concept:** Positive impact on employees’ circadian rhythms, mood, and alertness, in addition to reducing eye strain which helps employees maintain focus and productivity throughout the workday. ^40^

**Movement concept:** Reduce sedentary behaviour, and enhance cognitive function and mood, leading to increased productivity.

**Thermal comfort concept:** Uncomfortable temperatures lead to distraction, reduced focus, and decreased work efficiency. Thus, achieving thermal comfort reduces discomfort and improves employee’s productivity.

**Mind concept:** Helps employees’ recharge, and improves their concentration (maintaining focus), visual comfort, cognitive function, and regulates circadian rhythms while reducing stress and mental fatigue. All of which ultimately improves employee’s overall productivity rates.

**Sound concept:** The WELL Sound concept’s association with employee productivity is founded on its focus on enhancing acoustical comfort parameters. A well-managed acoustic environment in the workplace can improve concentration, reduce stress, and enhance productivity. Therefore, the Sound concept in the WELL certification plays a significant role in creating a healthier and more productive environment for employees.

Following this general introduction to WELL Building Standards’ six Architectural based concepts is the conducted design-oriented features with a direct impact on productivity.

#### 2.3.2 Conducted design-oriented features with a direct impact on productivity

**Air:** Choosing four key features from the Air concept, each with its own set of points, ensures these goals are met. The first feature is Enhanced Ventilation, where automated air conditioning systems should supply conditioned air through individual diffusers positioned 0.8 m above the occupants’ heads.

The second feature is Operable Windows, which provides access to natural ventilation when possible. At least 75% of occupied spaces should have operable windows of at least 4% of the floor area, and these should be designed with universal access in mind so they can be operated easily without tight grasping or twisting of the wrist.

Pollution Infiltration Management is the third feature, where the entryways of regular entrances (excluding terraces) should use entryway design elements such as grilles, grates, slots, or roll-out mats that have widths of at least 3 m and length in the circulation direction.

Finally, Source Separation examines separating rooms with high-volume printers, copiers, and humidity using automatic operating doors and negative-pressure exhaust fans that redirect outside air into higher-pressure areas. ^39^

**Light:** This concept is centred around the idea of light exposure with six selected features. The first feature is Light exposure focusing on the interior layout. 30% of all occupied areas must be within 6 meters of envelope glazing, and common areas must have seating for at least 15% of regular occupants, with a 5-meter distance between seatings and envelope glazing for 70% or more of said seating.

The second feature is Visual Lighting Design - 90% or more of space types in the project area must meet illuminance thresholds based on their purpose (offices need 320 lux at task surfaces while lobbies, atriums, and transition spaces need a minimum of 110 lux at floor levels). Eateries, lounge, and restroom levels are required to achieve a minimum of 110 lux at the task surface).

Circadian Lighting Design is the third feature - meeting lighting requirements for day-active people such as applying light levels on vertical planes at eye level, achieving 4 hours (min. start by noon) of light over work surfaces at 45 cm height and 140 cm in the centre of all seating areas and kitchens.

Electric Light Glare Control follows this as the fourth feature - buildings needing strategies to manage glare from electric lighting either through luminaire, considerations that limit UGR values to 16 or lower, and luminance do not exceed 6,000 cd/m ^2^ between angles 450-900 from nadir; or through space consideration where UGR values must also be 16 or lower.

Daylight Design Strategies is the fifth feature - two options present themselves: 70% of workstations within 7.5 m from transparent envelope glazing with VLT > 40%, 15% minimum envelope glazing area, or 70% within 5 m with VLT > 40%, 25% minimum envelope glazing area; both facilitated by solar shading in manual mode controllable by occupants (opening throughout the working day), automated shading for glare prevention.

Last up is Occupant Lighting Control - ambient lighting systems should be in place per 60 m ^2^, one per 10 occupants’ zones; differing criteria if rooms are smaller than needed or occupancy is lower than allocated quota; plus supplemental lighting available controlled by occupants. ^39^

**Movement:** This concept focuses on creating a healthy and comfortable working environment. There are four selected features in this concept. The first feature is Ergonomic Workstation Design which requires a minimum of 25% of workstations to be adjustable by users to support standing and seated positions. This includes flexible device heights, chairs, anti-fatigue mats or impact-reducing flooring, toe space, and footrests/footrails.

The second feature is Circulation Network which looks at aesthetically designed staircases with music, artwork, light levels, access to daylight and natural design elements for each floor. Visible stairs should be promoted over elevators and escalators from the entry-level onwards.

The third feature is Facilities for Active Occupants which provides cycling infrastructure with short-term bike parking located 30 m from the entrance accommodating at least 2.5% of visitors, and long-term bike parking located within building boundaries accommodating at least 5% of occupants. Furthermore, within a 200 m walk distance from the building boundary, there must be showers, lockers and changing facilities available for every 0-100 regular occupants, plus one shower per 150 occupants for every 101-999 regular occupants and 8 showers plus 1 per 500 occupants for every 1000-4999 regular occupant as well as 16 showers plus 1 per 1000 occupant for more than 5000 regular occupants with a minimum of five lockers associated with each shower facility.

The last feature is Physical Activity Spaces and Equipment, which requires the provision of an indoor activity space with dedicated fitness facilities offering two types of exercise equipment that can be used by at least 5% of building users, as well as outdoor physical activity spaces such as green spaces like parks or trails, blue spaces like swimming areas, recreational fields or courts and fitness zones. ^39^

**Thermal comfort:** This concept has six selected features that improve users’ thermal comfort. The first feature is Verified Thermal Comfort. The first point under this feature is a Thermal Comfort Questionnaire - occupants must participate in an anonymous questionnaire, and the number of responses required depends on the number of occupants: if there are more than 45, then a minimum of 35% should respond, 20-45 requires 15%, and fewer than 20 requires 80%. The results of responses must also meet target satisfaction thresholds: 80% or 90%.

The second feature is Thermal Zoning. The first point is to Provide Thermostat Control for at least 90% of occupied spaces; temperature in each room must be controlled via a thermostat or digital interface accessible via a smart device; maximum size per thermal zone should not exceed 60 m ^2^ or 10 occupants. Sensors should be placed at least 1 metre away from exterior walls, doors, windows, direct sunlight, air supply diffusers, mechanical fans, heaters etc.

The third feature is Individual Thermal Control. It includes two points. The first point provides Personal Cooling Options such as rooms/thermal zones with adjustable thermostats connected to building cooling systems that one person can regularly occupy; desk/ceiling fans; mechanical cooling system chairs; any other solutions capable of affecting a PMV change of -0.5 within 15 minutes without changing PMV for other occupants. The second point lists Personal Heating Options such as rooms/thermal zones with direct user-adjustable thermostats connected to buildings heating systems that can only be regularly occupied by one person; electric parabolic space heaters; electric heated chairs/footwarmers; any other solutions capable of affecting a PMV change of +0.5 within 15 minutes without changing PMV for others.

The fourth feature is Radiant Thermal Comfort, with Implement Radiant Heating and Cooling being the only point - at least 50% of occupied areas should have radiant ceilings/walls/floors or radiant panels attached, covering half the wall/ceiling area minimum.

Fifth is Enhanced Operable Windows, where Provide Windows with Multiple Opening Modes has four points: At least 70% open so no more than 1.8 m above finished floor (1 window per room); 30% open with whole opening 1.8 m above finished floor (1 window per room), Operation controls min 1.7m above the finished floor and low openings used in mild/warm weather, high in cold weather.

The last feature is Outdoor Thermal Comfort which consists of two points: Manage Outdoor Heat where pedestrian pathways and building entrances must have tree canopies, awnings or other structures providing shade for ≥50%, parking spaces ≥25%, plazas seating areas and other outdoor areas covered between 25%-75%; Avoid Excessive Wind where 5 m/s not expected more than 5% hours yearly in seating areas and 10% on paths and parking lots while 15 m/s no more than 0.05% hours throughout the year across all areas. ^39^

**Sound:** This concept has three selected features, starting with sound mapping as the first feature, offering an Acoustic Design Plan. Sound barriers are the second feature, with Doors and Walls Sound Isolation Design as its primary point. Lastly, Impact Noise Management is the third feature, with Specify Impact Noise Reducing Flooring being its main point. ^39^

**Mind:** There are five selected features in this concept. The first feature is devoted to promoting mental health and well-being with a dedicated space for restoration and relaxation, as well as work policies allowing breaks.

Nature and Place is the second feature that establishes a connection to nature through materials, patterns, shapes, colours, images, or sounds. It also entails celebrating culture and the integration of art.

The Restorative Opportunities feature, which is the third feature, provides a nap space and policy with at least one acoustically and visibly separated environment in a designated quiet zone, plus one reclining furniture for every 100 employees.

The Restorative Spaces is the fourth feature and offers an environment considering specific criteria such as lighting, sound, thermal comfort seating arrangements, calming colours, textures, and forms.

Lastly, the Enhanced Access to Nature fifth feature guarantees that 75% of workstations and seating areas have views of indoor plants/water/natural elements, and 70% of outdoor spaces include plants or natural elements within 200 m walk distance from the rooms available to occupants. ^39^

### 2.4 Conclusion

Following the completion of the analysis of the six design-oriented concepts within the WELL rating system, the intention is to identify the three design features exerting the most significant impact on employee productivity. To achieve this, a study examining the evolution of office space form will be undertaken, facilitating the identification of these impactful features.

## 3 Evolution of office space

### 3.1 Developed office design standards

**Figure 6.**
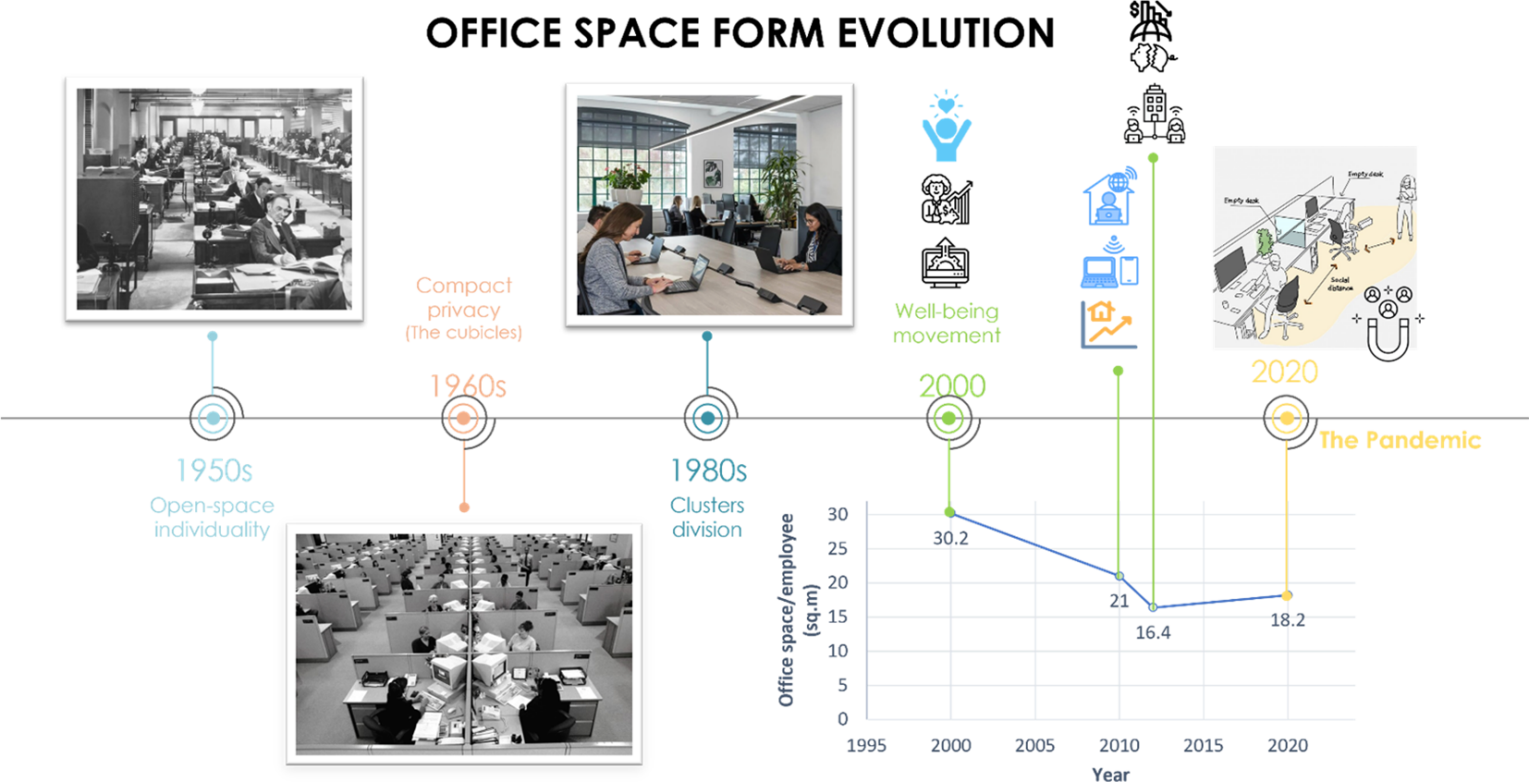
Office space form evolution: author.

The transformation of office space over the decades provides an insightful reflection of the changing attitudes towards work, productivity, and employee well-being. It was believed that office design and desk layout all influence the effect of office density over employees productivity and that the way density was measured can vary depending on the degree of enclosure (e.g., open plans and screens/partitions, etc.). ^41^ Thus, a thorough understanding of the office space form evolution is mandatory ( Figure 6).

The 1950s saw office layouts primarily as open spaces that promoted individuality and communal interaction. However, in the 1960s, the advent of cubicle offices provided employees with personal space and privacy, marking a shift towards. ^42^

In the 1980s, office layouts evolved to focus on clusters and departmental divisions, fostering intra-department teamwork while maintaining inter-department boundaries. At the start of the 21st century, the emphasis shifted towards employee wellness, resulting in office spaces designed to improve the occupants’ well-being. ^43^

However, the office space area per employee has been subject to fluctuations. In 2000, it was as high as 30.2 m ^2^, attributed to economic growth, technological advancements, and a focus on employee well-being. ^44^ By 2010, this number decreased to 21 m ^2^ due to the rise of open-plan offices, increased real estate costs, and technological advancements allowing optimised use of space. In 2012, it further reduced to 16.4 m ^2^, a 21% decrease, primarily due to the growth in flexible workspace trends and the global financial crisis. ^45^ In 2020, the COVID-19 pandemic necessitated social distancing measures, leading to an increase in office space per employee to 18.2 m ^2^. This change was influenced by social distancing, healthy building designs, and the need to attract and retain talent. ^46^ Taking into consideration that social distancing was applied by one of two methods by companies to maintain employees safety post-pandemic, since it was recommended to increase the size of the average workspace per person by 50%. ^47^ Some companies followed the ‘Hybrid work schedule’ method, which meant assigning half the employees to work remotely and switching shifts the next week while maintaining the same office footprint, while others choose to apply the distancing on a larger office or cutting of employees. ^48^

Post-2020, there was an ongoing debate about whether office space per employee should increase or decrease ^49^ ( Figure 6). Thus, The British Council for Offices suggested a generous allocation of space based on people rather than desks in order to satisfy companies’ current requirements for maximizing staff performance and comfort by providing a range of settings at work. ^50^ This report points out that with a 10-12 m ^2^ ‘Sweet Spot’ for each person, most common workspace issues like overcrowding and noise pollution can be addressed. On the contrary, higher office densities with less than 8 m ^2^ per person were more likely to cause complications and negatively affected occupants comfort, well-being and performance for most businesses. ^50^ Thus, companies realized the significance of their office setting. Creating a framework consisting of eight important design features to follow in order to design a healthy and productive workspace. ^51^^–^^53^ The eight conducted attributes of productivity in office space were location, size, appeal, well-being, flexibility, noise, good design, and user controllability.

### 3.2 Attributes of productivity in office space

1. Location: amenity-rich central location. ^51^^–^^53^
2. Size: 10-12 m ^2^ ‘Sweet Spot’ for each person
3. Appeal/Hotelization (e.g., relaxed dress codes, splash of colour, natural light, greenery, soft furnishing, Etc.). ^52^^–^^55^
4. Well-being/Atmosphere (biophilic design, distance between desks, areas for socializing, opportunities for fresh air, self-care amenities, private mother’s rooms, fitness amenities, things that are meaningful for the users. ^11^^,^^51^^,^^52^^,^^56^
5. Flexibility (e.g., open plan with flexible seating areas, Open and private offices, staff rotating schedules for remote and office attendance, ETC). ^11^^,^^48^^,^^51^^,^^54^^,^^56^
6. Designing the office by noise level (e.g., Public, Semi-Private, Private, adding a space for employees to unwind, ETC). ^48^^,^^54^^,^^55^
7. Good Design (Passive solutions, shading, natural ventilation, and daylight when possible). ^11^^,^^53^
8. User in control (giving occupants the control over their indoor environment). ^53^

However, to pinpoint the highest impacting features out of the previous list a thorough study of two successful office buildings, Googleplex HQ and the Amazon Spheres are to be analysed. ^57^^–^^59^

### 3.3 Successful office buildings examples

**Googleplex**

**Figure 7.**
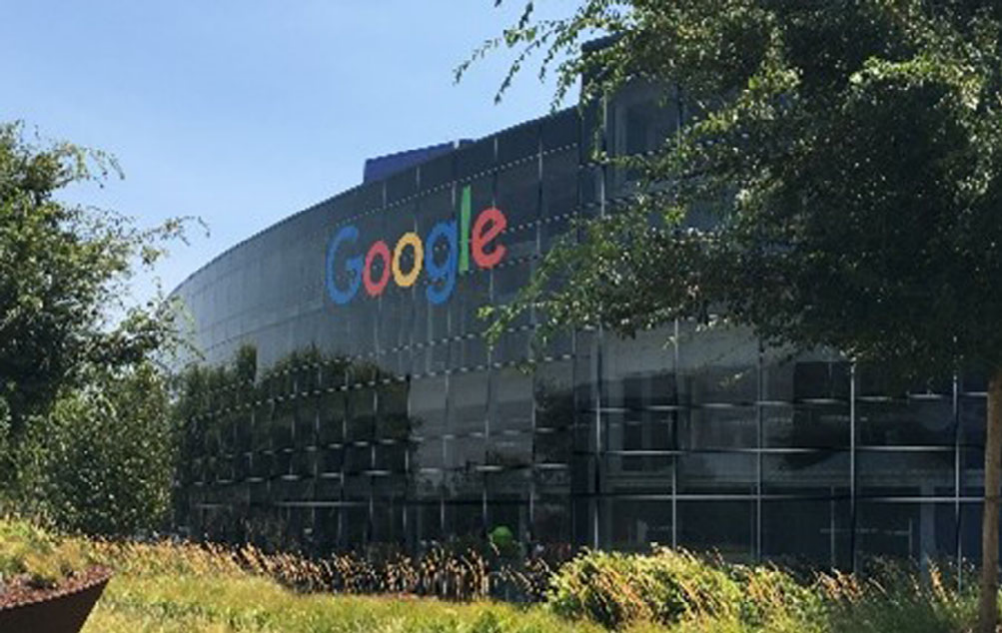
The elevation of the Googleplex building in California: Wikipedia.

With around 190 km ^2^ footprint of office space in California, USA ( Figure 7). Clive Wilkenson Architects Firm designed the building to mimic the university campus feel and merge the idea of the workplace with the experience and knowledge found within the educational environment. By applying 13 different office settings, the building components were proven effective in boosting employees’ well-being, ergo productivity. Google aimed to decrease everyday concerns and stress by providing all daily needs in one campus. ^60^ The building registered a 31% increase in revenue in 2013 which according to the productivity measuring methods proves increase in productivity, ^61^ meaning their philosophy benefited the company, thus it is selected as an example to be analysed ( Table 1).

**Table 1.** Summarized Googleplex architectural-design oriented features with most impact on enhancing the well-being and productivity of their employees: Author.

Design features	Additional detail
Air quality	• Operable windows. • The use of plants to purify air. • HVAC filters.
Biophilic design (Natural light)	• Natural light: the use of glass offices, open spaces, curtain walls courts and skylight for natural light access. • Glare control. • Sound proofing (sound zoning).
Thermal comfort	• Natural ventilation. • Shading devices: solar sensor exterior shading. • Building envelope design. • HVAC system. • Thermal zoning.

**Amazon Spheres**

**Figure 8.**
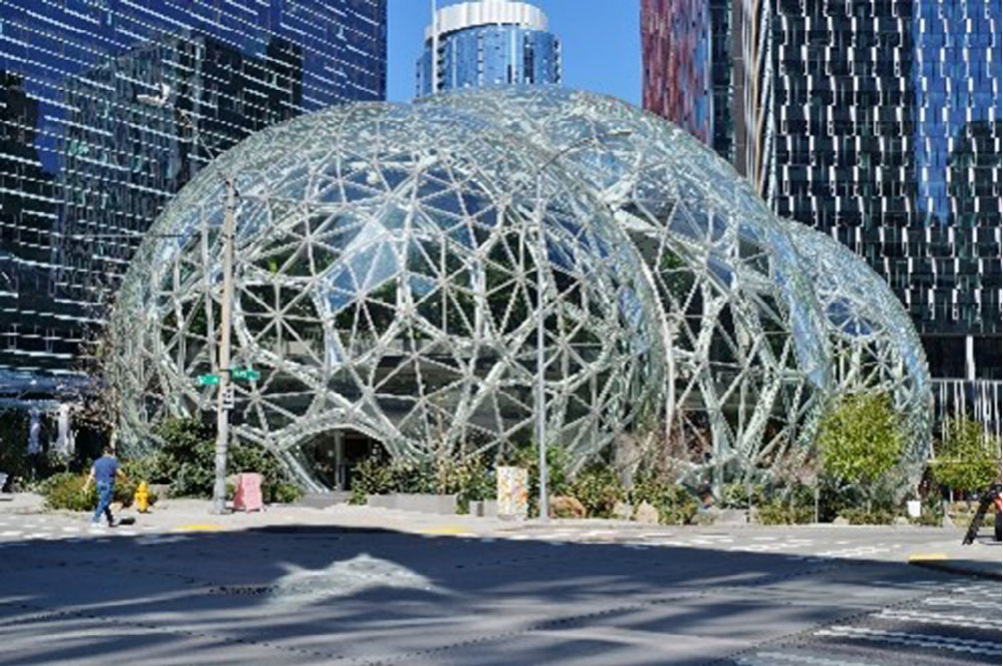
The Amazon Spheres three glass domes that are covered in pentagonal hexecontahedron panels: Wikipedia.

At 299.6 m ^2^ footprint located in Washington DC, USA ( Figure 8). ^62^ The Amazon Spheres was designed by NBBJ with the idea of mimicking the forest atmosphere while still being in touch with the urban areas’ comfort and luxury. After doing their research, they found that nature decreases stress, reduces cortisol levels and improves focus, and that’s how they came up with the building’s concept ( Table 2). ^62^

**Table 2.** Summarized Amazon Spheres Building design features aiming to enhance the well-being and productivity of their employees: Author.

Design features	Additional detail
Air quality	• Large court. • Plants purify the air. • Sophisticated HVAC system provides natural air every 20 minutes as the spheres façade doesn’t have operable windows to allow natural air.
Biophilic design (Natural light)	• Glare control. • Natural light: The Spheres’ Catalans support 2,643 panes of glass to provide ambient daylight access. • Circadian solution: Lights stimulating daylight. • Largest indoor living wall in the country with around 19m tall and 15m wide and hosts 25k individual plants. The building hosts 40k different varieties of plants (700 plant species). ^63^
Thermal comfort	• HVAC system. • Shading devices. • Building envelope design: pentagonal panels structure prevents solar heat gain; auto external shading tracks the sun.

### 3.4 Discussion of findings

By examining Tables 1 and 2, a replicated design criteria conducted by the two companies to enhance employee productivity was studied. It was observed that the same factors appeared in both the WELL concepts and the attributes of productivity, along with additional sources. ^17^^,^^64^ As a result, the three main design features that have a significant impact on employee productivity were identified as air quality, natural light, and thermal comfort. The subsequent step in this study involved comparing WELL certified office examples to determine the simplest application methods for incorporating these three features. This was done in order to establish an applicable scenario for existing office buildings in Egypt.

## 4. WELL-certified office spaces examples

**Figure 9.**
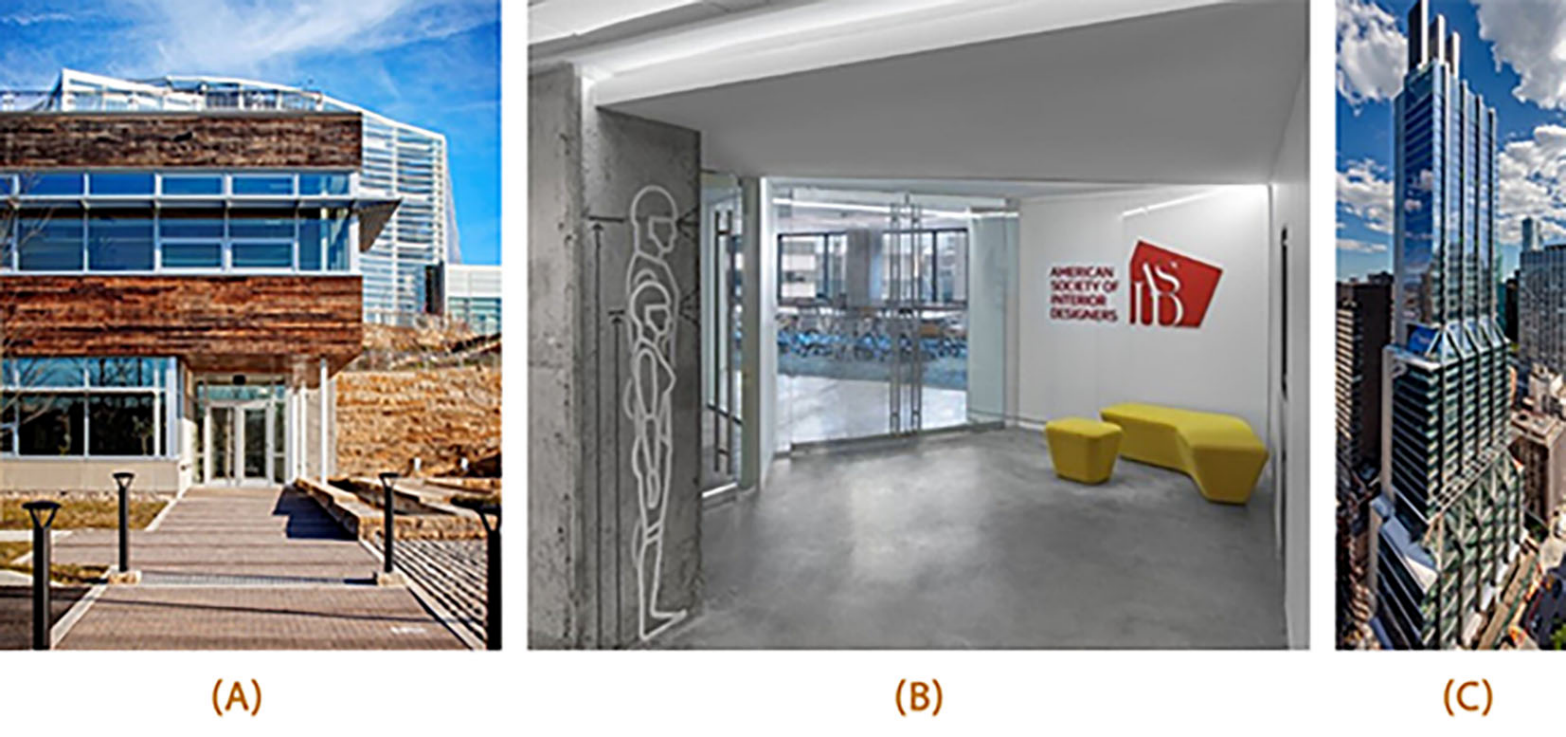
Picture (A) shows the entrance of CSL office building, acquired from PHIPPS conservatory website; Picture (B) shows ASID HQ office entrance, acquired from Architect - The Journal of the American Institute of Architects; Picture (C) shows the renovated 425 Park Avenue Highrise office building, acquired from Dezeen.

This section will focus on a comparative study of three office buildings certified by the WELL Building Standard, representing some of the best examples of sustainable and healthy workplaces ( Table 3) [ 1]. The Centre for Sustainable Landscapes Office Building (CSL), located in Pittsburgh, Pennsylvania, USA, is a net-zero energy building focusing heavily on the interaction between the building and the natural environment. The American Society of Interior Designs (ASID) headquarters office space in Washington DC, USA, is designed to promote employee well-being and productivity, with biophilic design elements and ergonomic workspaces. Lastly, the 425 Park Avenue high-rise office building located in New York, USA, is designed to focus on healthy building materials and energy efficiency. These three office buildings provide excellent examples of the successful implementation of WELL Building Standard principles in different contexts and building types ( Figure 9).

**Table 3.** Conducted design criteria for application on existing office in Egypt: Author.

Design Feature	CSL	ASID	425 Park Avenue	Easiest application
Air: Natural Ventilation	• Operable windows. • Court. • Operable skylight • Cross ventilation • Biophilic design	• Operable windows. • Greenery (Biophilic design) • Accessibility to operation of windows	• Breathing building concept. • Mechanical ventilation filtrations for city air pollution.	• Operable windows. • Court. • Operable skylight • Cross ventilation • Biophilic design • Mechanical ventilation filtrations for city air pollution.
Light: Natural light	• Curtain walls. • Court. • Reflective material for indirect daylight.	• Curtain walls. • Open plan layout and glass walls.	• Curtain walls. • Double and triple heigh floors to maximise natural daylight entrance.	• Curtain walls. • Court. • Reflective material for indirect daylight.
	**Shading**			
	• Exterior reflective shades • Interior solar shelves.	• Solar sensor shades over the curtain walls • Automatic interior blinds.	• Structure doubling as shading. • Canopies in some levels.	• Solar sensor shades over the curtain walls. • Interior solar shelves.
	**Circadian Rhythm**			
	LED Lights mimicking daylight			
Thermal Comfort	• Natural ventilation. • Occupants’ controllability. • High and low-level windows. • The use of clear and tented double glazing depending on the façade orientation.	• Natural ventilation. • Occupants’ controllability. • Mechanical ventilation.	• Natural ventilation. • High and low-level openings. • Mechanical ventilation.	• Natural ventilation. • High and low-level openings. • Mechanical ventilation. • The use of clear and tented double glazing depends on the façade orientation. • Occupants’ controllability.
	Shading devices.		Shading System.	

### 4.1 Selection criteria

All three examples are WELL-certified office spaces that acquired the certification as existing office buildings. Additionally, they all share the same Geometry façade style (curtain wall façade) that is applied on the typical office building in Egypt.

### 4.2 Comparison study of three WELL certified offices

The WELL certification has markedly influenced the architectural design of the CSL building, integrating numerous elements that prioritize occupant health and well-being. ^65^ The building uses natural light extensively to create an aesthetically pleasing environment that enhances the user experience. Additional features such as exterior and interior solar shelves and operable window blinds have been included to minimize glare and control sunlight penetration. The HVAC system is partitioned into distinct zones, providing users with full control over each zone’s timing, intensity, and duration. Furthermore, each occupied space in the CSL building has been designed to offer window views of the outdoors, contributing to an environment conducive to occupant health, well-being, and productivity. ^65^

Similarly, the ASID WELL certified office space has also had a positive impact on employee productivity through its sustainable and health-oriented design. ^66^ Employees have reported improved effectiveness and efficiency due to increased natural light exposure, reduced glare, and the implementation of an efficient circadian lighting system. ^67^ This improvement is reflected in the annual absenteeism score chart, which showed a 19% improvement from pre-certification (-0.025) to post-certification (0.16). Presenteeism scores, representing self-rated work performance, improved by 16% from 77.7 to 90. ^67^ The inclusion of plant life, improved air quality, and the overall design contribute to a physical and mental well-being-enhancing atmosphere, resulting in a more engaged and productive team. ^67^

The design of 425 Park Avenue office building was also impacted by the certification. ^68^ Mechanical systems with filters were used to purify the polluted city air. The building structure and service core were placed at the rear edge, away from Park Avenue, allowing for open, column-free leasable floors and increased natural light access. The Diagrid windows and the adjustment of artificial lighting to mimic natural daylight further enhanced the air quality experience for those working or visiting the office building.

Thus, after studying the three offices examples a scenario for easiest applicable features on existing office buildings in Egypt is conducted. Next, a simulation and validation of the conducted scenario on the case study building in Egypt will be done using Designbuilder computer software to clarify the impact.

### 4.3 Application on existing office buildings

Following the comprehensive analysis and review of previous examples, literature reviews, and various sources, it was concluded that the three most influential features for improving building occupant productivity have been identified. Based on this finding, a methodology was developed to implement these three features effectively, leveraging the features listed in the WELL rating system. The proposed scenario is primarily focused on enabling the implementation of these features in existing case study office buildings located in Alexandria, Egypt, with ease ( Table 4).

**Table 4.** Conducted design criteria for application in existing office building in Egypt: Author.

Feature	Application
Natural Daylight (Controlled Glare)	• Application of solar sensor exterior shading device over the building façades and interior reflective solar shelves.
Circadian Rhythm	• Providing supplemental light at the height of 45 cm above the work surface.
Natural Ventilation (Interchangeably with HVAC)	• Application of 25% operable window to the office building façades. • Placing a high-level window opposite to the curtain wall windows creates cross ventilation. • Operable skylight to allow natural ventilation
Thermal Comfort	• Application of automatic shading device over the building façades. • Providing two heights of operable windows: ○ Higher operable windows for cold weather. ○ Lower operable window for mild/warm weather. • Placing a high-level window opposite to the curtain wall windows and allowing air access from the skylight creates cross ventilation. • Adding double-glazing glass for the skylight.

**Figure 10.**
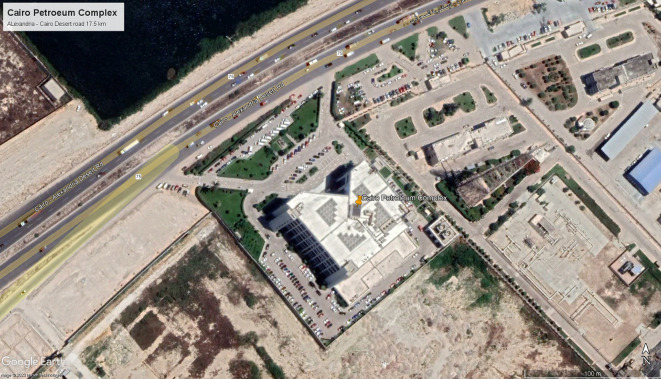
Bird’s-eye view of Cairo Petroleum Complex Building location: Google Earth.

## 5. Case study building

For the purpose of this paper, a typical medium-sized office building located in Alexandria, Egypt, exemplifying common office design problems prevalent in modern Egyptian office designs, was taken into consideration. This building, designed in 1995 and constructed in 2000, served as the case study. Permission to access the building and its blueprints was obtained, allowing for the measurement of natural ventilation, daylighting, and thermal gain. These parameters were evaluated through simulations conducted using DesignBuilder computer software for both the base case and following the implementation of specific criteria.

### 5.1 Brief

**Figure 11.**
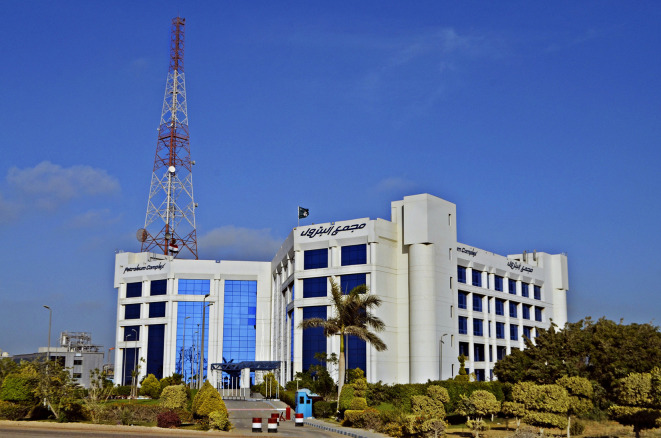
Cairo Petroleum Complex Building North-West main elevation showing the buildings Curtain wall façade: Wikipedia.

The case study building is composed of a total of 3370 m ^2^ area, with a rectangular shape measuring 60 m × 67 m and a height of 20 m, consisting of a ground floor and four typical floors ( Figure 11). It is structured using reinforced concrete, with blue double-glazed curtain walls and aluminium frames, and no exterior shading. It accommodates 600 employees arriving at 8 a.m. until 4 p.m. for their eight-hour shifts on weekdays, aside from the control rooms, which operate 24/7. Presently, the building rents offices to eight different companies and a bank. The offices are mechanically ventilated, with exterior walls constructed out of 20 cm brick, 2 cm exterior and interior plaster combination, and a total U-value of 1.5 W/m ^2^K. Meanwhile, the window-to-wall ratio across all elevations is 80%, except for the 90% that the North and Northwest double-glazed curtain walls have, with a U-value of 2.7 W/m ^2^K. Due to this, high mechanical loads are expected due to heat gain on the south and east-facing elevations. The northeast, northwest, southwest, and southeast elevations have 880 m ^2^, 198 m ^2^, 1072 m ^2^, and 960 m ^2^ of unshaded windows exposed to direct sunlight, totalling an overall exposed window area of 4010 m ^2^. The building contains four single-glazed skylights with a combined area of 240 m ^2^ and a U-value of 3.7 W/m ^2^K located above the primary court, with a flat, insulated roof with 3130 m ^2^ of reinforced concrete, interior paint, and insulation material with a U-value-of-0.5-W/m 2K (using the DesignBuilder computer software to identify the U-value within the building). ^69^

### 5.2 Building problems

**Figure 12.**
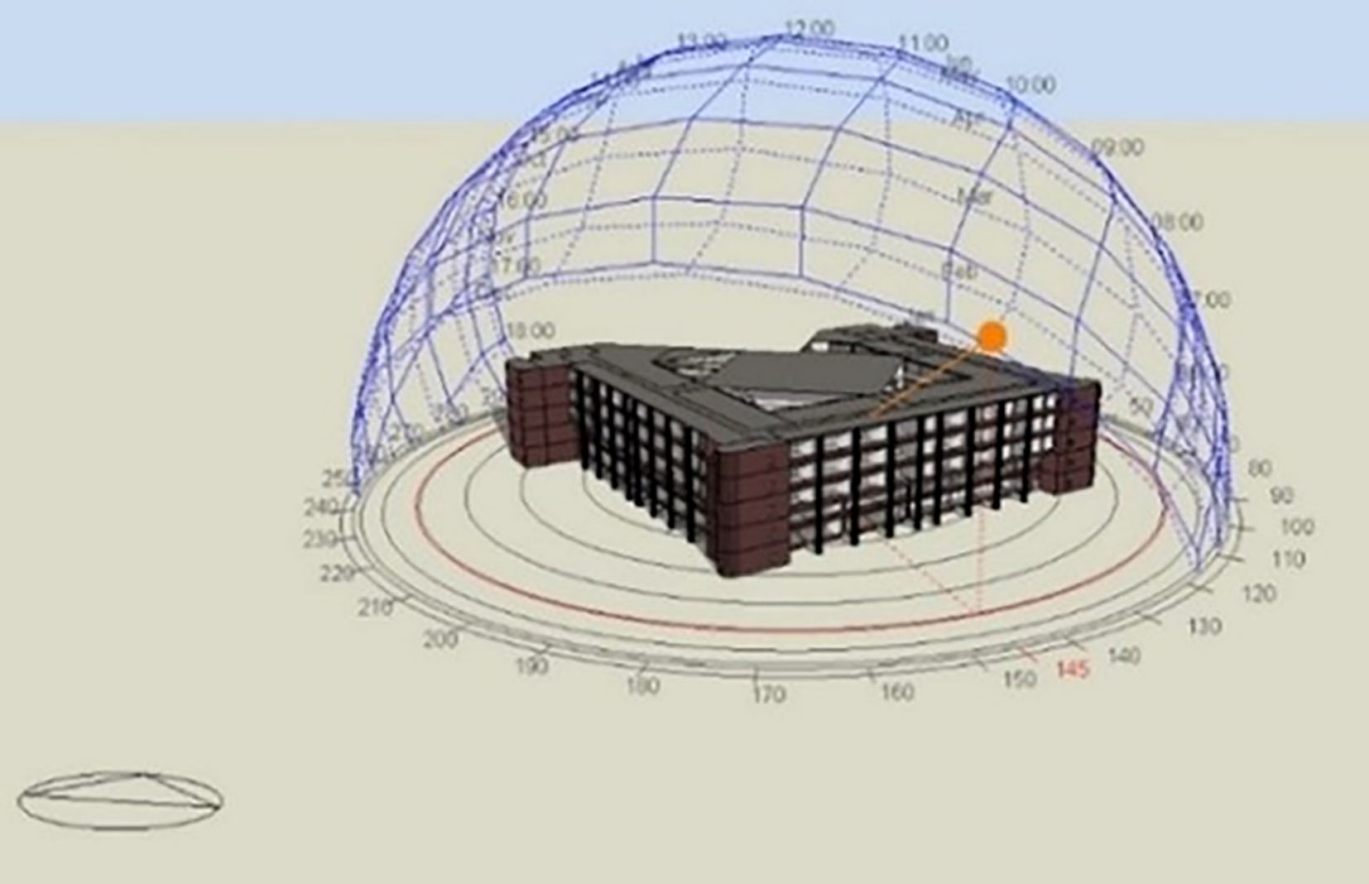
The three-dimensional model of the Cairo Petroleum Complex office building shows the solar path conducted by DesignBuilder on the selected office.

The case study office building is a prevailing example of modern architecture in Egypt, demonstrating an advanced mechanical ventilation system and a façade made mostly of curtain walls ( Figure 11). However, this combination has created various issues within the building due to thermal gain and glare caused by inadequate shading devices causing the West and South offices to be uncomfortable for employees to work in ( Figure 12 for solar path diagram and selected office). ^69^ Additionally, the building has inadequate natural ventilation as some facades have only 25% of the windows operable while others have none. Together these difficulties form a complex challenge requiring detailed consideration to ensure comfortable conditions with minimal energy consumption.

### 5.3 Designbuilder simulation

To validate the application of the proposed scenario, DesignBuilder computer software is utilized in this research. It serves to assess the impact of the scenario on the existing case study building. DesignBuilder ( Figure 12) is a tool used for building simulation and analysis. In the context of this research, the program has been employed for the following purposes:

• Evaluating the impact of design options on the building.
• Analyzing thermal comfort and indoor air quality.
• Conducting daylight and solar analysis.

#### 5.3.1 Base case model validation

A modelling validation method was needed to conduct reliable results for modifying the base case model based on the comparison between observation and simulation. Through various modelling validation parameters, “the Correlation Coefficient” parameter was selected. The Pearson product-moment correlation coefficient, also known as “the correlation coefficient [ 2],” is a widely used statistical tool that measures the strength of the relationship between two variables. The correlation coefficient ranges from -1.0 to 1.0 and indicates the relative movements of the two variables being measured. A value of 1.0 indicates a perfect positive correlation, while a value of -1.0 shows a perfect negative correlation. A value of 0.0 indicates no relationship between the two variables. Pearson’s correlation coefficient is denoted by R, with R2 representing the squared value of the correlation coefficient. It is commonly used in various scientific fields. ^70^

The formula states:

$$r=\frac{n\left(\sum \mathrm{xy}\right)-\left(\sum x\right)\left(\sum y\right)}{\sqrt{\left[n\sum x^{2}-{\left(\sum x\right)}^{2}\right]}\left[n\sum y^{2}-{\left(\sum y\right)}^{2}\right]}$$

“ *r*” stands for “the *correlation coefficient*”, “ *n*” stands for “number *in the given dataset*”, “ *x*” stands for “ *first variable in the context*”, and “ *y*” stands for “second *variable*”.

**Figure 13.**
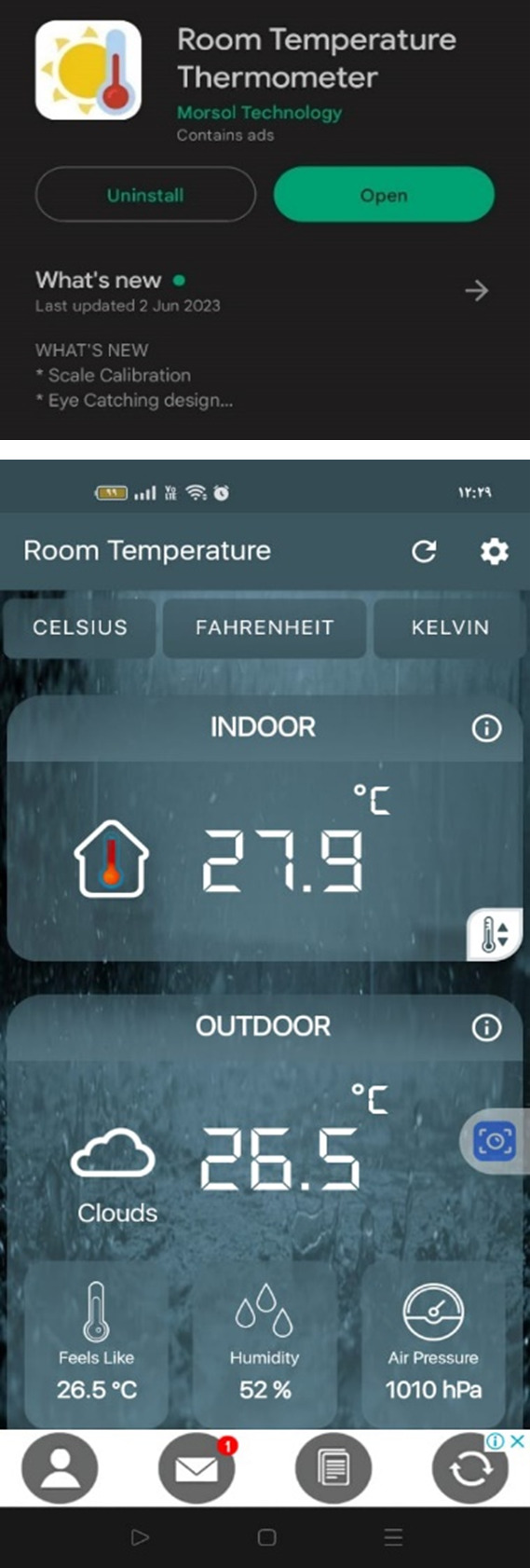
Room Temperature Thermometer android mobile application used as a validation method to measure indoor air temperature inside the case study building. The screenshot shown is taken for June month measurement: Author.

By comparing the interior air temperature data gathered from site visits to the case study office building through the months May, June, July, and August of 2023 and the base case resulting data from DesignBuilder software, the following table is conducted:

By using the variables from Table 5 in the Correlation Coefficient formula, the following results are achieved ( Figure 13):

$$\text{The correlation coefficient}\;\left({\text{R}}^{2}\right)\;\text{is}:0.993$$

$$\text{Pearson’ correlation coefficient}\;\left(R\right)\;\text{is}:0.997$$

**Table 5.** Required temperature data of the months May, June, July, and August of the year 2023 gathered from the “Room Temperature” application and base case scenario temperature results for The Correlation Coefficient formula: Author.

Month	Observation	Simulation
May	26	27.84
June	27.9	29.85
July	28.1	31.35
August	29.4	32.84

**Figure 14.**
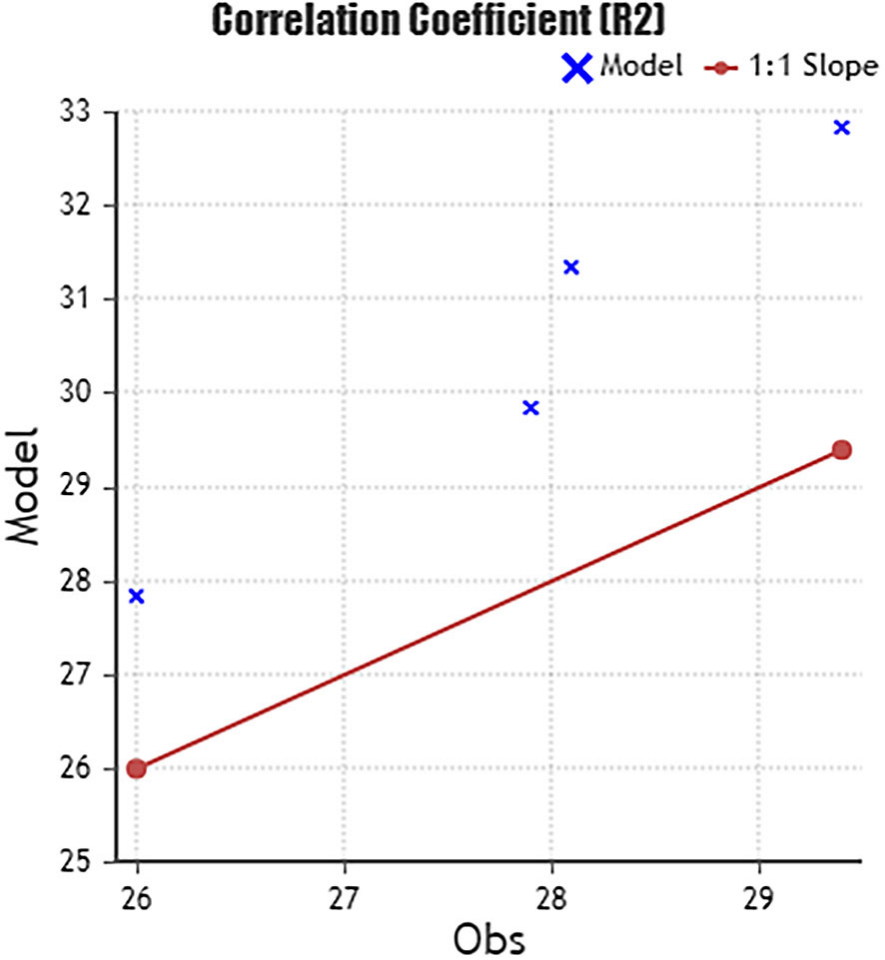
The Correlation Coefficient formula calculation chart.

The results show R ^2^ = 0.945, which is within the acceptable range of the correlation coefficient -1 and 1, thus indicating that the base case results are valid, reliable, and applicable.

### 5.4 Selected office and possible solutions

**Figure 15.**
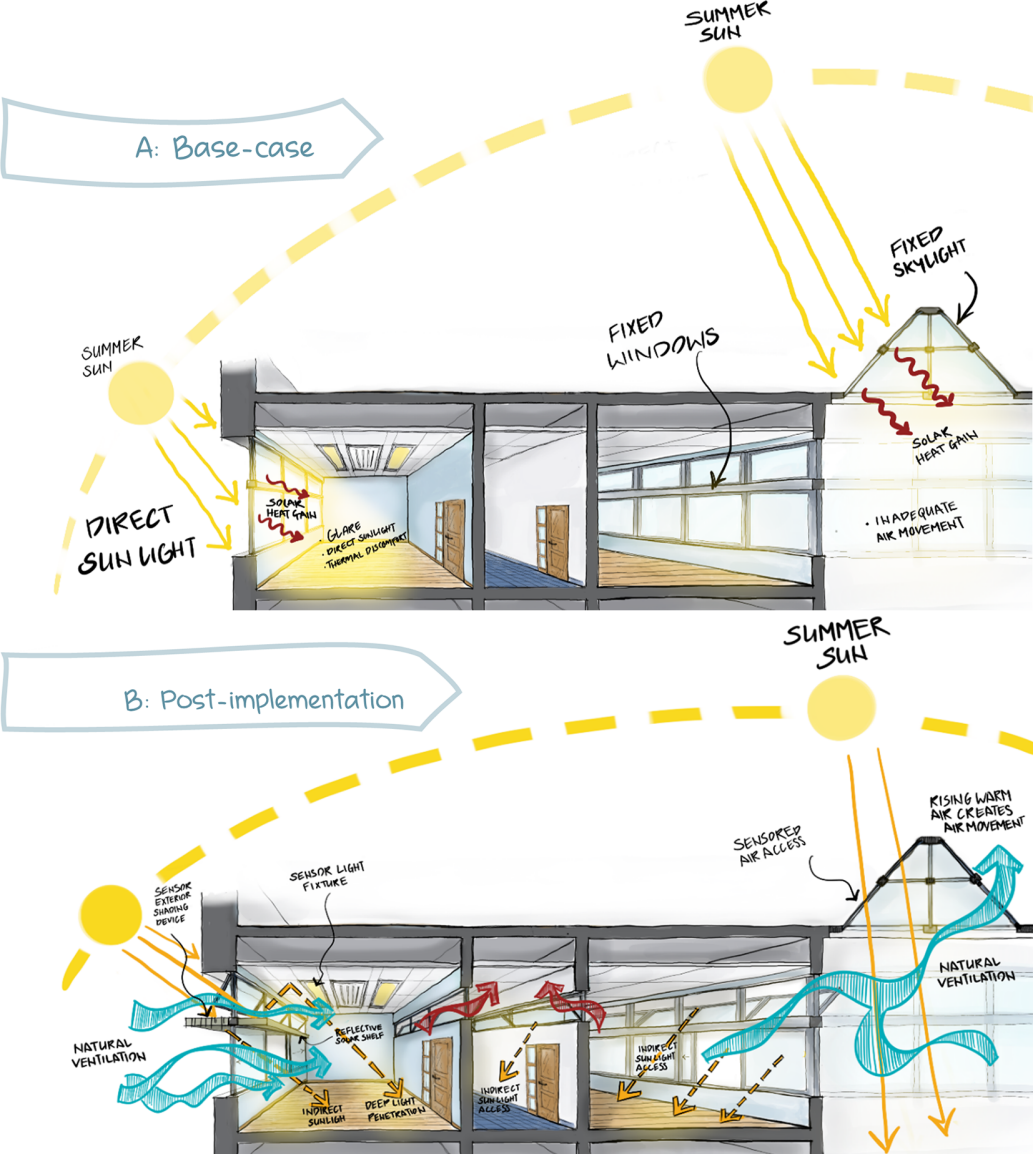
Picture (A) schematic sketch of the base-case scenario showing building problems: direct sunlight hitting the office area causing glare, discomfort, and solar heat gain, in addition to the fixed panels skylight and curtain walls that doesn’t allow air flow: author based on the base case model. Picture (B) schematic sketch showing the proposed scenario implementation on the case study building which shows an enhancing of ventilation and air flow in addition to light: Author sketches based on the proposed post implementation scenario and base-case scenario.

**Figure 16.**
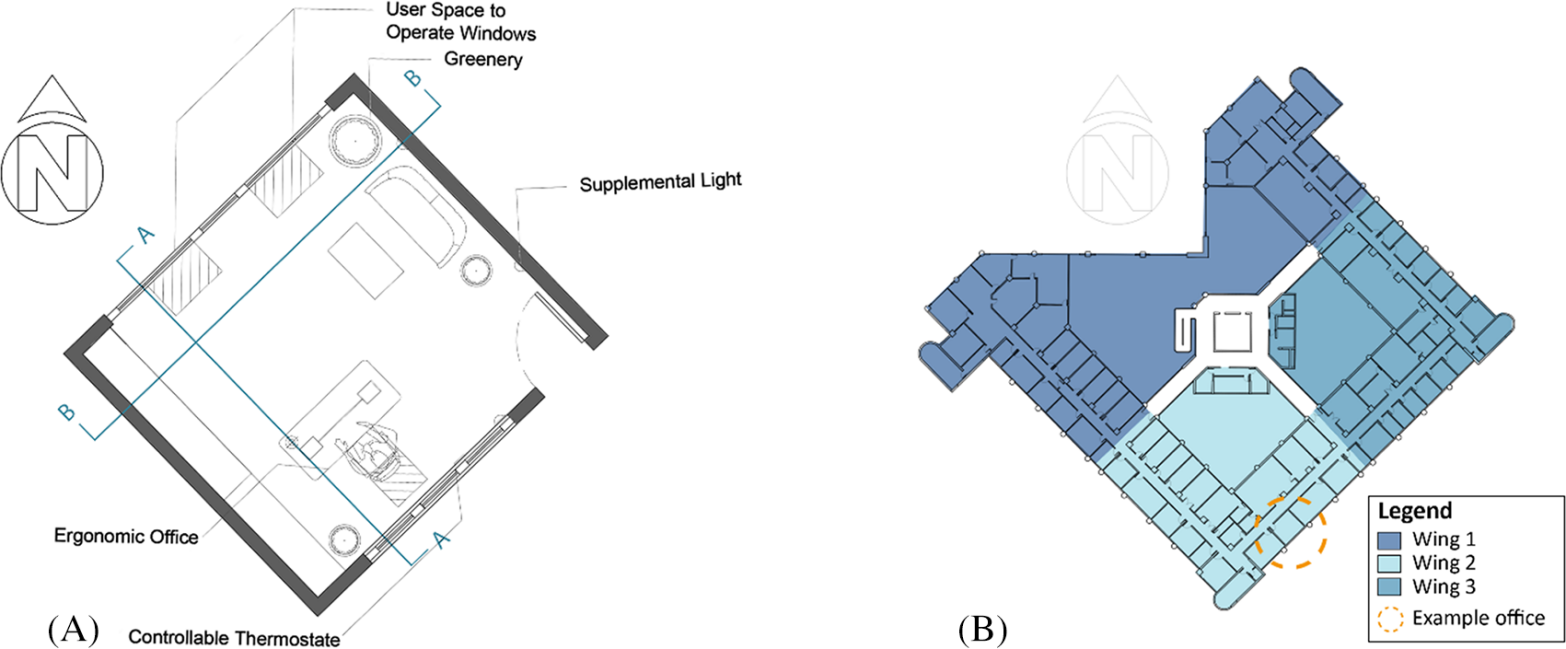
Picture (A) shows Implemented criteria on case study chosen office plan on the south-east façade, Picture (B) building typical floor plan showing the chosen office location and the three wings division: Author.

According to employees’ statement reported through interviews conducted by the author, the proposed scenario is applied from the previous chapters on the North-East and South-East façades that was reported to suffer from thermal heat gain from the exterior façade and direct sunlight. The following modifications are applied for a comparative simulation ( Figure 15 and Figure 16):

**Curtain Wall:** Designing different height operable windows in the façade to allow natural ventilation, higher operable windows for winter, and lower operable windows for summer. Operable windows must be opened at least halfway, with the maximum height from the finished floor not exceeding 1.8 m and a minimum dimension of 0.3 m for the smallest opening. Window operation control is minimum 1.7 m above the finished floor.

**Shading:** Application of solar-sensored shading devices (louvers) over curtain wall facades (South-East, South-West, North-East, North-West) to decrease direct sunlight entering the offices and eliminate glare. Additionally switching interior blinds with interior reflective solar shelves for deep light penetration.

**Cross Ventilation:** Applying a high-level operable window opposite to the curtain wall to create cross-ventilation.

**Skylight:** Provide automated operation of skylight panels to allow natural ventilation access. In addition to Insulated double-glazed panels.

**Circadian Lighting Design:** The necessary light levels should be achieved from a height of 45cm above the work plan and a sensory-activated LED light that mimics daylight.

**Figure 17.**
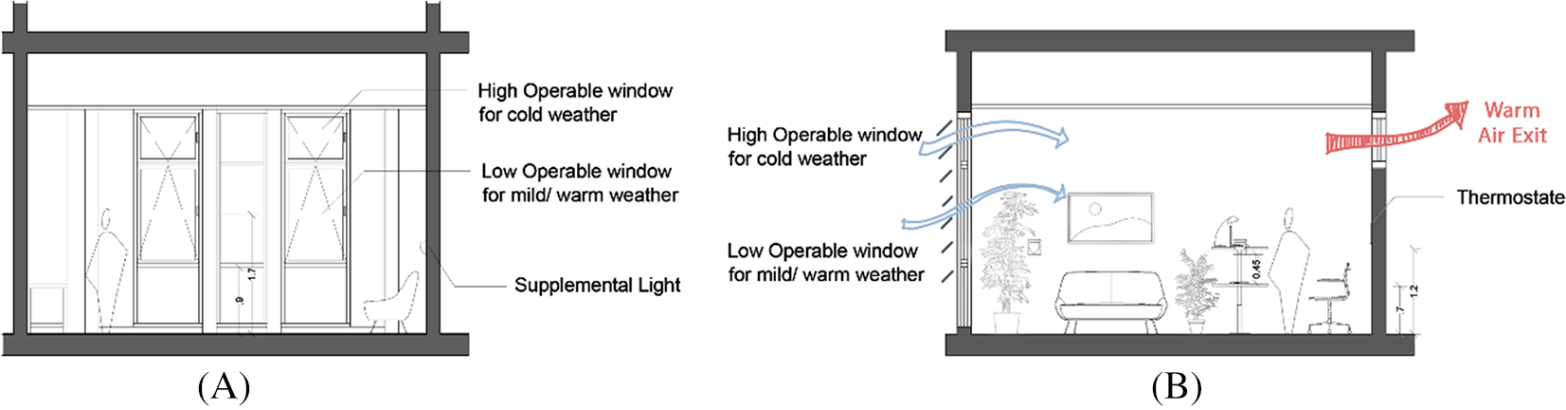
Picture (A) Cross Section B-B of case study office room showing the recommended heights for window operation management with higher operable windows use for cold weather and lower operable window for mild/warm weather, Picture (B) Cross section A-A showing predicted natural ventilation from different window heights and the circadian solution: Researcher’s work on the case study office building using Autodesk: AutoCAD software.

**Location of Furniture:** The distance between envelope glazing and seating area is within 5 m, VLT>40% to protect from direct sunlight. In order to measure the impact of the proposed scenario, we simulate the impact on the chosen selected office room oriented in the South-East façade ( Figure 16, Figure 17).

### 5.5 Simulation of base case and post-implementation proposal

The following analysis diagrams compare the office base case and post-implementation results. Starting with the direct sunlight simulation results. ^69^

**Figure 18.**
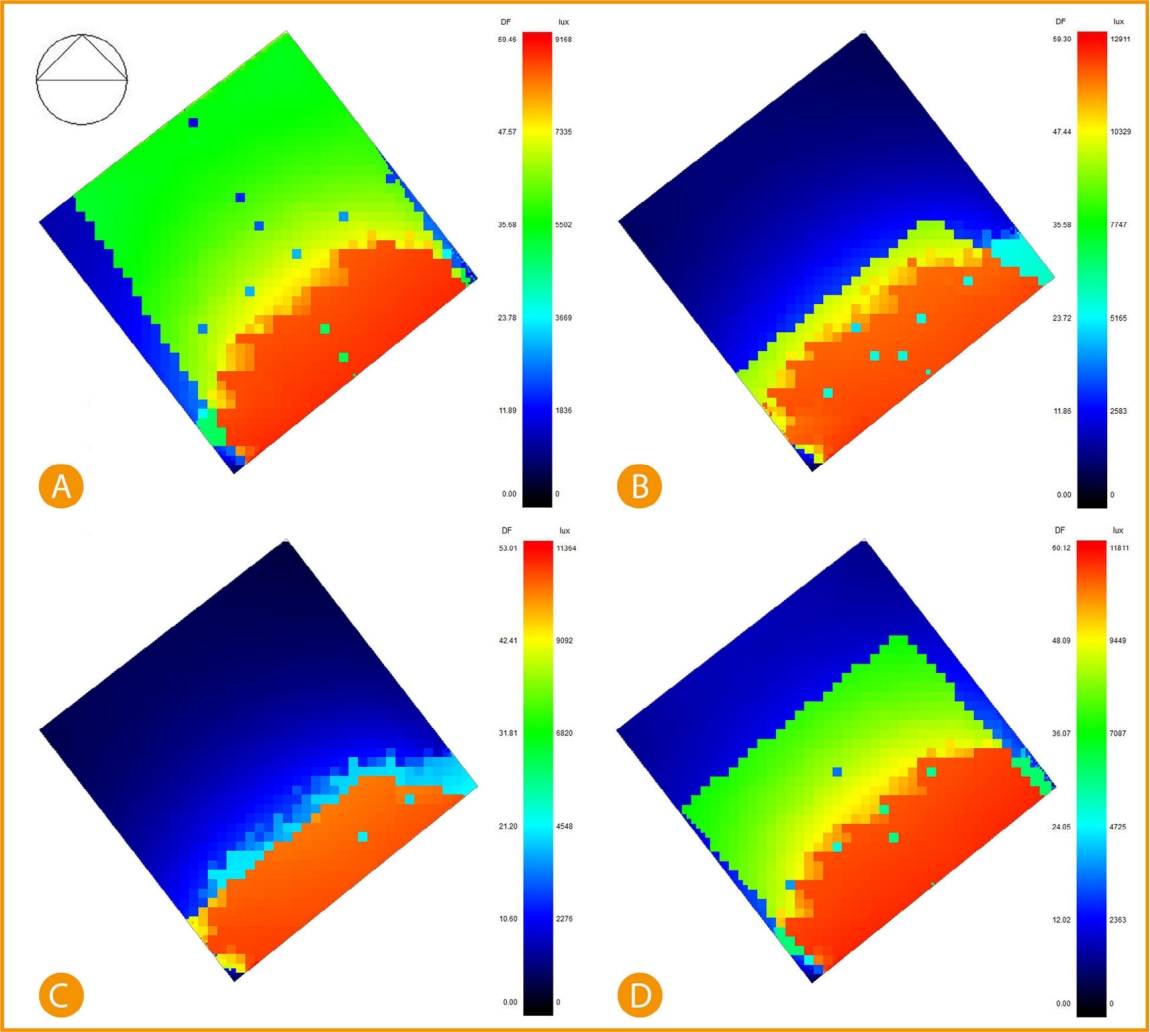
South-East office Base case direct sunlight simulation results, (A) January sunlight simulation, (B) April sunlight simulation, (C) July sunlight simulation, and (D) October sunlight simulation. The photos (A), (B), (C), and (D) were acquired from the researcher’s work on the case study office building using DesignBuilder software.

Using DesignBuilder computer software to simulate the base case of direct sunlight entering the office, we find that January and October are the highest recorded sunlight rate entering the building. January recorded direct sunlight of 7335-9168 lux reaching around 28% office space, while 60% is Indirect daylight of about 5502 lux, and 12% is daylight 0-1836 lux. April records 27.1% direct sunlight of around 12911 lux, 9.1% 10329 lux, 1.6% 5165 lux and the rest 62.1% between 0-2583 lux. July records 21.7% direct sunlight of 11364 lux, 7.9% records 9092 lux and the rest, 70.4%, records between 0-4548 lux. Lastly, October records 30% direct sunlight of 9449-11811 lux, 35% indirect sunlight of around 7087 lux, and the other 35% records 0-2363 lux ( Figure 18).

**Figure 19.**
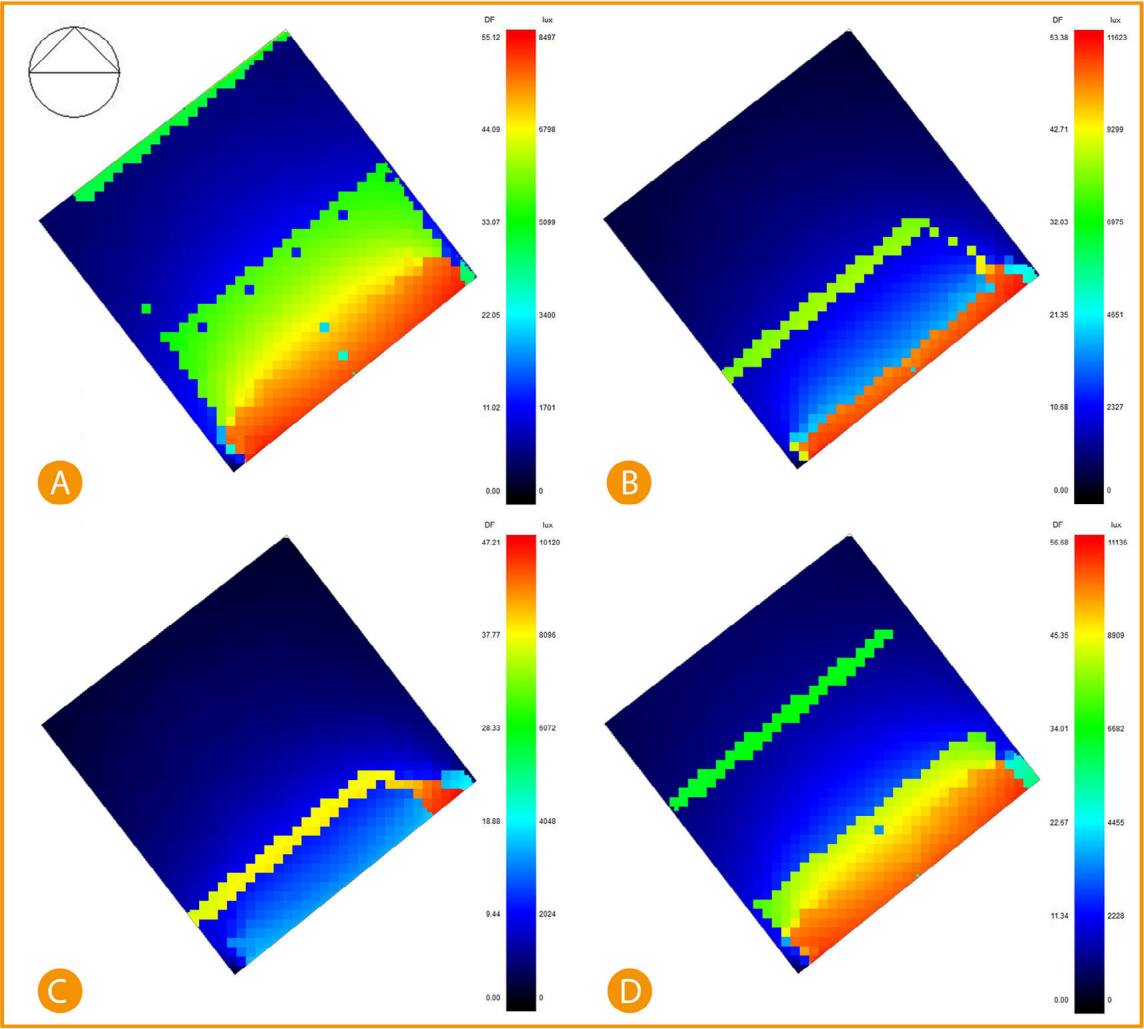
South-East office Post-implementation direct sunlight simulation results, (A) January sunlight simulation, (B) April sunlight simulation, (C) July sunlight simulation, and (D) October sunlight simulation. The photos (A), (B), (C), and (D) were acquired from the researcher’s work on the case study office building using DesignBuilder software.

While direct sunlight simulation post-implementation shows significant improvement and decrease in the area exposed to direct sunlight, the results were simulated after adding an automatic shading device (louvres) on the southeast façade. The results show January recorded 19% of exposed area in the range between 6798 lux to 8497 lux of direct sunlight, 27% records around 5099 lux, and the 54% remaining area are recorded at between 0-3400 lux, April records only 5.2% of the area is direct sunlight of 11623 lux, 5.9% records 6975 lux, and the rest 88.9% records between 2327-4651 lux. July records only 7.4% area of direct sunlight at 10120 lux, and the rest of 92.6% area is recorded between 2024-4048 lux. Lastly, October recorded 20% of direct sunlight area at 8909-11136 lux, 9.34% records 6682 lux, and 70.26% records between 2228-4455 lux ( Figure 19). ^69^

Comparing the base case and post-implementation results, it is visible that in January, there was a decrease in the total lux by 7.3% and a decrease in the direct sunlight exposure area by 9%. In April, the total lux decreased by around 10%, and the direct sunlight exposed area decreased by 21.9%. In July, total lux decreased by 10.9%, and the direct sunlight exposed area decreased by 14.3%. Lastly October, total lux decreased by 5.7%, and the direct sunlight exposed area decreased by 10%.

**Figure 20.**
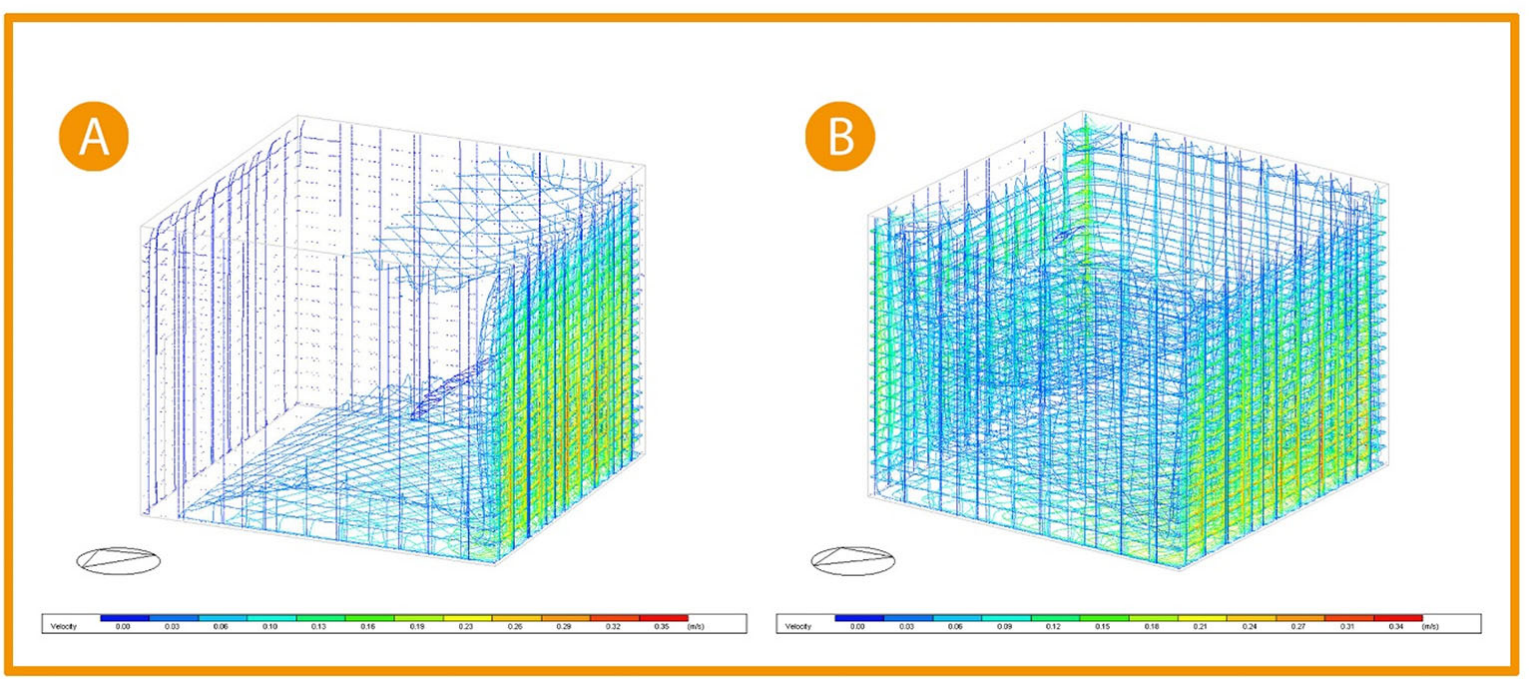
Three-Dimensional Air flow simulation comparison chart (A) Base case, (B) Post-implementation. The photos (A), and (B) were acquired from the researcher’s work on the case study office building using DesignBuilder software.

Thus, conducting through the previous studies that applying around 25% operable windows to the office façade and placing a high window opposite to the curtain wall windows creating cross ventilation, indicated an increase in air flow in the space ( Figure 20). ^7^

Picture (A) Figure 20 shows the base case three-dimentional simulation for airflow; the simulation indicates that the airflow is minor, and its velocity ranges between 0.01-0.10 m/s closer to the floor, increasing to 0.19 m/s near the curtain wall. While in picture (B), after adding windows to the façade and opposing it to create cross ventilation, the airflow increased to cover all the office area with a slight increase in the velocity ranging between 0.06-0.12 m/s all around the office while not affecting the comfort of users. The air velocity at the building envelope remains the same. Thus, making a 20% improvement in air flow. ^69^

**Figure 21.**
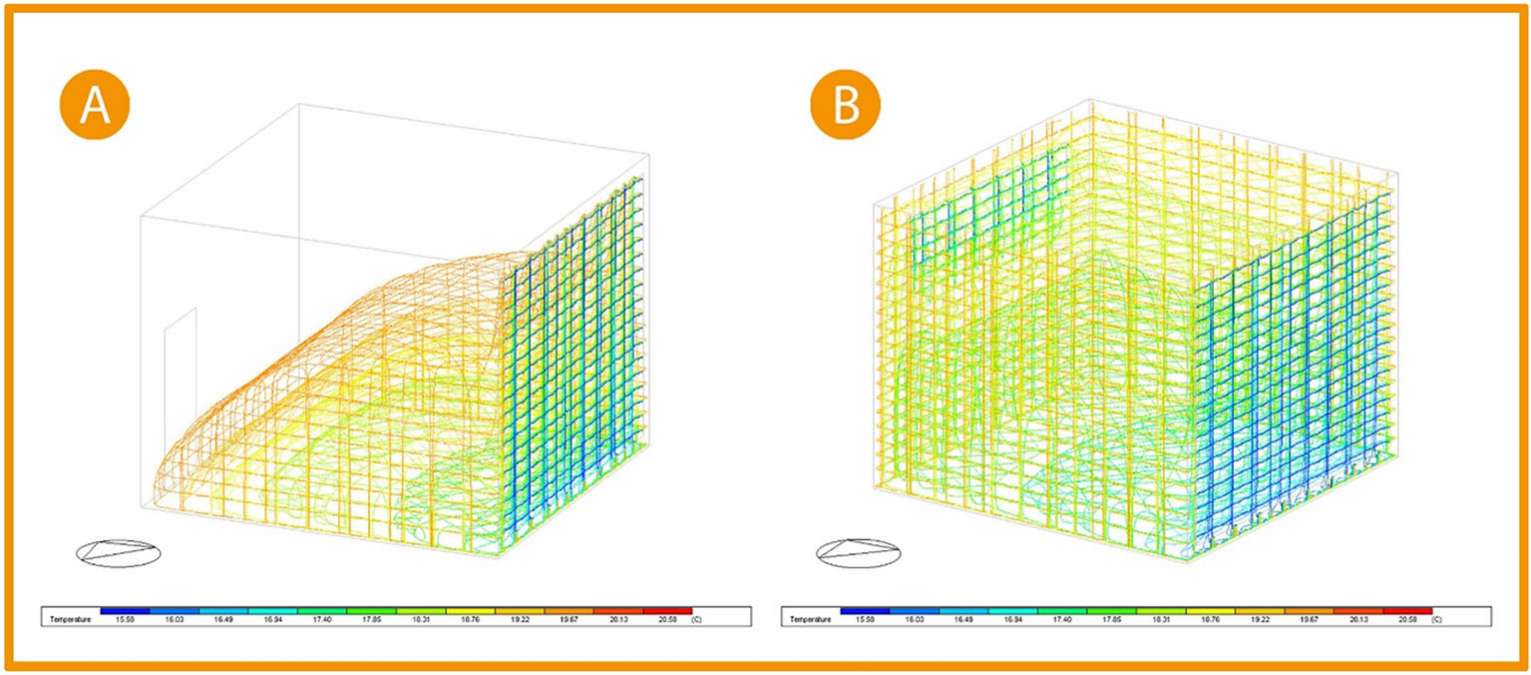
Three-Dimensional thermal distribution comparison chart (A) Base case, (B) Post-implementation. The photos (A) and (B) were acquired from the researcher’s work on the case study office building using DesignBuilder software.

As for the thermal distribution analysis, providing an automatic shading system to the building’s façade along with windows that increased airflow indicated the wider distribution of cooler temperature, leading to thermal comfort. Figure 21, picture (A) shows the three-dimensional thermal distribution in the base case office to cover less area, with the average temperature for user area ranging between 17.85 °C and 19.67 °C (colour indication: orange, yellow, light green, and green). While after implementation in the picture (B), it shows the three-dimensional temperature distribution after modification increases to cover the whole office area leading to an increase in the temperature affected by thermal gain. As the colour indication shows, the orange zone colour decreased (indicating a temperature zone of 19.67 °C), and the usable zone temperature records range between 16.49 °C and 18.31 °C (colour indication: cyan, green, and light green). Thus, the average temperature decreases by 1.37 °C, which is a 7% improvement. ^69^

**Figure 22.**
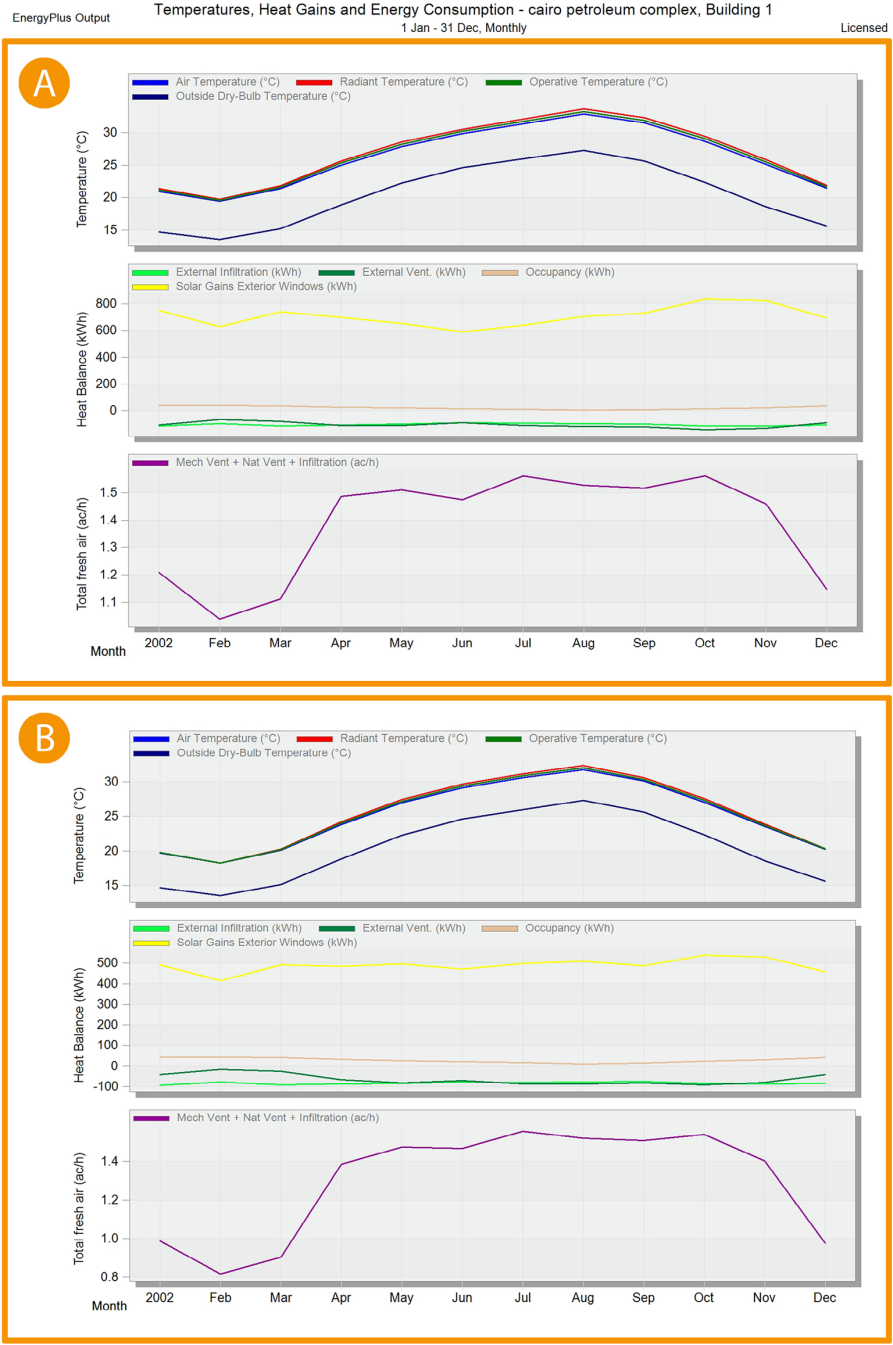
EnergyPlus monthly simulation results for Temperatures, heat gains and energy consumption of the selected office from 1 Jan- 31 Dec (A) Base case, (B) Post-criteria implementation. Showing increased natural ventilation and decreased solar glare and thermal gain. The photos (A) and (B) were acquired from the researcher’s work on the case study office building using the computer software “DesignBuilder” to simulate the changes in the space.

**Figure 23.**
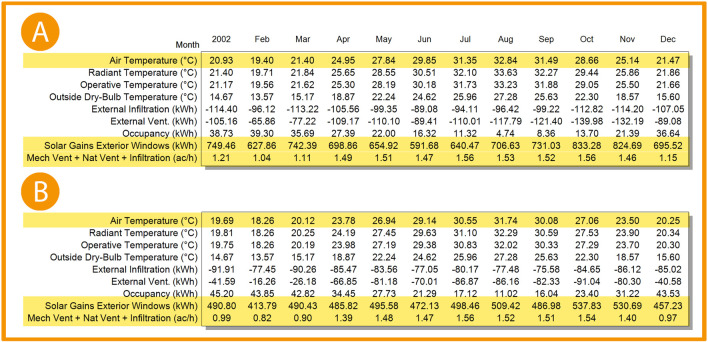
EnergyPlus monthly simulation results for Temperatures, heat gains, and energy consumption of the selected office from 1 Jan- 31 Dec (A) Base case, (B) Post-criteria implementation. Showing the increase in natural ventilation and decrease in solar glare and thermal gain. The photos (A) and (B) were acquired from the researcher’s work on the case study office building using the computer software “DesignBuilder” to simulate the changes on the space.

**Figure 24.**
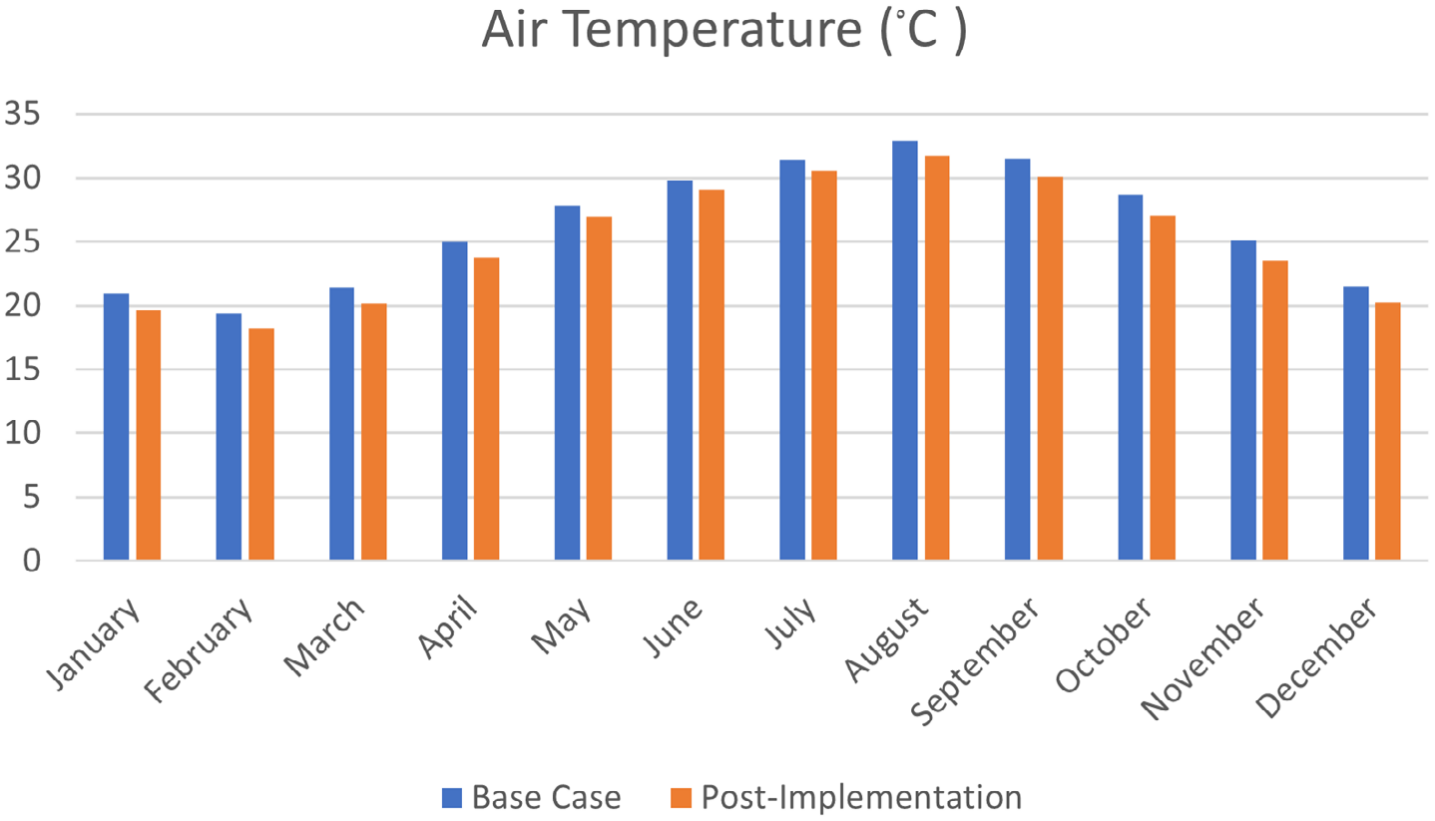
Comparison bar chart between base case and post-implementation Air temperature monthly simulations results.

As shown in Figure 22, Figure 23, and Figure 24, in January and February, air temperature decreased by 5.9%, 6% in March, 4.7% in April, 3.2% in May, 2.4% in June, 2.6% in July, 3.3% in August, 4.5% in September, 5.6% in October, 6.5% in November, and 5.7% in December. ^69^

**Figure 25.**
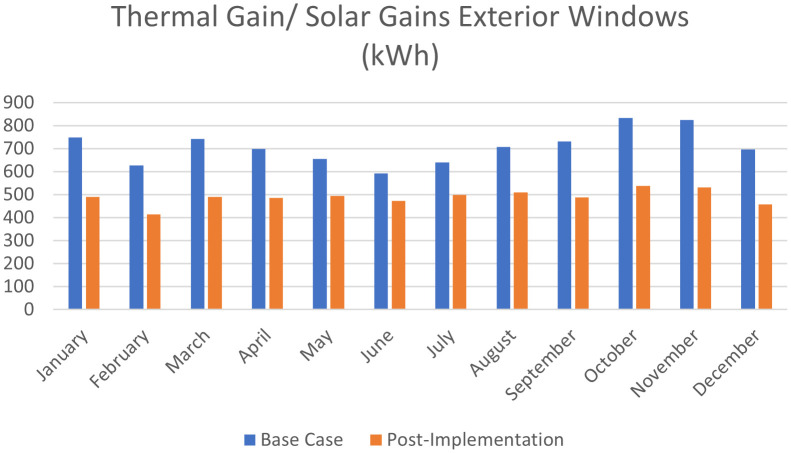
Comparison bar chart between base case and post-implementation monthly Thermal gain/Solar gains exterior windows simulations results.

For the thermal gain/solar gains exterior windows comparison simulation results, January records 34.5% decrease in thermal gain, 34.1% in February, 33.9% in March, 30.5% in April, 24.3% in May, 20.2% in June, 22.1% in July, 27.9% in August, 33.4% in September, 35.5% in October, 35.6% in November, and 34.3% in December ( Figure 22, Figure 23, and Figure 25). ^9^^,^^69^

**Figure 26.**
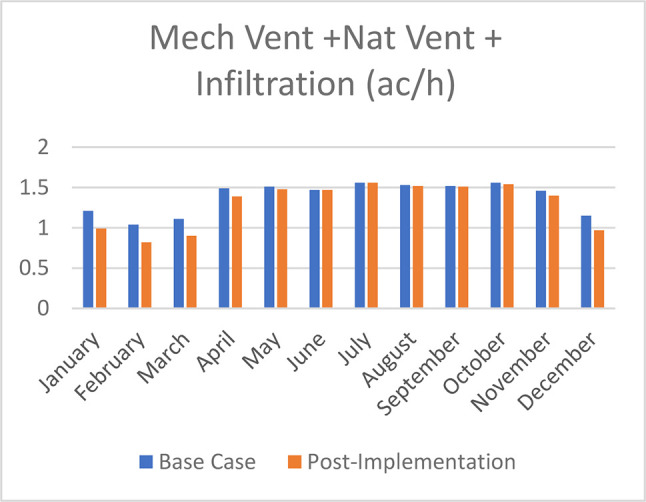
Comparison bar chart between base case and post-implementation of mechanical ventilation, Natural Ventilation, and Infiltration monthly simulations results.

Mechanical Ventilation, Natural Ventilation, and infiltration decreased by 18% in January, 21.2% in February, 19% in March, 6.7% in April, 2% in May, no effect in June and July, 0.65% in August and September, 1.3% in October, 4.1% in November, and 15.7% in December ( Figure 22, Figure 23, and Figure 26).

In addition to providing supplemental light 45 cm above the worksurface to influence employees’ circadian rhythm. According to the future workplace wellness study and other research, it was assured that by achieving thermal comfort, natural ventilation and natural light, an increase in productivity is inevitable. ^17^^,^^69^

## 6. Conclusion and recommendations

Studying WELL Building standards and recognising the success of popular existing office buildings shows great promise for improving occupants’ well-being and productivity. This is especially crucial after the pandemic, as business districts in Egypt must implement these structures to ensure a safe yet productive environment. Regarding present and future circumstances, this kind of research is increasingly vital for establishing better practices in the workplace.

This paper studied successful design criteria for existing office buildings to conduct an easy applicable design criteria for healthy office design to enhance productivity in existing workspace in Egypt.

By studying and analysing WELL Building Rating system concepts. Six design-oriented features with direct impact on employee’s productivity were conducted. Which explained how to achieve adequate levels of natural light, natural ventilation, and thermal comfort in addition to sound and designing for the mind to get employees to thrive in their work area.

Next, by studying office space form evolution, the attributes of productivity in office spaces, and successful office buildings designs like Googleplex building and Amazon spheres, the top three impacting design features over employees’ productivity were conducted. Natural Air, natural light, and thermal comfort. Followed by analysing three WELL certified office spaces and comparing their application of the conducted impacting features, an easy applicable scenario was conducted for implementation on the case study building.

Lastly, by applying the papers findings to the selected case study building, the building simulation results calculated using DesignBuilder computer software showed impact.

### 6.1 Case study findings and conclusion

The simulations showed that by applying the design implementations on the selected case study ( Table 6), the thermal heat gain was reduced after using automatic shading devices by an average of 20.2%-35.6% throughout the year, Airflow increased by 20% after adding 25% user-friendly designed operable windows to the building’s façade and opposite operable windows for cross ventilation. Lastly, adding double-glazed glass for the curtain wall and the automatic shading device enhanced the illuminance distribution, temperature distribution, and air temperature. Direct sunlight area decreased by 9% in January (Winter), 21.9% in April (Spring), 14.3% in July (Summer), and 10% in October (Autumn). Air temperature decreased by a minimum of 2.4% in June and a maximum of 6.5% in November—temperature distribution enhanced by an average of 7%. ^69^

**Table 6.** Conclusion table of the impact of proposed scenario over the case study building: author.

Finding	Application	Impact
**Natural Daylight** (Controlled Glare)	Application of solar sensor exterior shading device over the building façades and interior reflective solar shelves.	- 8.5% decrease in exposure to direct sunlight. - Decrease in the solar glare.
**Circadian Rhythm**	Providing supplemental light at the height of 45 cm above the work surface.	Influences the circadian rhythm of employees to enhance productivity.
**Natural Ventilation** (Interchangeably with HVAC)	- Application of 25% operable window to the office building façades. - Placing a high-level window opposite to the curtain wall windows creates cross ventilation. - Operable skylight to allow natural ventilation	- 20% improvement in single office airflow. - 16.6% decrease in the air velocity of the full building. - Enhanced overall building airflow.
**Thermal Comfort**	- Application of automatic shading device over the building façades. - Providing two heights of operable windows: ○ Higher operable windows for cold weather. ○ Lower operable window for mild/warm weather. - Placing a high-level window opposite to the curtain wall windows and allowing air access from the skylight creates cross ventilation. - Adding double-glazing glass for the skylight.	- 20.2%-35.6% overall decrease in the thermal heat gain throughout the year. - 4.7% decrease in overall air temperature per year. - Enhanced the illuminance distribution, temperature distribution, and air temperature. - Temperature distribution enhanced by an average of 7%.

### 6.2 Research recommendations

This paper has concluded that the top three effective design features on employees productivity which are Air, Light, and Thermal comfort; identified through literature review, example analysis, and testing on a selected medium-sized office building case study, could potentially be applied to an entire existing office building in Egypt. Proposing several criteria aimed at increasing productivity on both the scale of existing office buildings and individual workplaces. These include:

• Ensuring adequate natural ventilation and air quality using windows and cross ventilation, while taking into account the ease of window operation management.
• Providing sufficient natural light, with consideration given to circadian rhythm design elements.
• Utilizing solar sensor exterior shading devices (with device selection taking the façade’s orientation into account) and interior reflective solar shelves that function across all seasons.
• Striving for thermal comfort, appropriate temperature distribution, and strategic furniture placement.
• Enhancing productivity and health through connections to nature.

The paper recommends the use of a productivity measuring method to evaluate the productivity rates of employees in existing buildings, suggesting that these rates could be improved using the proposed scenario. It is recommended that architects and construction firms consider the conducted scenario during the design phase of buildings to enhance occupant performance by advocating for healthy building design.

Furthermore, it is suggested that legislative authorities should be mandated to incorporate conditions that support the health and well-being of occupants into the new construction law for those seeking construction permits. As the existing building code includes provisions related to the sustainability of buildings, it is proposed that several codes be added to ensure the well-being of building occupants. These could include requirements for air quality, such as a minimum number of operable windows in a building and specific sizes for these windows, along with specified HVAC systems with filters to purify air.

Natural light could be regulated by requiring a minimum Window-to-Wall Ratio (WWR) in buildings to ensure adequate sunlight enters the building, the use of skylights, and the implementation of appropriate shading devices that minimize glare without reducing the amount of natural sunlight entering the building.

Thermal comfort could be addressed through requirements for curtain walls and window panels to be at least double-glazed or use other proven methods that allow the entrance of natural light while controlling thermal heat gain. The interchangeable use of HVAC systems and operable windows could be required to achieve thermal comfort, along with thermal zoning and the proper use of indoor and outdoor shading devices.

Finally, the incorporation of plants and water elements both indoors and outdoors could be encouraged to regulate temperature and improve air quality.

## Data availability

### Underlying data

Mendeley data: Well-being as a tool to improve productivity in existing office space. Doi: https://doi.org/10.17632/d5g9vwt28s.1. ^69^

This project contains the following underlying data:

• Adobe Photoshop [Cairo Petroleum Complex architecture plans and cross-sections presentations]
• Autodesk AutoCAD [An architectural detailed plans and cross-section for the Cairo Petroleum Complex office building]
• DesignBuilder [A three-dimensional simulation of the Cairo Petroleum Complex case study building for the base case and the post-implementation of the conducted criteria]

Data are available under the terms of the Creative Commons Attribution 4.0 International license (CC-BY 4.0).

## Footnotes

^1^
Sedentary behavior is distinct from physical inactivity and is characterized as very low-intensity, low-effort activities, such as sitting.

^2^
The correlation coefficient was developed by Karl Pearson in the 1880s based on an idea from Francis Galton, with the mathematical formula established by Auguste Bravais in 1844.

## References

[1] TED: The Economics Daily:2022 [cited 2023 25 March]. Reference Source

[2] United Nations Environmental Programme: Global Status Report 2017 : *Towards a zero-emission, efficient, and resilient buildings and construction sector.* 2017.

[3] Arnett A : *Why is the WELL Building Standard Worth Striving For?* FER: Foodservice Equipment Reports;2021.

[4] Quintana K : *WELL @ Work.* 2017.

[5] Andrade CC Lima ML Devlin AS : Is It the Place or the People? Disentangling the Effects of Hospitals’ Physical and Social Environments on Well-Being. SageJournals. 2016;48(2):299–323.

[6] Tran V Park VD Lee Y : Indoor Air Pollution, Related Human Diseases, and Recent Trends in the Control and Improvement of Indoor Air Quality. Int J Environ Res Public Health. 2020;17(8). 10.3390/ijerph17082927 PMC7215772 32340311

[7] EPA: *Why Indoor Air Quality is Important to schools.* [cited 2023 12 March]. Reference Source

[8] *The total exposure assessment methodology (TEAM) study: Summary and analysis. EPA/600/6-7/002a.* Washington, DC.: U.S. Environmental Protection Agency;1987.

[9] *Indoor Air Quality: What are the trends in indoor air quality and their effects on human health?* The United States Environmental Protection Agency (EPA);2021.

[10] Vaughn E : *Redesigning The Office For The Next 100-Year Flu (Yes, It’s Coming). npr.* 2020.

[11] Kaysen R : *The Post-Pandemic Office.* Architectural Record;2022.

[12] Awada M : Ten questions concerning occupant health in buildings during normal operations and extreme events including the COVID-19 pandemic. Build. Environ. 2021;188: 107480. 10.1016/j.buildenv.2020.107480 PMC9759512 36570375

[13] Bueno AM Paula Xavier AA de Broday EE : Evaluating the Connection between Thermal Comfort and Productivity in Buildings: A Systematic Literature Review. *Buildings.* 2021;11(6):244. 10.3390/buildings11060244

[14] Kaushik A Arif M Tumul P : Effect of thermal comfort on occupant productivity in office buildings: Response surface analysis. Build. Environ. 2020;180: 107021. 10.1016/j.buildenv.2020.107021

[15] Felgueiras F Mourão Z Moreira A : Indoor environmental quality in offices and risk of health and productivity complaints at work: a literature review. J. Hazard. Mater. Adv. 2023: 100314. 10.1016/j.hazadv.2023.100314

[16] Alan K : New Study: Air Quality And Natural Light Have The Biggest Impact On Employee Well-Being. *Forbes.* Forbes.com: Forbes;2019.

[17] Future Workplace, V: *Future Workplace Wellness Study.* research study. 2019 [cited 2023 20 March]. Reference Source

[18] U.S. Bureau of Labor Statistics : *TED: The Economics Daily.*2022 [cited202325 March]. Reference Source

[19] Faragg N : Sick building syndrome and office space design in Cairo, Egypt. *Sage Journals.* 2021;31(2):568–577. 10.1177/1420326X211016507

[20] WHO : Health and Well-Being. Major themes. 1948[cited 2019]. Reference Source

[21] Dunnagan T Peterson M Haynes G : Mental Health Issues in the Workplace: A Case for a New Managerial Approach. *J. Occup. Environ. Med.* 2001;43(12):1073–1080. Reference Source 11765678 10.1097/00043764-200112000-00009

[22] Raderstorf M Kurtz JJAJ : Mental health issues in the workplace: maintaining a productive work force. *AAOHN J.* 2006 Aug;54(8):360–365; quiz 366-367. 10.1177/216507990605400804 16921867

[23] Asumeng M Asamani L Affu J : Occupational safety and health issues in Ghana: strategies for improving employee safety and health at workplace. *International Journal of Business and Management Review.* 2015;3(9):60–79.

[24] Useche SA Montoro L Pérez JIR : Workplace burnout and health issues among Colombian correctional officers. *PLoS One.* 2019;14(2): e0211447. 10.1371/journal.pone.0211447 PMC6372146 30753198

[25] Robroek SJW van de Vathorst S Hilhorst MT : Moral issues in workplace health promotion. *Int. Arch. Occup. Environ. Health.* 2012;85(3):327–331. 10.1007/s00420-011-0675-y 21710278 PMC3299975

[26] IWBI : The WELL Building Standard (WELL). WELL. v2 2018. 2023 [cited 2020]. Reference Source

[27] Allen JG MacNaughton P Satish U : Associations of cognitive function scores with carbon dioxide, ventilation, and volatile organic compound exposures in office workers: a controlled exposure study of green and conventional office environments. *Environ. Health Perspect.* 2016;124(6):805–812. 10.1289/ehp.1510037 26502459 PMC4892924

[28] Boubekri M Cheung IN Reid KJ : Impact of windows and daylight exposure on overall health and sleep quality of office workers: a case-control pilot study. *J. Clin. Sleep Med. * 2014;10(6):603–611. 10.5664/jcsm.3780 24932139 PMC4031400

[29] Seppanen O Fisk WJ Lei Q : Room temperature and productivity in office work. Lawrence Berkeley National Lab (LBNL).Berkeley, CA (United States);2006.

[30] Browning : BJP and Strategy. *Healthier Workplaces, Happier Employees.* 2015;38(3):14.

[31] Vischer JC : The effects of the physical environment on job performance: towards a theoretical model of workspace stress. Journal of the International Society for the Investigation of Stress. 2007;23(3):175–184. 10.1002/smi.1134

[32] Kiara M : 7 best ways to measure productivity of employees, in AboutLeaders. 2023.

[33] Palinkas L : Effects of Physical and Social Environments on the Health and Well-Being of Antarctic Winter-Over Personnel. Environ. Behav. 1991;23(6):782–799. 10.1177/0013916591236008

[34] Helliwell JF Putnam RD : The social context of well-being. *Philos. Trans. R. Soc. Lond. B Biol. Sci.* 2004 Sep 29;359(1449):1435–1446. 10.1098/rstb.2004.1522 15347534 PMC1693420

[35] Larry E James H : The Healthy Building Movement.The Healthy Building Movement.2020[cited 2023]. Reference Source

[36] Wikipedia contributors. Biophilic design. 2023 7August 202316:51 UTC [cited 2023]. Reference Source

[37] Spence C : Senses of place: architectural design for the multisensory mind. *Cognitive Research: Principles and Implications.* 2020;5(1):46. 32945978 10.1186/s41235-020-00243-4 PMC7501350

[38] Park J Rider TR : Facilitating the WELL Building Standard through wellness programs in the workplace. ARCC Conference Repository. 2018.

[39] *The WELL Building Standard (WELL).* WELL v2. 2022 [cited 2020. Reference Source

[40] Beemer CJ : A brief review on the mental health for select elements of the built environment. *Indoor Built. Environ.* 2021;30(2):152–165.

[41] Nigel O Roderic B Michael H : The Future of UK Office Densities. *British Council for offices (BCO).* 2022.

[42] David A : The evolution of modern office buildings and air conditioning. *ASHRAE Journal.* 1999;41(6):1.

[43] Messenger JC Gschwind L : Three generations of Telework: New ICTs and the (R) evolution from Home Office to Virtual Office. New Technology, Work and Employment 2016.31(3):13. 10.1111/ntwe.12073

[44] Conor M : HOW MUCH OFFICE SPACE DO WE NEED PER EMPLOYEE? 2023 Jan. 12 [cited 2023 03/02]. Reference Source

[45] Ernst N : *Neufert Architect’s Data.* fourth edition. Wiley-Blackwell;2012.

[46] Yu R Burke M Raad N : Exploring impact of future flexible working model evolution on urban environment, economy and planning. *J. Urban Manag.* 2019;8(3):10. 10.1016/j.jum.2019.05.002

[47] Mike P : How Much Office Space Do We Need Per Employee?IOffice by Eptura.2020.

[48] Aneta C : *Office Space Planning Guidelines For Returning To Work and a Better Occupant Experience, in Kontakt.io.* Aneta Ciurkot;2022.

[49] Robert L : Office Sprawl: The Evolving Geography of Business. Center on Urban & Metropolitan Policy,2000.

[50] Sarah O :2022; *BCO recommends allocating more space-per-person for the post-pandemic office* . FMJ: Facilities Management Journal.

[51] WSP : *How Will Covid-19 Change Demand For Office Space?* WSP;2022.

[52] WorldGBC : Principle 1: Protect and Improve Health. 2022 [cited202312 Feb]. Reference Source

[53] WorldGBC : Health Wellbeing & Productivity in Offices.2014, World Green Building Council (WorldGBC): World Green Building Council.

[54] Haiken: *What are the 5 key elements to a good office design?* Haiken;2021.

[55] L’Estrange S : A Functional Post-Pandemic Office That Inspires. *Workdesign Magazine.* 2021.

[56] Blaine B : rethinking office design trends in a post-covid world. *Architect Magazine.* 2020.

[57] Daisuke W : Google’s Plan for the Future of Work: Privacy Robots and Balloon Walls, in NewYork Times. 2021. *NewYork Times.*

[58] Jessica S : The Incredible Science Behind Why Amazon Filled Its New Office With 40,000 Plants. *Design.* 2018; [cited 2022]. Reference Source

[59] Craig W : See the Offices Where Employees Can Work in a Tree House. *Environment.* 2018; [cited 2022]. Reference Source

[60] *Googleplex*, in *wikipedia.* wikipedia wikipedia.

[61] Tudorache A : *The ultimate level of employee satisfaction: Working at the Googleplex.*, in *Performance Magazine.* The KPI Institute;2013.

[62] Wikipedia: Amazon Spheres. *Wikipedia. Wikipedia: Wikipedia.*

[63] WenMing Y Miro E : Inference at the Edge: A Case Study at the Amazon Spheres, in solaripedia. 2018.

[64] Meister JC : The #1 Office Perk? Natural Light. *Harvard Business Review.* 2018.

[65] PHIPPS Conservatory : *Achieving the WELL Building Standard.* Green Building Toolkit Series I. 2015.

[66] The ASID Foundation : ASID HQ office research. American Society of Interior Designers(ASID) 2017 [cited 2023; Reference Source

[67] Architectural Health : Yang Architects LLC. Healthy Building Walkthrough: The ASID Headquarters in Washington, D.C.2023 [cited 2023]. Reference Source

[68] Katie A. : 425 Park Avenue telescopes skyward with diagrid glass.2021. [cited 2022]. Reference Source

[69] Hamadah M : Well-being as a tool to improve productivity in existing office space. *Mendeley Data.* 2023. 10.17632/d5g9vwt28s.1 PMC10797229 38249134

[70] Software, A.a.M: *AgriMetSoft.* R2 (correlation coefficient). 2019 [cited 2023 6 april]. Online Calculators. Reference Source

