# Supplementary material for: Well-being as a tool to improve productivity in existing office space: Case study in Alexandria, Egypt
Source: F1000Res. 2023 Dec 14;12:639. Originally published 2023 Jun 12. [Version 2] doi: 10.12688/f1000research.133199.2 (PMC10797229; doi:10.12688/f1000research.133199.2)
Supplement: Peer review history

# Reviewer report: Reviewer response for version 2

**DOI:** 10.5256/f1000research.157534.r235686

**Published:** 2024-01-18

**Authors:** Elina Mohd Husini

**Affiliations:** 1 University Sains Islam Malaysia, Negri Sembilan, Malaysia

The authors have briefly discussed on the method. In a way to explain this, it is suggested that the authors have to describe the significance of WELL building rating system compared to other systems to validate the impact on productivity. The authors should provide a chart to explain the features and factors to analyze.

 The simulation data is suggested to be presented in a chart and the max/min  of lux based on factors needed; orientation, position of the office room/  exterior/interior/.

 The results shall provide the summary, as there are many factors presented in the research. Tables/charts are needed to present in the summary of the result.

Is the case presented with sufficient detail to be useful for teaching or other practitioners?

Partly

Is the work clearly and accurately presented and does it cite the current literature?

Yes

If applicable, is the statistical analysis and its interpretation appropriate?

Yes

Are all the source data underlying the results available to ensure full reproducibility?

Partly

Are the conclusions drawn adequately supported by the results?

Yes

Is the background of the case’s history and progression described in sufficient detail?

Partly

Reviewer Expertise:

Sustainable Architecture/ Design/ Daylighting/ renewable energy

I confirm that I have read this submission and believe that I have an appropriate level of expertise to confirm that it is of an acceptable scientific standard, however I have significant reservations, as outlined above.

# Reviewer report: Reviewer response for version 2

**DOI:** 10.5256/f1000research.157534.r235692

**Published:** 2024-01-18

**Authors:** Hasim Altan

**Affiliations:** 1 Department of Architecture, Prince Mohammad Bin Fahd University, Al Khobar, Eastern Province, Saudi Arabia

The paper recommends the use of a productivity measuring method to evaluate the productivity rates of employees in existing buildings, suggesting that these rates could be improved using the proposed scenario as well as during the design phase of buildings to enhance occupant performance by advocating for healthy building design.

 The paper is relevant to the scope of the journal and is showing some originality. It appears to be a complete work after some significant revision presenting interesting findings. The paper is well written and structured with satisfactory literature review and comprehensive analyses. It has both clarity and good expression in English, and is in appropriate length with concise conclusions. I do not have any further comments or any criticism of this paper, and therefore, in my opinion, it should be accepted without any hesitation.

Is the case presented with sufficient detail to be useful for teaching or other practitioners?

Yes

Is the work clearly and accurately presented and does it cite the current literature?

Yes

If applicable, is the statistical analysis and its interpretation appropriate?

Not applicable

Are all the source data underlying the results available to ensure full reproducibility?

Yes

Are the conclusions drawn adequately supported by the results?

Yes

Is the background of the case’s history and progression described in sufficient detail?

Yes

Reviewer Expertise:

sustainable design of the built environment

I confirm that I have read this submission and believe that I have an appropriate level of expertise to confirm that it is of an acceptable scientific standard.

# Reviewer report: Reviewer response for version 2

**DOI:** 10.5256/f1000research.157534.r230086

**Published:** 2024-01-18

**Authors:** Mark Mulville

**Affiliations:** 1 School of Surveying and Construction Innovation, Technological University Dublin, Dublin, Leinster, Ireland

I am happy for it to be approved. The authors have made significant improvements, I still believe the link made between income and the building is not sound as there are too many external variables at play and there could still be more depth of analysis etc., but overall it is satisfactory.

Is the case presented with sufficient detail to be useful for teaching or other practitioners?

Yes

Is the work clearly and accurately presented and does it cite the current literature?

Partly

If applicable, is the statistical analysis and its interpretation appropriate?

Yes

Are all the source data underlying the results available to ensure full reproducibility?

Partly

Are the conclusions drawn adequately supported by the results?

Yes

Is the background of the case’s history and progression described in sufficient detail?

Partly

Reviewer Expertise:

NA

I confirm that I have read this submission and believe that I have an appropriate level of expertise to confirm that it is of an acceptable scientific standard.

# Reviewer report: Reviewer response for version 1

**DOI:** 10.5256/f1000research.146177.r183493

**Published:** 2023-07-10

**Authors:** Mark Mulville

**Affiliations:** 1 School of Surveying and Construction Innovation, Technological University Dublin, Dublin, Leinster, Ireland

Abstract is clear and concise, the 'methods' section here could include (in brackets) what specific data is collected. There are too many key words - 5-6 is plenty (unless journal requires more). 

 There is a clear rationalle for research of this type - it highlights the disjointed approach to building design vs building operation and the reality of occupant satisfaction/comfort/productivity/health.

 Some of the sentences could be revised for better flow, i.e. first sentence in section 1.0. The work could generally do with a review to ensure the flow of writing is easy to follow. Some of the sentences in the work are very long - try to stick to a maximum of 40 words in the sentence.

 Paragraph three in section 1.0 the first sentence needs to be supported with a reference, likewise first sentence in paragraph four - the work should be reviewed to ensure all such statements are fully referenced. 

 Section 1.2 is it just Architects and Construction Firms? I would think the entire design team needs to be involved. Construction firms will build what they are instructed to (as long as it meets relevant requirements). Building Services Engineers will be important in this as will those who set regulations/policy.

 Section 1.3 how many office buildings in Alexandria? 3? On what basis where they chosen? Do you have permission to name the specific buildings (if they are the case studies used) - this is potentially a significant issue - my approach would be to describe the type (i.e. a high end cooperate head quarters, of x size, design etc, built in xxxx.)

 I would like to see section 1.3 revised to add clarity - perhaps a diagram would be of help, I currently find it difficult to follow the methods. Is it three case study buildings or four? on what basis where the specific criteria to be evaluation chosen?

 Methodology and method mean different thinks, but are used interchangeably here

 I would like to see more detail on how the data collection was carried out - it would not be possible to gather all of this information through a walk through, what 'other sources' were used? 

 Sometimes in the work past tense is use then present tense in the next sentence, this could be revised to help the flow of the work.

 In terms of the model creation, more detail on data collection is needed to ensure the reader understands the level of accuracy. The overall section needs revision as I am not clear why one model was built when there seems to be multiple case studies?

 Was any monitoring of actual environmental conditions in the building(s) carried out?

 Section 1.4 should be earlier in the work

 Section 2.0 is titled 'results' but in reality this is a literature review - consider renaming this section

 I do not understand the statement in section 2.2 that the building increased its revenue in 2013 - this linkage between the design and revenue generation seems tenuous as it is presently presented.

 There is a good deal of literature highlighting the importance in indoor air quality, light, noise, configuration, privacy etc and the impact on occupant health, wellbeing, productivity (which the WELL standard was developed as a result of). At present there is, in my view, a lack of depth in the literature review in relation to these factors. Some interesting case study buildings are presented, but the link between their design and the wellbeing/health/productivity benefit and reason for this is not fully explored. There is quite a bit of descriptive text in relation to the WELL standard - it would be of greater benefit to utilise the literature to demonstrate why specific factors are of importance.

 Provide the sources and a rationale for the chosen design criteria in table 4.

 Is it a single case study building being used? the methods section seemed to suggest multiple case studies. Is the office building in section 4.2 the one that was modelled in DeisgnBuilder? the description of the building may be better placed in the methods section.

 Section 4.4 may also better fit in the methods section.

 Some of the data described in section 4.6 would work better in a table.

 The modelling work is interesting, but I believe the data could be better presented to max it easier to follow/compare. In the text then the focus could be on the implications etc.

Is the case presented with sufficient detail to be useful for teaching or other practitioners?

Yes

Is the work clearly and accurately presented and does it cite the current literature?

Partly

If applicable, is the statistical analysis and its interpretation appropriate?

Yes

Are all the source data underlying the results available to ensure full reproducibility?

Partly

Are the conclusions drawn adequately supported by the results?

Yes

Is the background of the case’s history and progression described in sufficient detail?

Partly

Reviewer Expertise:

Building performance evaluation

I confirm that I have read this submission and believe that I have an appropriate level of expertise to confirm that it is of an acceptable scientific standard, however I have significant reservations, as outlined above.

# Response

**Published:** 2023-10-18

**Authors:** Miral Hamadah

Dear Dr. Mark,

 Thank you for your detailed feedback and constructive criticism. We appreciate your time in helping to improve the manuscript. First, i would like to clarify that the revised manuscript sections has been rearranged for better flow and clarification. In response to your suggestions, the following major revisions have been made:

 1. Regarding the clarity and conciseness of the abstract. In response to your suggestion, I have now amended the 'methods' section of the abstract to include specific details about the data collected in this study.

 2. Regarding the keywords, I acknowledge your recommendation for a more focused selection. The list has been revised and narrowed down to six keywords that accurately reflect the core themes of the paper. However, I would like to note that the initial extensive list was due to certain journal requirements.

 3. Regarding the flow of the manuscript and the length of the sentences. In response to your comments, I have revisited the document and made necessary revisions, particularly focusing on the first sentence of section 1.0 and other similar instances throughout the text. The aim was to improve the overall readability and ensure that the narrative is easier to follow.

 Moreover, I have tried to adhere to your recommendation of keeping sentences within a maximum limit of 40 words.

 4. Regarding referencing in the manuscript. I understand the importance of properly citing all statements that are not common knowledge, and I appreciate your attention to detail in this regard.

 In response to your feedback, I have thoroughly reviewed the document, particularly focusing on paragraph three in section 1.0 and the first sentence in paragraph four. References have been added wherever necessary to support the statements made.

 5. Section 1.2. I concur with your observation that the entire design team, not just architects and construction firms, plays a crucial role in the process.

 As per your suggestion, the section has been revised to reflect this broader perspective. It now emphasizes the importance of collaboration amongst all stakeholders, including Building Services Engineers and regulators, who indeed have significant contributions to make.

 6. In response to your comments, I have revised Section 1.3 to clarify that the case study pertains to a single office building in Egypt, not three or four as may have been previously misinterpreted. This building was chosen as it exemplifies typical modern office buildings in Egypt, especially those with prevalent design issues. To address your concerns about confidentiality, the specific name of the building has been removed from the paper. Instead, it is now referred to in a more general way that preserves anonymity while still providing necessary context.

 7. I have expanded and modified section 1.4 Methods to provide more detail about the process. 

 8. In response to your feedback, I have revised the section on model creation to provide more explicit details about the data collection process. This should enhance the reader's understanding of the level of accuracy involved in our study.

 To address your query, the case study pertains to a single office building in Egypt, not multiple buildings. The reason for this is that the selected building represents typical modern office buildings in the region, making it an ideal subject for our analysis. Any other examples mentioned in the paper were used to help formulate the scenario, but were not themselves subjects of the case study.

 Moreover, as part of our data collection process, we did conduct monitoring of actual environmental conditions in the building ( Indoor Air temperature). This was essential in validating our base-case building model results. thus, the paper was modified to clarify this process.

 9. As per your request section 1.3 and 1.4 were switched . Along side other arranging in the paper for clarity purpose and better information flow.

 10. The title of Section 2  has been modified as per your suggestion.

 11. The reference to increased revenue in 2013 for the Googleplex building is an indirect way of indicating enhanced employee productivity, which is a central theme of our study and Revenue calculation is a way of calculating productivity which was explained in the paper as one of productivity measuring methods. Successful design features in buildings, like those in Googleplex and Amazon Spheres, have been shown to boost employee productivity, and by extension, revenue.

 However, I agree that the link between design and revenue generation needed clearer explanation. Therefore, I have revised this section to better elucidate the relationship between the two. The focus is on understanding how specific design features can impact productivity, and consequently, the economic success of a company.

 This understanding is pivotal to our study's aim of identifying design-oriented features that can be effectively implemented in office buildings in Egypt to enhance productivity. 

 12. I agree that the literature review could benefit from a more in-depth exploration of the factors affecting occupant health, wellbeing, and productivity. In light of your comments, I have revised the literature review to delve further into these aspects.

 13. as for the rationale for Table 4. A comparative study that resulted in the design criteria has been added to the revised paper as Section 4.

 14. A description of the case study office building was placed in the methods section for clarification.

 15. Modifications to the case study section was made and additional figures was added for clarifications.

 We highly value your insightful suggestions, which have contributed significantly to refining the manuscript.

 Best Regards, Miral H.

# Reviewer report: Reviewer response for version 1

**DOI:** 10.5256/f1000research.146177.r183497

**Published:** 2023-07-05

**Authors:** Osama Omar

**Affiliations:** 1 Faculty of Architecture‐Design and Built Environment, Beirut Arab University, Beirut, Lebanon

Dear Authors,

 I would like to thank the author/s for their effort.

 A significant effort has been made by the authors to improve the design strategies in Egypt to increase productivity through concluding a checklist to be implemented in Egypt office spaces designed to improve productivity and well-being of employees and implement it in the case study office. But there are some minor comments that need from authors to modify it to enhance the research:

1. In Abstract:  I prefer if the author can rewrite the Methods with more summarized and clarification for what he/ she mentioned in page 3 & 4.
2. In Keywords, The authors should be more specific and presize when select the keywords. For Example, post-pandemic office buildings design standards its too long to be a keyword. For that, I prefer if the author can change the keywords to be like the following: Well Standard, Sick Building Syndrome, Occupant`s Productivity, well–being framework, Office Building Design.
3. In the Introduction: it`s so brief and short. So, I prefer to expand the references in this area especially in Introduction with more updated references related to the topic specially when it come to the references, most of references exceed more than 10 years.
4. This paper focuses only on Well Building Rating System and ignores Pyrmid Rating System, so I recommend the author add a comparison part in the introduction and mention the missing parts between them.
5. Due to the different weather and architecture features in Egypt, the selected examples on page 8 & 9 are not relevant to the case study in Egypt. In that case, I suggest the author remove them from the paper and keep Tables 1, 2 &3 as a summary of design features from international examples.
6. It would be better if the author provide the WELL Check list criteria as mention in page 13 (10 core concepts) for Building Certification. Also, Its more important to clarify for the reader why the authors focus on just six concept as mention in first line in page 4.
7. In page 27, 28 the word conclusion were repeated twice in section 5.1 and section 5.2.

By the end, I want to say that you did a great job and all this comments is to encourage the author to enhance the quality of paper.

 Thank you for your efforts.

 Best Wishes

Is the case presented with sufficient detail to be useful for teaching or other practitioners?

Yes

Is the work clearly and accurately presented and does it cite the current literature?

Yes

If applicable, is the statistical analysis and its interpretation appropriate?

Yes

Are all the source data underlying the results available to ensure full reproducibility?

Yes

Are the conclusions drawn adequately supported by the results?

Partly

Is the background of the case’s history and progression described in sufficient detail?

Yes

Reviewer Expertise:

Environmental Studies, Sustainable Design, Zero Energy Buildings , Smart Cities, Intelligent Buildings, Sick Building Syndrome

I confirm that I have read this submission and believe that I have an appropriate level of expertise to confirm that it is of an acceptable scientific standard, however I have significant reservations, as outlined above.

# Response

**Published:** 2023-10-18

**Authors:** Miral Hamadah

Dear Dr.Osama,

 we are grateful for the time and effort you've invested in providing such comprehensive and insightful feedback on our manuscript. Your comments have been instrumental in improving the quality of the paper, and we appreciate your suggestions.

1. In response to your comment about the abstract's Methods section, I have made revisions for more clarity and succinctness. However, I would like to note that the brevity was necessitated by the 300-word limit on the abstract, which compelled a more concise approach.
2. I have accommodated your feedback regarding the specificity of the keywords. The suggested terms 'Well Standard', 'Sick Building Syndrome', 'Occupant`s Productivity', 'well–being framework', and 'Office Building Design' have now been incorporated into the revised manuscript.
3. Following your advice, I have added more recent references related to the subject matter, enhancing the paper's relevance and depth.
4. As for the focus on the WELL Building Rating System, it was chosen due to its emphasis on well-being design features impacting workforce productivity and being a complementary system to other green building rating systems. The Green Pyramid Rating System, while valuable, wasn't included because of its lesser adoption by buildings in Egypt, making it challenging to study, and as other green building rating systems does not necessarily focus on aspects directly impacting productivity. However, I value your suggestion and will contemplate including a comparative analysis in future research. As the aim of this paper was to maintain a clear and focused narrative on the influence of WELL's well-being design features on productivity, which is why additional information on other green building systems was omitted.
5. The examples of Googleplex and Amazon Spheres were chosen for their recognized success in office building design. While these examples may not directly correspond to Egypt's architectural and weather conditions, they offer valuable insights that can be adapted to various contexts. Nevertheless, I have made modifications to Tables 1 and 2 for better clarity.
6. I have elaborated on the WELL Building Standard's 10 core concepts in response to your feedback. A detailed explanation of why only six of these concepts were focused upon has been provided in the revised manuscript. These specific concepts were selected due to their design-oriented nature and direct impact on workforce productivity, making them integral elements in the context of this research. In response to the suggestion about providing a complete WELL Building Standard checklist, it is important to note that due to its extensive nature, incorporating the full list directly into the paper might disrupt the flow and conciseness of the content. However, I acknowledge that this information could be beneficial for the comprehensive understanding of the topic. Thus, I recommend referring to the official WELL Building Standard website which has the complete and most updated checklist and can be easily accessed ( https://v2.wellcertified.com/en/wellv2/overview/) for a comprehensive understanding.
7. Lastly, the repetition in subtitles was an oversight and has been corrected in the revised manuscript following your keen observation.

Once again, thank you for your perceptive feedback. Your comments have significantly contributed to improving the paper's quality and coherence.

 Best Regards.

 Miral H.
